# Comparative transcriptome analysis reveals insights into the streamlined genomes of haplosclerid demosponges

**DOI:** 10.1038/srep18774

**Published:** 2016-01-07

**Authors:** Christine Guzman, Cecilia Conaco

**Affiliations:** 1 Marine Science Institute, College of Science, University of the Philippines, Diliman, Quezon City, Philippines 1101.

## Abstract

Sponges (Porifera) are one of the most ancestral metazoan groups. They are characterized by a simple body plan lacking the true tissues and organ systems found in other animals. Members of this phylum display a remarkable diversity of form and function and yet little is known about the composition and complexity of their genomes. In this study, we sequenced the transcriptomes of two marine haplosclerid sponges belonging to Demospongiae, the largest and most diverse class within phylum Porifera, and compared their gene content with members of other sponge classes. We recovered 44,693 and 50,067 transcripts expressed in adult tissues of *Haliclona amboinensis* and *Haliclona tubifera*, respectively. These transcripts translate into 20,280 peptides in *H. amboinensis* and 18,000 peptides in *H. tubifera*. Genes associated with important signaling and metabolic pathways, regulatory networks, as well as genes that may be important in the organismal stress response, were identified in the transcriptomes. Futhermore, lineage-specific innovations were identified that may be correlated with observed sponge characters and ecological adaptations. The core gene complement expressed within the tissues of adult haplosclerid demosponges may represent a streamlined and flexible genetic toolkit that underlies the ecological success and resilience of sponges to environmental stress.

## Introduction

Sponges are important components of coral reef ecosystems. They can filter large amounts of seawater and are central players in bentho-pelagic coupling and cycling of nutrients^1^,^2^. They also contribute to substrate modification through bioerosion and consolidation of reef structures while providing unique habitats for various marine organisms and microorganisms^3^. These important functional roles exert major impacts on the overall functioning of marine ecosystems. Sponges have also become important model organisms for the understanding of early animal evolution and the emergence of multicellularity, as well as for their capacity for regeneration, biomineralization, and their unique chemical defense mechanisms^4^. However, despite their evolutionary significance and ecological impact on marine habitats, sponges remain poorly investigated and appreciated.

Sponges arose more than 600 million years ago and are considered one of the most ancestral metazoan groups^5^,^6^. Their simple body plan has proven effective and has remained largely unchanged over time. A typical sponge body consists of three distinct functional layers, the pinacoderm (exterior), mesohyl (middle) and choanoderm (interior) around an intricate system of water canals, choanocyte chambers, incurrent pores (ostia) and excurrent pores (oscules)^7^. The skeletal elements of sponges can be comprised of inorganic spicules (either siliceous or calcareous), proteinaceous spongin, chitin and collagen^8^. Unlike other animals, sponges do not have a centralized gut nor conventional muscles and nerves^8^. Sponge tissues house a dense and varied community of prokaryotic, as well as eukaryotic, symbionts that contribute to the metabolic complexity of the holobiont^9^.

Despite the diversity within phylum Porifera, the genetic potential within this group is largely understudied. Analysis of sponge species representing the major classes within Porifera reveals the surprising genetic complexity of sponges and debunk the view that morphologically simple animals have simple genomes. Sponges possess a genetic toolkit corresponding to key metazoan features, such as cell adhesion, cell cycle control, tissue differentiation, apoptosis, innate immunity and development^4^. Surprisingly, animal-associated genes that were previously thought to have evolved only in the last common ancestor of cnidarians and bilaterians have been discovered in some sponges. This suggests that the last common ancestor of metazoans already possessed a complex genome that conferred the capacity to sense and respond to the environment while maintaining multicellular homeostasis^4^,^10^,^11^,^12^,^13^. The difference in the genomic content between basal metazoans and the different sponge lineages is driven by gene loss and gene family expansion events that occurred after their divergence from the last common ancestor of metazoans^4^,^10^,^13^.

Porifera can be divided into four major classes based on skeleton and tissue composition. These classes are the Homoscleromorpha, Calcarea, Hexactinellida, and Demospongiae. Demospongiae are by far the most diverse in terms of number of species, abundance, and ecosystem distribution. Of the 8,553 species of modern sponges, 83% belong to the demosponge class^14^. Demosponges exhibit ecological adaptability and plasticity in response to different environmental factors, such as light, temperature, turbidity and hydrodynamics, allowing them to integrate into diverse habitats from marine to freshwater, intertidal to deep seafloors, even in caves and polar seas^14^,^15^. Many demosponge species have emerged as ‘winners’, thriving in extreme environments, exhibiting resilience to environmental disturbances, and successfully outcompeting other organisms^16^. Unfortunately, taxonomic classification of demosponges remains a major challenge because of deep divergence between major clades, which hinders phylogenetic resolution at various levels^17^.

In this study, we sequenced the adult transcriptomes of two marine haplosclerid demosponges and compared their gene content to other sponge classes. Our findings point to the similarity of the genetic complement of closely related haplosclerid demosponges and highlight some differences that may underlie the unique characteristics of each species. These genetic differences may be hallmarks of the ecological requirements and adaptive potential of each sponge.

## Results and Discussion

This study generated transcriptome data using Illumina sequencing for adult tissues of two non-model *Haliclona* species belonging to order Haplosclerida of class Demospongiae. Comparison to other sponge genomes and transcriptomes reveals that haplosclerid demosponges possess a similar core genetic repertoire despite broad differences in morphology and ecology.

### Sponge characterization

**Figure 1:**
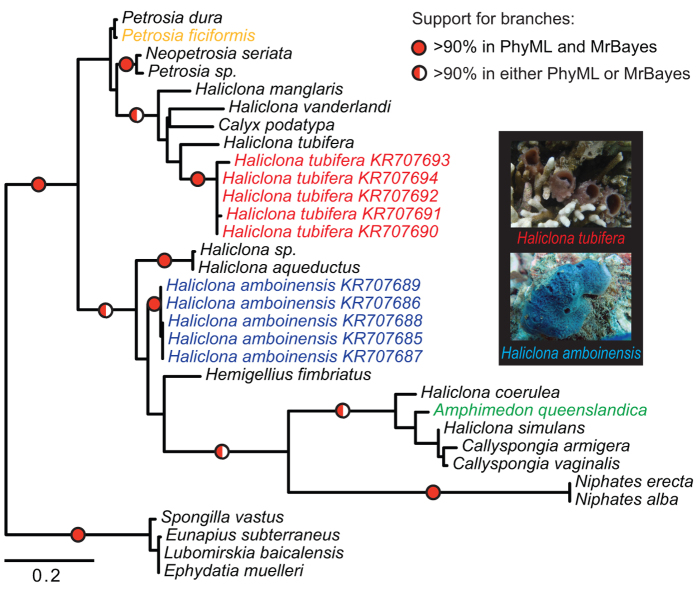
Phylogenetic analysis of haplosclerid sponges using the COI gene. *H. amboinensis* is an encrusting sponge that is found attached to hard substrates at depths of 7–10 meters while *H. tubifera* is a soft tubular sponge found in association with coral skeletons in shallow reef flats. *H. amboinensis* groups with members of family Niphatidae, including *A. queenslandica*, while *H. tubifera* groups more closely with the Petrosidae. Node support values are derived from bootstrap replicates for the maximum likelihood method, as well as posterior probability for Bayesian analysis. Red circles at the nodes represent >90% support in both maximum likelihood and Bayesian analyses while half-filled circles represent >90% support from one method only. The tree is rooted on the freshwater haplosclerids *E. muelleri*, *E. subterraneus, S. vastus* and *L. baicalensis.* Genbank accession numbers for *H. amboinensis* and *H. tubifera* COI sequences are indicated.

*Haliclona amboinensis* and *Haliclona tubifera* are classified as haplosclerid demosponges under family Chalinidae yet exhibit very different morphological and ecological characteristics. *H. amboinensis* (Fig. 1, Supplementary Figure 1) is a blue-purple encrusting sponge with a broad depth range. It can be found attached to hard substrates, such as rocks or corals. The surface of this sponge is rough to the touch with no visible ostia and its texture is brittle or crumbly. Its skeleton is characterized by an isotropic reticulation formed by oxeas, which are either straight or curved at the center^18^. Microcleres such as c-shaped sigmas can also be found in this species. *H. tubifera* (Fig. 1, Supplementary Figure 1) is a soft pink, occasionally light brownish, tubular sponge found in association with coral skeletons in shallow water reef flats. This sponge has a hispid surface, is spongy and compressible in texture. *H. tubifera* resembles *Haliclona sinyeoenesis*^19^ in its growth form, shape and choanosomal skeleton. However, *H. tubifera* has one size of oxea, while *H. sinyeoensis* has oxea of two different sizes (Supplementary Table 1).

### COI phylogeny

Order Haplosclerida is the largest group within class Demospongiae. It consists of three main suborders, Haplosclerina, Petrosina (both marine) and Spongillina (freshwater). Based on traditional morphological cladistics, *H. amboinensis* and *H. tubifera* are classified within family Chalinidae of suborder Haplosclerina, while *Amphimedon queenslandica* belongs to family Niphatidae within the same suborder^20^. Cytochrome oxidase I (COI) gene sequencing, however, reveals that members of genus *Haliclona* are interspersed into different subclades within Haplosclerida. *H. amboinensis* COI sequences cluster with both Chalinidae and Niphatidae sponges (*Niphates* and *Amphimedon*) while *H. tubifera* sequences cluster with *Petrosia* of suborder Petrosina. In previous studies, *H. tubifera* was found to be closely related to other niphatids, such as *Neopetrosia* and *Xestospongia,* and such species were positioned within the same clade as *A. queenslandica*^21^. However, inclusion of sequences from *H. amboinensis* reveals that this species has greater affinity with the niphatid sponges compared to *H. tubifera*. Our COI tree is congruent with recent phylogenetic studies that support the monophyly of marine haplosclerids but fail to resolve most families and their two suborders, Haplosclerina and Petrosina, as monophyletic^21^,^22^. It should be noted, however, that the 5′ end of the COI gene has been shown to evolve more slowly in sponges and thus may not always be able to finely resolve their phylogenetic relationships^23^. Nonetheless, COI phylogeny is recapitulated by phylogenetic analysis based on sequences of nuclear encoded genes, such as the membrane-associated guanylate kinases, DLG and MAGI (Supplementary Figure 2).

### Transcriptome sequencing and *de novo* assembly

Barcoded cDNA libraries with an average insert size of 319 bp were constructed using the Illumina TruSeq RNA sample prep kit. Libraries were sequenced on the Illumina HiSeq 2000 platform to generate an average of 53 million clean paired-end reads per library with a read length of 100 bp. Trinity *de novo* assembly rendered 107,470 and 124,476 total transcripts for *H. amboinensis* and *H. tubifera*, respectively (Supplementary Table 2). After clustering of transcripts to reduce assembly redundancy, 44,693 and 50,067 transcripts were retained for *H. amboinensis* and *H. tubifera*, respectively. Longer transcripts were assembled for *H. tubifera* (N50 = 1,583 bp) compared to *H. amboinensis* (N50 = 1,527 bp). Based on these assembly statistics, our transcriptomes are of comparable quality to recently published sponge transcriptomes^13^.

Protein coding regions within the non-redundant reference transcipts were identified. About 35–45% of total assembled transcripts could be translated into proteins, suggesting that the reference transcriptome assemblies may still include non-protein coding sequences, as well as truncated or potentially misassembled sequences (Supplementary Table 2). More complete open reading frames (ORFs) were recovered from the *H. tubifera* compared to the *H. amboinensis* assembly. Retaining only the longest ORF for each transcript returned 20,280 and 18,000 reference peptides for *H. amboinensis* and *H. tubifera*, respectively.

### Transcriptome annotation

**Figure 2:**
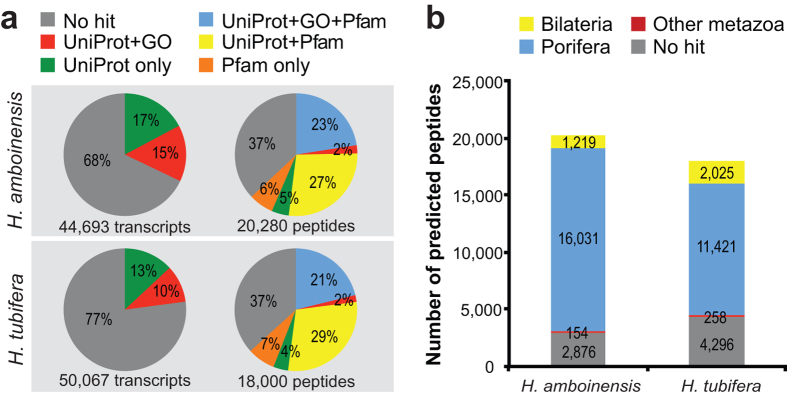
Transcriptome annotation and comparison with other sponges. (**a**) Percent of assembled transcripts with sequence similarity to UniProt peptides and gene ontology (GO) annotation. Assembled transcripts were aligned by Blastx to proteins in the UniProt database with an e-value cutoff of 1 × 10^−5^. The GO annotation associated with the top UniProt hits were retained for each transcript. Longest ORFs from *H. amboinensis* (*Hamb*) and *H. tubifera* (*Htub*) were predicted using TransDecoder and were aligned by Blastp to the UniProt database. The percent of peptides with matches in UniProt, GO annotation, and Pfam domains are shown. (**b**) Percent of predicted peptides aligning to sequences in the Ensembl Metazoa database.

To annotate the reference transcriptomes, transcripts were aligned by Blastx against the UniProt database with an e-value cutoff of 1 × 10^−5^ (Supplementary Table 3 and Supplementary Figure 3). Gene ontology terms were assigned based on the top Blastx hits. 32% of *H. amboinensis* and 23% of *H. tubifera* transcripts aligned to proteins in the UniProt database (Fig. 2a). About half of the sequences with hits to UniProt have associated gene ontology assignments. Predicted peptides were similarly annotated by Blastp alignment to the UniProt database with an e-value cutoff of 1 × 10^−5^. Protein domains were identified using HMMER v3.1b1 against the Pfam 28.0 database. Approximately 63% of the predicted peptides in both sponges have matches in UniProt or contain identifiable protein domains but only about 40% are associated with gene ontology annotations (Fig. 2a). The low percentage of annotation for predicted peptides may be due to the scarcity of poriferan sequences in most public data repositories. Alignment to the Ensembl metazoan database reveals that about 75–85% of predicted peptides in the two sponges are similar to sequences from other animals, with the majority of sequences matching to peptides in the sponge, *A. queenslandica* (Fig. 2b).

### Global comparison of sponge genomes

**Figure 3:**
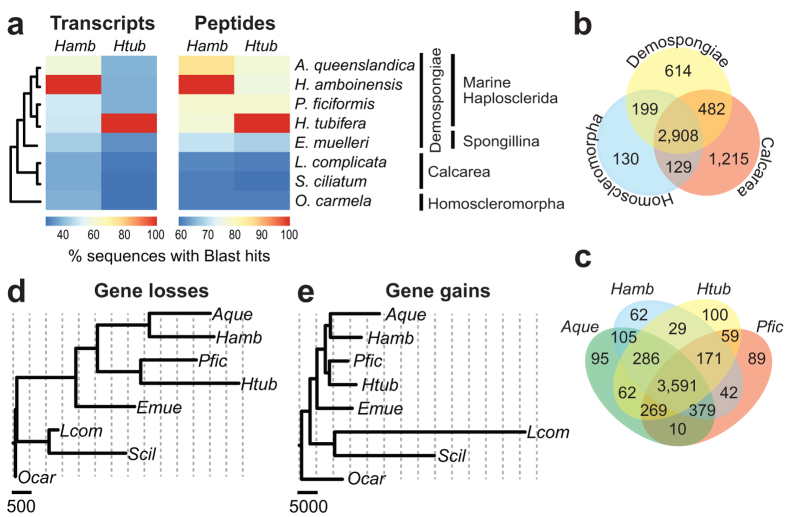
Diversification of sponge genomes. (**a**) Transcript and peptide sequences of *H. amboinensis* and *H. tubifera* were compared to sequences from other sponges. Alignments were conducted using Blastx for transcripts and Blastp for peptides. Heatmaps represent the percent of transcripts (left) or predicted peptides (right) with detectable sequence similarity to other sponges at an e-value threshold of 1 × 10^−5^ (red, high; blue, low). (**b**) Comparison of the number of shared and class-specific orthologous protein groups from OrthoMCL analysis. (**c**) Shared and species-specific orthologous protein groups in four haplosclerid sponges. (**d**) Gene losses in sponge lineages as estimated by EvolMap analysis. The scale bar corresponds to 500 genes. (**e**) Gene gains in sponge lineages as estimated by EvolMap analysis. The scale bar corresponds to 5,000 genes. EvolMap trees are rooted on *O. carmela*. Species names are abbreviated as follows: *A. queenslandica* (*Aque*); *H. amboinensis* (*Hamb*); *H. tubifera* (*Htub*); *P. ficiformis (Pfic)*; *E. muelleri* (*Emue*); *L. complicata* (*Lcom*); *S. ciliatum* (*Scil*); *O. carmela* (*Ocar*).

To determine the similarity of the transcriptomes of *H. amboinensis* and *H. tubifera* to gene sequences in other sponges, we performed global Blast comparisons between all the transcripts or predicted peptides from different sponge species. As expected, pairwise global comparison reveals greater similarity (>50% for transcripts; >80% for peptides) of *Haliclona* sequences to other demosponges (Fig. 3a). In contrast, *Haliclona* sequences have fewer (<40% for transcripts; <65% for peptides) Blast hits to calcareous and homoscleromorph sequences, supporting the substantial divergence between demosponges and the calcareous-homoscleromorph sister group^13^. Within the demosponge group, the marine haplosclerids exhibit greater sequence similarity (>55% for transcripts; >80% for peptides) amongst themselves than to the freshwater demosponge, *Ephydatia muelleri*. 85% of *H. amboinensis* and 75% of *H. tubifera* peptides are similar to genes in the *A. queenslandica* genome. Reciprocally, Blastp alignment reveals that more than 80% of *A. queenslandica* genes possess significant similarity to peptides recovered from the adult transcriptomes of *H. amboinensis and H. tubifera* (Supplementary Table 4). This suggests that despite being derived from a single developmental stage, the adult transcriptomes of *Haliclona* capture many of the genes present in the genome of the model demosponge.

To determine the number of shared and unique protein families among different sponges, we constructed orthologous gene clusters for 8 sponge species representing 3 classes using OrthoMCL with default settings. After all-against-all comparisons of sequences, 264,663 proteins were clustered into 6,996 orthologous groups (Fig. 3b). Of these groups, 2,908 (42%) are common to all eight species. Calcareous sponges possess the highest number (1,215) of unique orthologous clusters, indicating that this sponge lineage may have experienced gene expansions after divergence from the last common ancestor of sponges. Looking just at orthologous clusters present in haplosclerid demosponges, we found 3,591 out of 5,397 (66%) that are common to the four species represented in the analysis (Fig. 3c). Very few orthologous protein clusters are unique to each of the haplosclerid demosponges, suggesting that these sponges possess highly similar sets of genes.

EvolMap analysis was performed to estimate gene gains and losses in the different sponge lineages. This method identifies gene orthologs by all-to-all Blast followed by Needleman-Wunsch alignment to determine similarity scores. The algorithm then traverses the species tree to estimate ancestral gene content at each node and applies Dollo parsimony-based comparison of these ancestral genes to determine how many have been lost or gained within each branch^24^. Peptide sequences from the non-redundant assemblies of *H. amboinensis*, *H. tubifera*, and *P. ficiformis* were used in the analysis to minimize the inflation of gene gain estimates, although this data set may underestimate the true genetic diversity in haplosclerid sponges because the transcriptomes are derived solely from the adult stage. The *O. carmela* genome was used as the outgroup to compare the gene expansions occurring along the demosponge and calcareous lineages. EvolMap analysis reveals that more gene losses and less gains occurred on the branches leading to the demosponges (Fig. 3d-e), suggesting that the ancestor of this sponge class underwent periods of genomic reduction. Marine haplosclerid lineages exhibit a similar extent of gene loss whereas gene gains are slightly higher for *A. queenslandica*, which may be due to inclusion of its full genome in the analysis. In contrast, extensive gains and fewer losses were observed on the branch leading to the calcareous group (Supplementary Table 5), which is also reflected in the high number of unique orthologous gene clusters observed for this class. Because the sequences for *S. ciliatum* and *L. complicata* are derived from genome and non-redundant multi-developmental stage transcriptome data^10^, respectively, the estimated gains are likely to be true gains and not only artifacts of assembly. Nevertheless, while this analysis provides us with an estimate of genomic changes in different sponge lineages, a more accurate picture of the genetic history of diverse sponge groups will emerge with future genome sequencing efforts.

### Genomic innovations for ecological adaptation

Sponges possess a functional metazoan toolkit^4^. Although only about 25% of putative peptides in the four haplosclerid demosponges are associated with gene ontology assignments based on their top Blastp match in the UniProt database (Supplementary Figure 4), metabolic processes, transport, signal transduction, and catalytic activity are the most abundant gene ontology terms. As in other sponges, haplosclerids possess genes that underlie the basic characteristics of multicellular organisms, including control of the cell cycle, cell differentiation, apoptotic processes, extracellular matrix, cell adhesion, and innate immunity^13^,^25^. The most abundant protein domains are known to be involved in metabolism, cell adhesion, cell structural support, signaling, immune response, apoptosis and transcriptional control (Supplementary Figure 5). Below we highlight some gene families that may underlie the unique characteristics of each sponge lineage.

### Transcription factors

**Figure 4:**
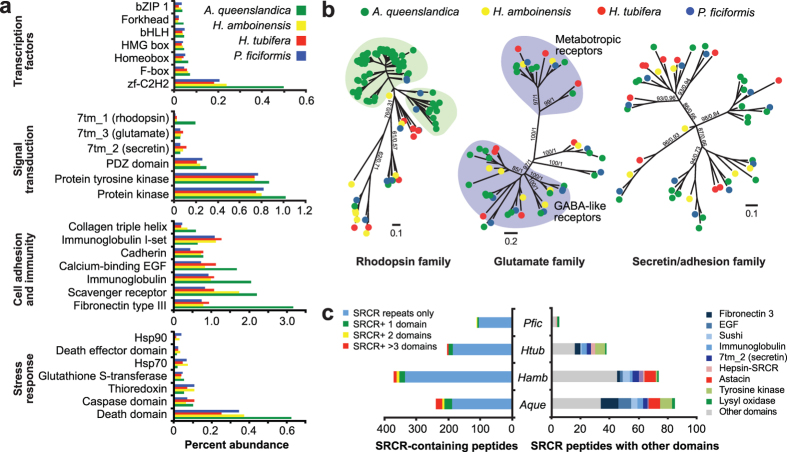
Functional annotation of transcriptomes. (**a**) Abundance of selected Pfam domains involved in transcription, cell adhesion, signal transduction, and stress response in four haplosclerid sponges. Percent abundance is shown relative to the total number of predicted Pfam domains for each species (green, *A. queenslandica*; yellow, *H. amboinensis*; red, *H. tubifera*; blue, *P. ficiformis*). (**b**) Expansion and diversification of families of G-protein coupled receptors (GPCRs) in haplosclerid sponges (green, *A. queenslandica*; yellow, *H. amboinensis*; red, *H. tubifera*; blue, *P. ficiformis*). The unrooted GPCR trees were derived from Bayesian analysis. Numbers on selected branches represent maximum likelihood bootstrap values and Bayesian posterior probabilities, respectively. Green shading represents rhodopsin GPCRs expanded in *A. queenslandica*. Blue shading indicates clusters of glutamate GPCRs. (**c**) Abundance and diversity of scavenger receptor-domain containing peptides in the four haplosclerid species (*Aque, A. queenslandica; Hamb, H. amboinensis; Htub, H. tubifera; Pfic, P. ficiformis*). The total number of SRCR domain-containing peptides in each species is shown on the left while the number of SRCR peptides associated with other protein domains is shown on the right.

Comparison of selected Pfam domains in the four haplosclerid sponges reveals similar abundance patterns for the different transcription factor families, which may have integral roles in controlling development, shaping sponge tissue architecture, and regulating responses to the environment (Fig. 4a). Sponges possess transcription factor families similar to that of metazoans. In *A. queenslandica*, these transcription factors have been found to be expressed at various stages of the sponge lifecycle. bZip, Tbox, bHLH, and homeobox factors are enriched in the competent larvae of *A. queenslandica* and may regulate the genes necessary for settlement, while zinc finger, forkhead, ETS, and homeobox domain-containing factors may control the widespread transcriptional changes observed immediately after settlement as metamorphosis begins^26^. Members of these transcription factor families are also present in the transcriptomes of *H. amboinensis*, *H. tubifera* and *P. ficiformis*. Whether they are expressed in the same manner, or if they regulate the same sets of genes, remains to be discovered.

### Signal transduction

The haplosclerid sponges possess a diverse set of peptides containing domains involved in signal transduction (Fig. 4a). PDZ domains and protein kinases are found to be similarly abundant in all four species. Sponges are also known to possess an expanded and diversified set of rhodopsin, secretin/adhesion, and glutamate G-protein couple receptors (GCPRs)^27^. Most groups of glutamate receptors are represented in the four haplosclerids while rhodopsin-family GPCRs have expanded in *A. queenslandica* (Fig. 4a,b).

GPCRs are membrane proteins that mediate a wide variety of cellular responses to environmental stimulants, such as allelochemicals and inducers of settlement and metamorphosis^28^. Differences in GPCR content and function reflect differences in sponge physiology and behavior. GPCRs expressed in sponge larvae may influence the patterns of settlement that govern sponge assemblage distribution^28^. Some sponge larvae exhibit photosensitive behavior and swim away from light, thus ensuring their settlement in shaded areas under rocks or coral rubble for protection against intense sunlight or UV radiation^29^. This behavior may be regulated by photoreceptive pigments and the expression of specific sets of GPCRs. Other potential functions of the diverse GPCR families in sponges are coordination of cellular contractions, regulation of the uptake of dissolved organic matter from seawater, or even regulation of sponge morphology and tissue properties through the detection of fluid shear stress^30^,^31^,^32^.

### Cell adhesion and structural components

Haplosclerid sponge bodies are composed of proteinaceous mesohyl in which various cells are embedded. Sponges maintain tissue integrity within a colony through strong cell aggregation and allorecognition properties^33^,^34^. These same properties also promote reaggregation and regrowth of damaged or fragmented colonies. Not surprisingly, cell adhesion and extracellular matrix-related domains, such as integrin, immunoglobulin I-set, and cadherin are similarly abundant in the sponges (Fig. 4a). All four haplosclerids possess many collagen, calcium-binding EGF, fibronectin, and immunoglobulin domains, although these are more enriched in the *A. queenslandica* genome. It is important to note, however, that except for *A. queenslandica,* the domain counts for the other sponges are derived from single-stage transcriptome data and may not capture the complete repertoire of genes in these species.

Spongin is a distinguising character of most demosponges, providing support and stability to the sponge tissue. Spongin short-chain collagens are homologous to type IV collagen, one of the main extracellular matrix components ubiquitous in vertebrates and some invertebrates^35^. Not surprisingly, we identified spongin short-chain collagen sequences in the haplosclerid demosponges (Supplementary Figure 5). We further identified sequences for silicatein, an enzyme involved in biosilica formation and spiculogenesis^36^. Although the phylogenetic tree for this gene is not well resolved, the demosponge sequences still cluster together. Similarly, we identified homologs of spherulin, which is a gene acquired from bacteria that has become an integral component of the biomineralization strategy of the coralline demosponge, *Astrosclera willeyana*^37^. Interestingly, we did not find spherulin homologs outside of the demosponge group, suggesting that the horizontal transfer event occurred specifically in this lineage.

In *Haliclona,* intracellular spongin fibres formed by chains of specialized cells function as supplementary skeletal support during the production of the more rigid spicule and spongin skeleton^8^. The loose organization of the sponge body enables morphological plasticity that is responsive to environmental factors^38^. For example, sponges in high water flow environments can induce spongin production and spicule formation to form tougher tissues that can withstand high water flow while still maintaining efficient filter feeding capacity^38^,^39^.

### Immunity

The scavenger receptor cysteine-rich (SRCR) domain is a highly conserved domain found in sponges and in other animals^40^. Scavenger receptors, which are known to associate with diverse co-receptors, are highly versatile and can recognize a large repertoire of ligands. They may function in cell-cell recognition, aggregation, lipid recognition, pattern recognition, phagocytosis, and pathogen clearance and transport^41^. We identified many SRCR-containing peptides in the four haplosclerids (Fig. 4a). *H. amboinensis* has the greatest number of SRCR-containing peptides, although the majority of these are composed of SRCR repeats only. Both *A. queenslandica* and *H. amboinensis* possess more SRCR peptides associated with various other protein domains (Fig. 4c). The presence of this diverse class of receptors in the cell membranes of sponges may be important for the selection or clearance of bacteria from sponge tissues. Moreover, the modified domain architectures suggest that they may also be involved in a wider range of cellular functions.

The microbial abundance in sponge tissues is reflected in the complement of immune-related genes in the host. For instance, *H. tubifera, A. queenslandica,* and *H. amboinensis*, which are low microbial abundance sponges^42^, exhibit expansion and diversification of immune-related genes, particularly scavenger receptors, which may be involved in the selection of specific symbionts and maintenance of a lower bacteria population. On the other hand, *P. ficiformis*, a known high microbial abundance sponge^43^, has a reduced repertoire of scavenger receptors. The core microbiome of sponges effectively extends the genetic potential of the host, providing a pool of novel genes that represent functional innovations that may contribute to the survival of the holobiont under stressful conditions. Rapid shifts in the natural bacterial community due to environmental perturbation can affect the stability of the sponge-bacteria interactions and result in the deterioration of sponge health^44^.

### Stress response

Stress response-related protein domains, such as heat shock protein (Hsp) and thioredoxin, which are involved in protein stabilization and antioxidant defense, respectively, are abundant in *H. amboinensis* and *P. ficiformis* (Fig. 4a). These two sponges also possess many proteins with death effector domains (DED) that regulate cellular homeostasis through the simultaneous control of proliferation and apoptosis^45^. Caspases, which are cysteine proteases that regulate apoptosis, as well as glutathione S-transferase (GST) domains, which function in the antioxidant response, are similarly abundant in all four sponge species. Critical deployment of stress response proteins and antioxidant defenses are important mechanisms that protect the organism and support restoration of cellular homeostasis. Modifications of the complement of stress response proteins found in each sponge species may underlie differences in their tolerance to environmental perturbations.

## Conclusions

Transcriptome sequencing of two haplosclerid demosponges and comparative analysis with other sponge species reveal that haplosclerid sponges possess a streamlined gene complement compared to other sponge classes. This core gene repertoire contains all the tools needed for sponge function and is flexible enough to allow diversification into different habitats. The invention of new functions based on this genetic toolkit may have occurred through exaptation of preexisting genes and rewiring of gene regulatory networks^46^. The streamlining of genomes after bursts of molecular invention associated with species radiation is a common theme in organismal evolution^47^ and is also likely to have contributed to the diversification of haplosclerid demosponges.

Haplosclerid demosponges are found in various habitats but are common in shallow water environments that are characterized by high water flow, variable temperatures, intense light exposure, fluxes in salinity, as well as input of nutrients from coastal ecosystems. To survive in these highly dynamic environments, sponges must possess mechanisms to maintain tissue integrity and repair tissue damage. While haplosclerid sponges share a highly similar set of genes, key differences in specific gene families, such as GPCRs, immune response-related genes, and stress response genes, provide clues to the unique adaptations of each species.

Thus, sponges, and demosponges in particular, provide a glimpse into how different molecular innovations emerging from an essentially similar core gene complement allow adaptation to variable conditions encountered within a habitat range. In future studies, it would be of interest to look at the sponge holobiont as a whole and determine how functional genes of the sponge-associated microbiota contribute to local adaptation of their host. Further genome and metagenome-level comparisons with other sponge species will provide a deeper understanding of the genomic features that support the ecological success of this diverse group of organisms.

## Methods

### Sample collection

Sponges were collected by SCUBA diving in Malilnep Channel, Bolinao, Pangasinan (*H. amboinensis* - 16.43968°N 119.94434°E; *H. tubifera* - 16.43530°N 119.94062°E) in September, 2013 at a depth of 7–10 meters for *H. amboinensis* and 1–2 meters for *H. tubifera.* Collections were conducted with permission from the Philippines Department of Agriculture Bureau of Fisheries and Aquatic Resources (DA-BFAR GP-0075-14). Tissues were dissected and cleaned of macroscopic contaminants before storage in RNAlater (Ambion). Samples were transferred to liquid nitrogen for transport and stored at −80 °C for subsequent molecular analyses.

### Sponge characterization and identification

Sponge characterization was done by observing the morphological features of collected sponges (*in situ* and voucher specimens), including growth form, texture, spicule forms and architecture. Fresh sponge samples were fixed in 95% ethanol solution and used for spicule observation and DNA extraction. Spicules were prepared following the bleach digestion protocol^20^. Briefly, a longitudinal section of the sponge was dissolved in a household bleach (5% sodium hypochlorite) and the remaining mineral skeleton was viewed by optical microscopy (Zeiss Primo Star and Nikon E200). Hand-sectioning was also done to examine the skeleton structure of the sponge. Features were matched to descriptions in Systema Porifera^20^ and The Sponge Guide^48^ to confirm sponge identity.

### Genomic DNA extraction and COI amplification

Genomic DNA was extracted from sponge tissues using the xanthogenate DNA isolation method^49^. The 5′ region of cytochrome oxidase I (COI) gene was amplified using 0.5 uM of the degenerate primers dgLCO1490 and dgHCO2198^50^ in a reaction mix containing buffer (20 mM Tris-HCl pH 8.4, 50 mM KCl), 1.5 mM MgCl_2_, 0.2 uM dNTPs, and 0.25 units of Taq DNA polymerase (Invitrogen). Amplification was performed using a standard three-step PCR with initial denaturation of 3 minutes at 94 °C followed by 40 cycles of 30 seconds at 94 °C, 30 seconds at 40 °C and 1 minute at 72 °C, and a final extension step of 5 minutes at 72 °C. PCR amplicons were purified and sequenced (1st Base Laboratories, Malaysia).

### RNA extraction, quantity and quality assessment

RNA was extracted using Trizol Reagent (Invitrogen) following the manufacturer’s protocol. Longitudinal sections of sponge tissues were manually homogenized to minimize RNA shearing. Contaminating genomic DNA was removed using the DNAfree kit (Ambion). Nucleic acid concentrations were obtained using a BioSpec Nanodrop spectrophotometer (Shimadzu). The integrity of RNA samples was determined by electrophoresis on a native agarose gel with denaturing loading dye. RNA quality was further assessed using the mRNA Pico Series II assay on the Agilent Bioanalyzer 2100 System (Agilent Technologies).

### RNA sequencing, data filtering, and *de novo* assembly

Genetic material from three independent sponge colonies were used in the assembly of a reference transcriptome for each species. Barcoded cDNA libraries derived from non-reproductive adult sponge tissues were prepared using the Illumina TruSeq RNA Sample Prep Kit protocol. The rRNA-depleted and mRNA-enriched libraries were sequenced on the Illumina HiSeq 2000 platform with 100 bp paired-end reads (Beijing Genomic Institute, Hong Kong). Raw sequence reads were filtered to remove adapter sequences and low-quality reads. Read quality was visualized using FastQC 0.10.1 (Babraham Bioinformatics). Trimmomatic 0.32^51^ was used to trim the first 15 bases of the reads and bases with a quality score below 30 at leading and trailing ends. Reads were then scanned with a 4-base sliding window, cutting when the average quality per base dropped below 30. Only reads that passed the quality filters and were longer than 36 bases were retained for further analysis. *De novo* transcriptome assemblies were carried out on Trinity (trinityrnaseq_r2013-11-10)^52^. To evaluate the quality of transcriptome assemblies, all clean paired-end reads used for assembly were mapped back to the assembled transcripts using the alignReads.pl script incorporated in the Trinity package.

### Filtering of transcriptome assemblies and peptide prediction

Reads were first mapped back to the reference transcriptome assembly using RSEM^53^. Isoforms with the highest combined isoform percentage (IsoPct) from three libraries, or the longest transcript for isoforms with the same IsoPct, were retained. Isoforms with zero IsoPct in all libraries were removed, as these may be misassembled transcripts. CD-HIT-EST^54^ was then implemented using default parameters to cluster similar sequences and filter out redundant transcripts. The non-redundant reference transcript set was used for all further analyses. Coding regions were identified using the TransDecoder script included in the Trinity package. Only the longest ORF from each transcript was included in building the reference peptide set for each sponge.

### Annotation of transcripts and peptides

Transcripts and peptide sequences for the longest open reading frames (ORFs) were mapped against UniProtKB/Swiss-Prot database (February 2015) and to predicted peptides of *A. queenslandica*^4^, *E. muelleri, S. ciliatum* and *O. carmela* genomes, as well as *P. ficiformis* and *L. complicata* transcriptomes. Sponge sequences were downloaded from Compagen^55^, except for *A. queenslandica* sequences, which were downloaded from Ensembl Metazoa, and *P. ficiformis* sequences, which were shared by Ana Riesgo (Natural History Museum, London). Only sponge peptides greater than 100 amino acids in length were included in all subsequent analyses. The top Blast hit for each sequence was used as input into Blast2GO^56^ to retrieve gene ontology terms. Protein domains were identified by mapping predicted peptides against the Pfam 28.0^57^ database using HMMER v3.1b1^58^. Gene homologs were identified by reciprocal Blastp alignments with an e-value cutoff of 1 × 10^−5^ .

### Analysis of sequence similarity, orthologous groups and gene histories

Pairwise sequence comparisons between different sponge species were performed using Blastx and Blastp alignments at an e-value cutoff of 1 × 10^−5^. The percent of transcripts or predicted peptides with detectable sequence similarity to various sponges was visualized as a heatmap using the pheatmap R package. Orthologous gene groups were identified using OrthoMCL^59^ with default settings (e-value 1 × 10^−5^, protein identity 50%, and MCL inflation of 1.5). EvolMap^24^ was used to infer ancestral genome content and trace the history of gene gains and losses in the different sponge species. All-to-all Blast was run to retrieve the 300 top-scoring gene pairs for which normalized similarity scores were generated. Orthologous genes were identified as symmetrical best alignments scoring above the minimum threshold set at 250.

### Phylogenetic analysis

Sequences were aligned using ClustalW^60^ and trimmed with Gblocks^61^. The best-fit substitution model for each alignment was determined using jmodeltest (v2.1.7) for nucleotides and prottest (v3.4) for amino acids. Maximum-likelihood analysis was implemented on PhyML 3.0^62^ with 1,000 bootstrap replicates. Bayesian inference analysis was executed on MrBayes 3.2.2^63^ using two independent MCMC runs with four chains per run. Each analysis set for 1 million generations sampled every 100 trees or until the standard deviation of split frequencies was <0.01. The first 25% of trees were discarded as burn-in. COI sequences of other sponges were downloaded from NCBI (Supplementary Table 6). Specific genes and gene families were identified through reciprocal Blast. Other peptide sequences used for phylogenetic comparisons were downloaded from NCBI.

## Additional Information

**How to cite this article**: Guzman, C. and Conaco, C. Comparative transcriptome analysis reveals insights into the streamlined genomes of haplosclerid demosponges. *Sci. Rep.* **6**, 18774; doi: 10.1038/srep18774 (2016).

## Supplementary Material

Supplementary Information

## References

[b1] Bell J. J. The functional roles of marine sponges. Estuarine, coastal and shelf science 79, 341–353 (2008).

[b2] de Goeij J. M. *et al.* Surviving in a Marine Desert: The Sponge Loop Retains Resources Within Coral Reefs. Science (New York, N.Y.) 342, 108–110, doi: 10.1126/science.1241981 (2013). 24092742

[b3] Wisshak M., Schönberg C., Form A. & Freiwald A. Ocean Acidification Accelerates Reef Bioerosion. PloS one 7, e45124 (2012). 23028797 10.1371/journal.pone.0045124 PMC3445580

[b4] Srivastava M. *et al.* The Amphimedon queenslandica genome and the evolution of animal complexity. Nature 466, 720–726, doi: 10.1038/nature09201 (2010). 20686567 PMC3130542

[b5] Li C. W., Chen J. Y. & Hua T. E. Precambrian sponges with cellular structures. Science (New York, N.Y.) 279, 879–882 (1998). 10.1126/science.279.5352.879 9452391

[b6] Yin Z. *et al.* Sponge grade body fossil with cellular resolution dating 60 Myr before the Cambrian. Proceedings of the National Academy of Sciences of the United States of America, doi: 10.1073/pnas.1414577112 (2015). PMC4378401 25775601

[b7] Harrison F. & De Vos L. In Microscopic Anatomy of Invertebrates Vol. 2 (eds Frederick W. Harrison & Edward E. Ruppert) (Wiley-Liss, 1990).

[b8] Bergquist P. R. Sponges. (University of California Press, 1978).

[b9] Taylor M. W., Radax R., Steger D. & Wagner M. Sponge-associated microorganisms: evolution, ecology, and biotechnological potential. Microbiology and molecular biology reviews 71, 295–347, doi: 10.1128/mmbr.00040-06 (2007). 17554047 PMC1899876

[b10] Fortunato S. A. *et al.* Calcisponges have a ParaHox gene and dynamic expression of dispersed NK homeobox genes. Nature 514, 620–623, doi: 10.1038/nature13881 (2014). 25355364

[b11] Nichols S. A., Dirks W., Pearse J. S. & King N. Early evolution of animal cell signaling and adhesion genes. Proceedings of the National Academy of Sciences of the United States of America 103, 12451–12456, doi: 10.1073/pnas.0604065103 (2006). 16891419 PMC1567900

[b12] Harcet M. *et al.* Demosponge EST sequencing reveals a complex genetic toolkit of the simplest metazoans. Molecular biology and evolution 27, 2747–2756, doi: 10.1093/molbev/msq174 (2010). 20621960 PMC2981516

[b13] Riesgo A., Farrar N., Windsor P. J., Giribet G. & Leys S. P. The analysis of eight transcriptomes from all poriferan classes reveals surprising genetic complexity in sponges. Molecular biology and evolution 31, 1102–1120, doi: 10.1093/molbev/msu057 (2014). 24497032

[b14] Van Soest R. W. *et al.* Global diversity of sponges (Porifera). PloS one 7, e35105, doi: 10.1371/journal.pone.0035105 (2012). 22558119 PMC3338747

[b15] Palumbi S. R. How Body Plans Limit Acclimation: Responses of a Demosponge to Wave Force. Ecology 67, 208–214 (1986).

[b16] Bell J. J., Davy S. K., Jones T., Taylor M. W. & Webster N. S. Could some coral reefs become sponge reefs as our climate changes? Global change biology 19, 2613–2624 (2013). 23553821 10.1111/gcb.12212

[b17] Cardenas P., Perez T. & Boury-Esnault N. Sponge systematics facing new challenges. Advances in marine biology 61, 79–209, doi: 10.1016/b978-0-12-387787-1.00010-6 (2012). 22560778

[b18] Longakit M. B. A., Sotto F. B. & Kelly M. The shallow water marine sponges (Porifera) of Cebu, Philippines. Science Diliman 17, 52–74 (2005).

[b19] Kang D. W., Lee K. J. & Sim C. J. Two New Marine Sponges of the Genus Haliclona (Haplosclerida: Chalinidae) from Korea. Animal systematics, evolution and diversity 29, 51–55 (2013).

[b20] Hooper J. N. A. & Van Soest R. W. M. In Systema Porifera (eds John, N. A., Hooper, Rob, W. M. Van Soest & Philippe, Willenz) Ch. 1, 1–7 (Springer: US, , 2002).

[b21] Redmond, N. E. *et al.* Phylogeny and systematics of demospongiae in light of new small-subunit ribosomal DNA (18S) sequences. Integrative and comparative biology 53, 388–415, doi: 10.1093/icb/ict078 (2013). 23793549

[b22] Hill M. S. *et al.* Reconstruction of family-level phylogenetic relationships within Demospongiae (Porifera) using nuclear encoded housekeeping genes. PloS one 8, e50437, doi: 10.1371/journal.pone.0050437 (2013). 23372644 PMC3553142

[b23] Erpenbeck D., Hooper J. N. A. & Worheide G. CO1 phylogenies in diploblasts and the ‘Barcoding of Life’— are we sequencing a suboptimal partition? Molecular ecology notes 6, 550–553, doi: 10.1111/j.1471-8286.2005.01259.x (2006).

[b24] Sakarya O., Kosik K. S. & Oakley T. H. Reconstructing ancestral genome content based on symmetrical best alignments and Dollo parsimony. Bioinformatics (Oxford, England) 24, 606–612, doi: 10.1093/bioinformatics/btn005 (2008). 18184685

[b25] Perez-Porro A. R., Navarro-Gomez D., Uriz M. J. & Giribet G. A NGS approach to the encrusting Mediterranean sponge Crella elegans (Porifera, Demospongiae, Poecilosclerida): transcriptome sequencing, characterization and overview of the gene expression along three life cycle stages. Molecular ecology resources 13, 494–509, doi: 10.1111/1755-0998.12085 (2013). 23437888

[b26] Conaco C. *et al.* Transcriptome profiling of the demosponge Amphimedon queenslandica reveals genome-wide events that accompany major life cycle transitions. BMC genomics 13, 209, doi: 10.1186/1471-2164-13-209 (2012). 22646746 PMC3447736

[b27] Krishnan A. *et al.* The GPCR repertoire in the demosponge Amphimedon queenslandica : insights into the GPCR system at the early divergence of animals. BMC evolutionary biology 14, 270, doi: 10.1186/s12862-014-0270-4 (2014). 25528161 PMC4302439

[b28] Degnan S. M. & Degnan B. M. The initiation of metamorphosis as an ancient polyphenic trait and its role in metazoan life-cycle evolution. Philosophical transactions of the Royal Society of London. Series B, Biological sciences 365, 641–651, doi: 10.1098/rstb.2009.0248 (2010). 20083639 PMC2817140

[b29] Leys S. P. & Degnan B. M. Cytological basis of photoresponsive behavior in a sponge larva. The Biological bulletin 201, 323–338 (2001). 11751245 10.2307/1543611

[b30] Chachisvilis M., Zhang Y. L. & Frangos J. A. G protein-coupled receptors sense fluid shear stress in endothelial cells. Proceedings of the National Academy of Sciences of the United States of America 103, 15463–15468, doi: 10.1073/pnas.0607224103 (2006). 17030791 PMC1622845

[b31] Ramoino P. *et al.* Metabotropic gamma-aminobutyric acid (GABAB) receptors modulate feeding behavior in the calcisponge Leucandra aspera. Journal of experimental zoology. Part A, Ecological genetics and physiology 315, 132–140, doi: 10.1002/jez.657 (2011). 21370481

[b32] Elliott G. R. & Leys S. P. Coordinated contractions effectively expel water from the aquiferous system of a freshwater sponge. The Journal of experimental biology 210, 3736–3748, doi: 10.1242/jeb.003392 (2007). 17951414

[b33] Gauthier M. & Degnan B. M. Partitioning of genetically distinct cell populations in chimeric juveniles of the sponge Amphimedon queenslandica. Developmental and comparative immunology 32, 1270–1280, doi: 10.1016/j.dci.2008.04.002 (2008). 18514309

[b34] Blumbach B. *et al.* The putative sponge aggregation receptor. Isolation and characterization of a molecule composed of scavenger receptor cysteine-rich domains and short consensus repeats. Journal of cell science 111 (Pt 17), 2635–2644 (1998). 9701562 10.1242/jcs.111.17.2635

[b35] Aouacheria A. *et al.* Insights into early extracellular matrix evolution: spongin short chain collagen-related proteins are homologous to basement membrane type IV collagens and form a novel family widely distributed in invertebrates. Molecular biology and evolution 23, 2288–2302, doi: 10.1093/molbev/msl100 (2006). 16945979

[b36] Shimizu K., Cha J., Stucky G. D. & Morse D. E. Silicatein alpha: cathepsin L-like protein in sponge biosilica. Proceedings of the National Academy of Sciences of the United States of America 95, 6234–6238 (1998). 9600948 10.1073/pnas.95.11.6234 PMC27641

[b37] Jackson D. J., Macis L., Reitner J. & Worheide G. A horizontal gene transfer supported the evolution of an early metazoan biomineralization strategy. BMC evolutionary biology 11, 238, doi: 10.1186/1471-2148-11-238 (2011). 21838889 PMC3163562

[b38] Meroz-Fine E., Shefer S. & Ilan M. Changes in morphology and physiology of an East Mediterranean sponge in different habitats. Marine biology 147, 243–250, doi: 10.1007/s00227-004-1532-2 (2005).

[b39] Palumbi S. R. Tactics of acclimation: morphological changes of sponges in an unpredictable environment. Science (New York, N.Y.) 225, 1478–1480, doi: 10.1126/science.225.4669.1478 (1984). 17770077

[b40] Pancer Z., Munkner J., Muller I. & Muller W. E. A novel member of an ancient superfamily: sponge (Geodia cydonium, Porifera) putative protein that features scavenger receptor cysteine-rich repeats. Gene 193, 211–218 (1997). 9256079 10.1016/s0378-1119(97)00135-2

[b41] Sarrias M. R. *et al.* The Scavenger Receptor Cysteine-Rich (SRCR) domain: an ancient and highly conserved protein module of the innate immune system. Critical reviews in immunology 24, 1–37 (2004). 14995912 10.1615/critrevimmunol.v24.i1.10

[b42] Easson C. G. & Thacker R. W. Phylogenetic signal in the community structure of host-specific microbiomes of tropical marine sponges. Frontiers in microbiology 5, doi: 10.3389/fmicb.2014.00532 (2014). PMC4201110 25368606

[b43] Vacelet J. & Donadey C. Electron microscope study of the association between some sponges and bacteria. Journal of Experimental Marine Biology and Ecology 30, 301–314 (1977).

[b44] Simister R. *et al.* Thermal stress responses in the bacterial biosphere of the Great Barrier Reef sponge, Rhopaloeides odorabile. Environmental microbiology 14, 3232–3246, doi: 10.1111/1462-2920.12010 (2012). 23106937

[b45] Tibbetts M. D., Zheng L. & Lenardo M. J. The death effector domain protein family: regulators of cellular homeostasis. Nature immunology 4, 404–409, doi: 10.1038/ni0503-404 (2003). 12719729

[b46] Miyata T. & Suga H. Divergence pattern of animal gene families and relationship with the Cambrian explosion. BioEssays: news and reviews in molecular, cellular and developmental biology 23, 1018–1027, doi: 10.1002/bies.1147 (2001). 11746218

[b47] Wolf Y. I. & Koonin E. V. Genome reduction as the dominant mode of evolution. BioEssays: news and reviews in molecular, cellular and developmental biology 35, 829–837, doi: 10.1002/bies.201300037 (2013). PMC3840695 23801028

[b48] Zea S., Henkel T. & Pawlik J. *The Sponge Guide: a picture guide to Caribbean sponges.* <Available online at http://www.spongeguide.org> (2009) (Date of access:10/07/2013).

[b49] Jhingan A. K. A novel technology for DNA isolation. Methods molecular cell biology 3, 15–22 (1992).

[b50] Folmer O., Black M., Hoeh W., Lutz R. & Vrijenhoek R. DNA primers for amplification of mitochondrial cytochrome c oxidase subunit I from diverse metazoan invertebrates. Molecular marine biology and biotechnology 3, 294–299 (1994). 7881515

[b51] Bolger A. M., Lohse M. & Usadel B. Trimmomatic: a flexible trimmer for Illumina sequence data. Bioinformatics (Oxford, England) 30, 2114–2120, doi: 10.1093/bioinformatics/btu170 (2014). PMC4103590 24695404

[b52] Grabherr M. G. *et al.* Full-length transcriptome assembly from RNA-Seq data without a reference genome. Nature biotechnology 29, 644–652, doi: 10.1038/nbt.1883 (2011). PMC3571712 21572440

[b53] Li B. & Dewey C. N. RSEM: accurate transcript quantification from RNA-Seq data with or without a reference genome. BMC bioinformatics 12, 323, doi: 10.1186/1471-2105-12-323 (2011). 21816040 PMC3163565

[b54] Li W. & Godzik A. Cd-hit: a fast program for clustering and comparing large sets of protein or nucleotide sequences. Bioinformatics (Oxford, England) 22, 1658–1659, doi: 10.1093/bioinformatics/btl158 (2006). 16731699

[b55] Hemmrich G. & Bosch T. C. Compagen, a comparative genomics platform for early branching metazoan animals, reveals early origins of genes regulating stem-cell differentiation. BioEssays: news and reviews in molecular, cellular and developmental biology 30, 1010–1018, doi: 10.1002/bies.20813 (2008). 18800383

[b56] Conesa A. *et al.* Blast2GO: a universal tool for annotation, visualization and analysis in functional genomics research. Bioinformatics (Oxford, England) 21, 3674–3676, doi: 10.1093/bioinformatics/bti610 (2005). 16081474

[b57] Finn R. D. *et al.* Pfam: the protein families database. Nucleic acids research 42, D222–230, doi: 10.1093/nar/gkt1223 (2014). 24288371 PMC3965110

[b58] Eddy S. R. Profile hidden Markov models. Bioinformatics (Oxford, England) 14, 755–763 (1998). 10.1093/bioinformatics/14.9.755 9918945

[b59] Li L., Stoeckert C. J. Jr. & Roos D. S. OrthoMCL: identification of ortholog groups for eukaryotic genomes. Genome research 13, 2178–2189, doi: 10.1101/gr.1224503 (2003). 12952885 PMC403725

[b60] Thompson J. D., Higgins D. G. & Gibson T. J. CLUSTAL W: improving the sensitivity of progressive multiple sequence alignment through sequence weighting, position-specific gap penalties and weight matrix choice. Nucleic acids research 22, 4673–4680 (1994). 7984417 10.1093/nar/22.22.4673 PMC308517

[b61] Castresana J. Selection of conserved blocks from multiple alignments for their use in phylogenetic analysis. Molecular biology and evolution 17, 540–552 (2000). 10742046 10.1093/oxfordjournals.molbev.a026334

[b62] Guindon S. *et al.* New algorithms and methods to estimate maximum-likelihood phylogenies: assessing the performance of PhyML 3.0. Systematic biology 59, 307–321, doi: 10.1093/sysbio/syq010 (2010). 20525638

[b63] Ronquist F. *et al.* MrBayes 3.2: efficient Bayesian phylogenetic inference and model choice across a large model space. Systematic biology 61, 539–542, doi: 10.1093/sysbio/sys029 (2012). 22357727 PMC3329765

