# Supplementary material for: Comparative transcriptome analysis reveals insights into the streamlined genomes of haplosclerid demosponges
Source: Sci Rep. 2016 Jan 7;6:18774. doi: 10.1038/srep18774 (PMC4704026; doi:10.1038/srep18774)
Supplement: Supplementary Information [file srep18774-s1.pdf]

Supplementary Information

Comparative transcriptome analysis reveals insights into the streamlined genomes of haplosclerid demosponges

Christine Guzman, Cecilia Conaco

Marine Science Institute, College of Science, University of the Philippines, Diliman, Quezon City, Philippines 1101

List of Supplementary Information

Supplementary Figure 1. *Haliclona* species found native to Bolinao, Pangasinan, Philippines.
Supplementary Figure 2. Phylogenetic analyses of membrane-associated guanylate kinase (MAGUK) homologs.
Supplementary Figure 3. Length distribution of assembled transcripts and predicted peptides
Supplementary Figure 4. Abundance of peptides associated with functions related to general cellular processes, metabolic processes, and metazoa-associated processes.
Supplementary Figure 5. Abundance of peptides containing selected protein family domains in the four haplosclerid sponges.
Supplementary Figure 6. Phylogenetic analyses of sponge structural proteins.
Supplementary Table 1. Collection details and morphological characteristics of *H. amboinensis* and *H. tubifera.*
Supplementary Table 2. Reference transcriptome assembly statistics.
Supplementary Table 3. Assembly annotation statistics.
Supplementary Table 4. Percent of *A. queenslandica* proteins with sequence similarity to predicted peptides in other sponge species.
Supplementary Table 5. Estimated number of gene losses and gains based on EvolMap analysis of 8 sponge species.
Supplementary Table 6. Accession numbers of COI sequences used for phylogenetic analysis.
Supplementary Note. Nucleotide and amino acid sequences used for phylogenetic analyses.
References

Supplementary Figures

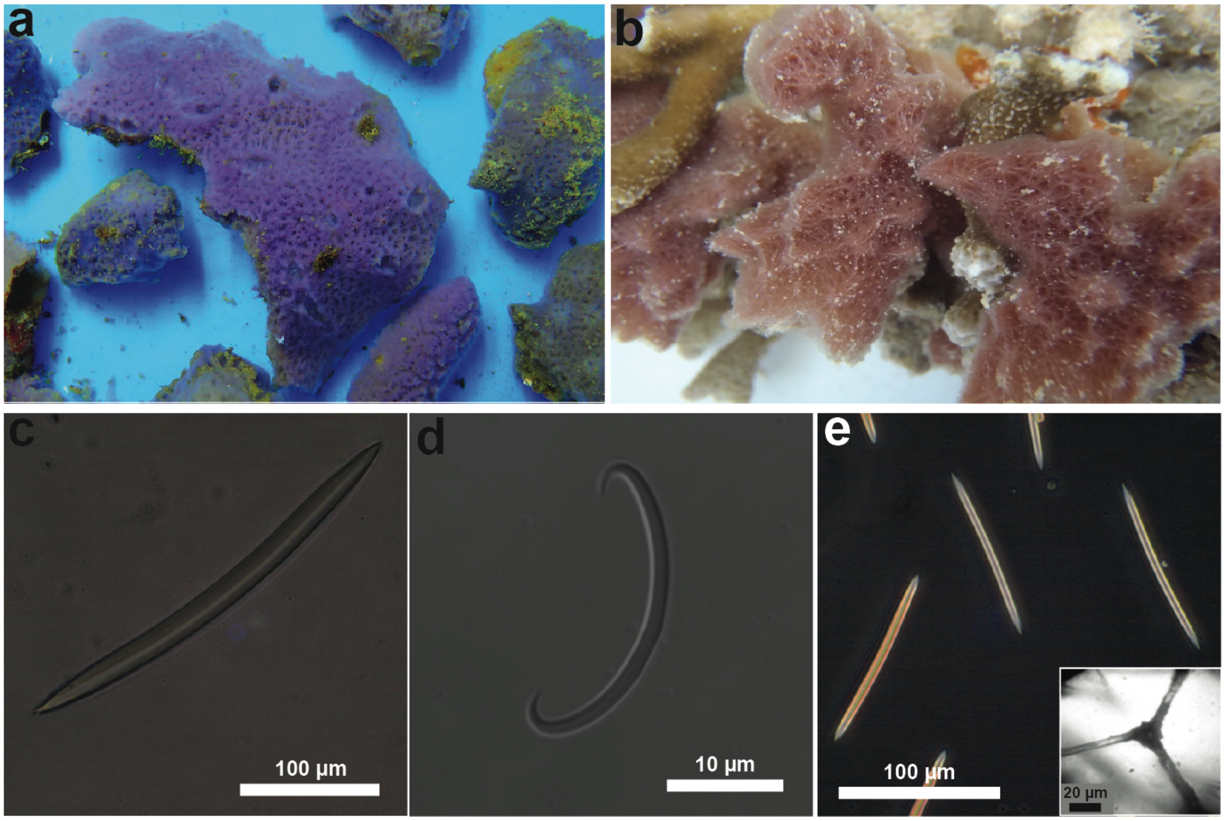

Supplementary Figure 1. *Haliclona* species found native to Bolinao, Pangasinan, Philippines, (a) *H. amboinensis* and (b) *H. tubifera*. The *H. amboinensis* skeleton is composed of (c) megascleres (straight/slightly-curved oxeas) and (d) microscleres (c-shaped sigmas). *H. tubifera* has monosized oxeas (e) forming isodictyal reticulation with spongin visible at junctions between tips of three spicules (inset).

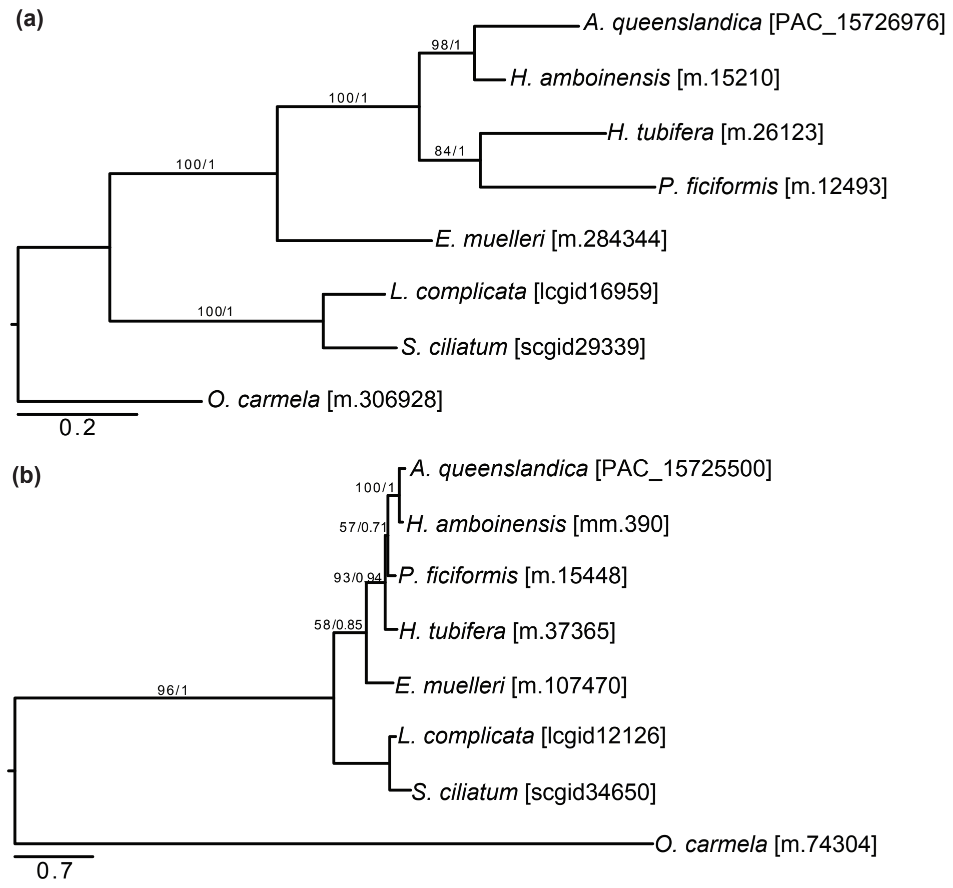

Supplementary Figure 2. Phylogenetic analyses of membrane-associated guanylate kinase (MAGUK) homologs. (a) Disks large (DLG) and (b) membrane-associated guanylate kinase inverted (MAGI) sequences for *H. amboinensis* show closer phylogenetic relationship to sequences in *A. queenslandica* while *H. tubifera* sequences are more similar to *P. ficiformis*. The DLG and MAGI trees rooted on *O. carmela* were calculated using PhyML with the best-fit substitution models WAG+I+G and JTT+I+G, respectively. The tree topology of the Bayesian trees calculated with MrBayes, using the same models, are similar. Numbers at the nodes are bootstrap values for maximum-likelihood and Bayesian posterior probabilities, respectively. Gene sequences for other sponges were obtained from NCBI or by reciprocal Blast against transcriptome datasets in Compagen (Hemmrich & Bosch, 2008).

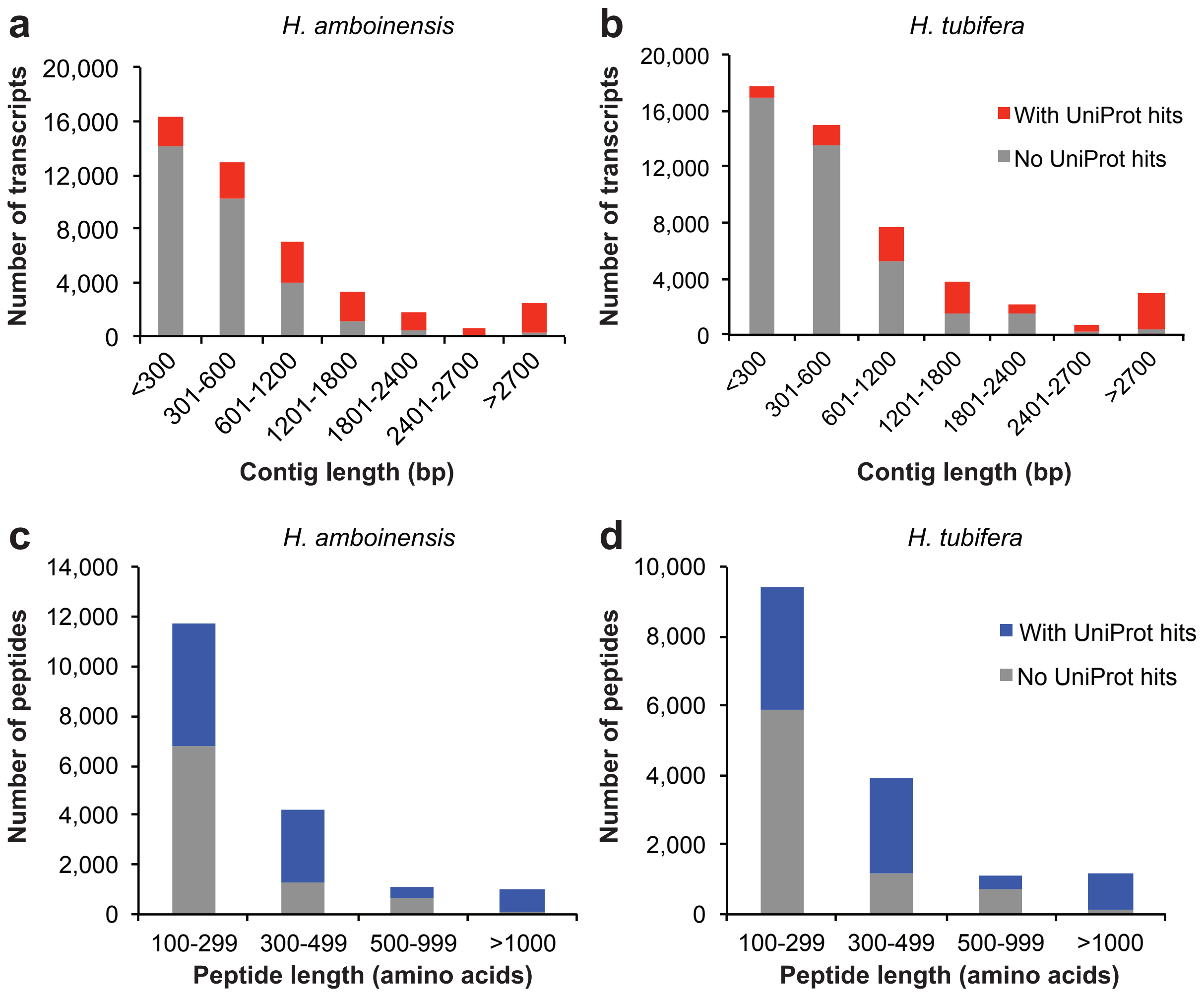

Supplementary Figure 3. Length distribution of assembled transcripts and predicted peptides for *H. amboinensis* (a, c) and *H. tubifera* (b, d). The percent of sequences within each size range with Blast hits in the UniProt database are shown.

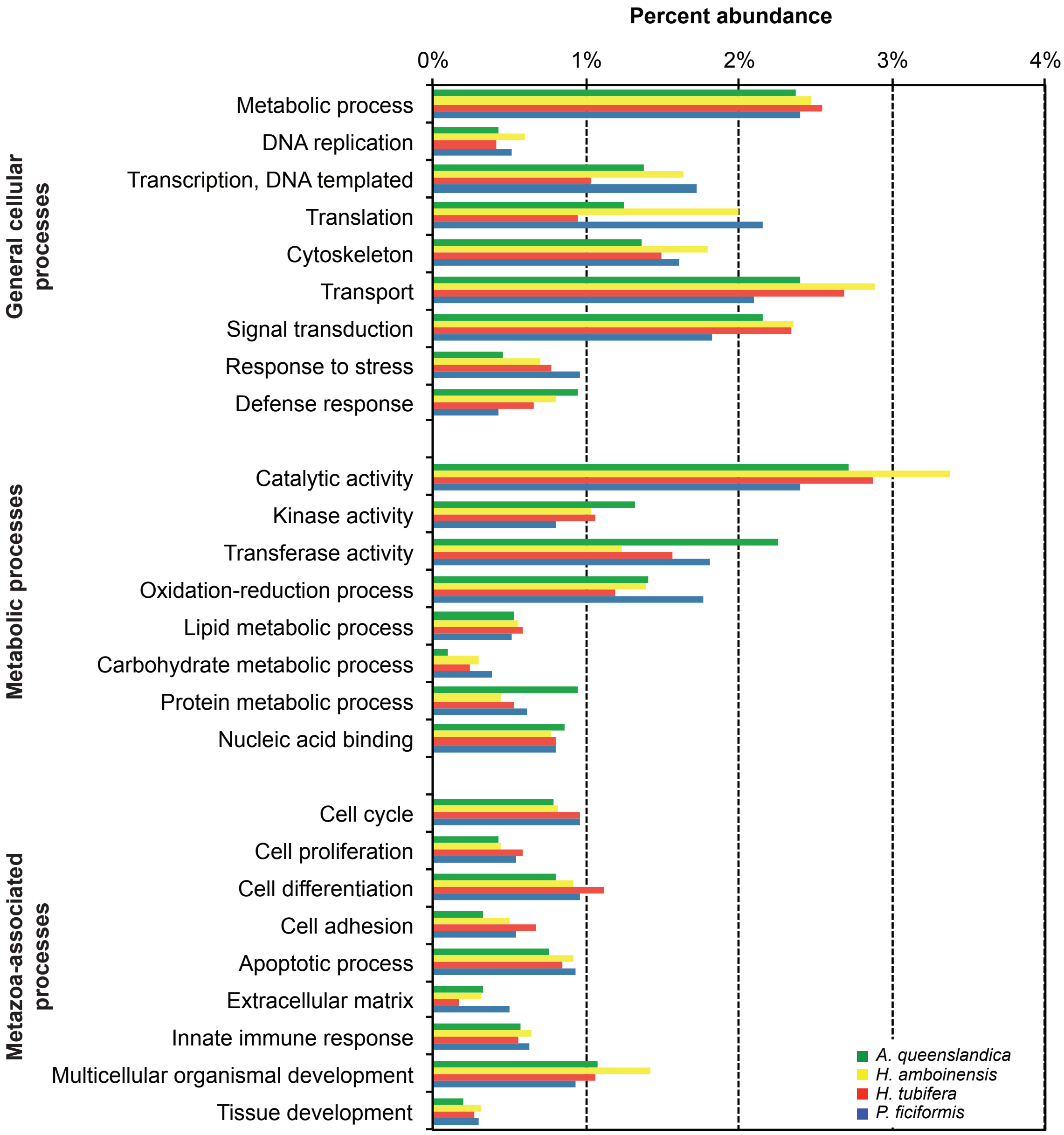

Supplementary Figure 4. Abundance of peptides associated with functions related to general cellular processes, metabolic processes, and metazoa-associated processes. Percent abundance is shown relative to the number of peptides under each gene ontology (GO) term. GO annotations are based on terms associated with the top Blastp hit against the UniProt database.

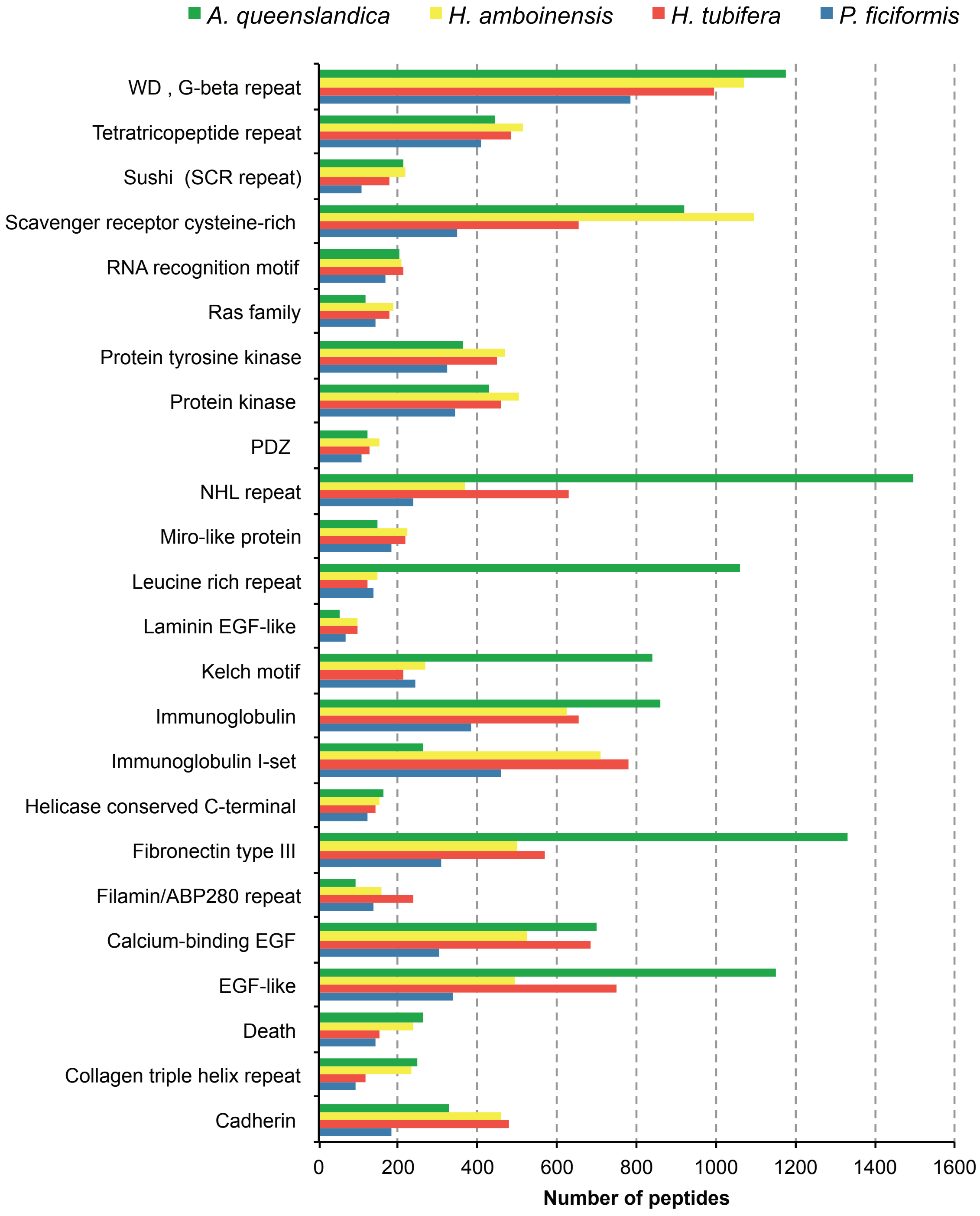

Supplementary Figure 5. Abundance of peptides containing selected protein family domains in the four haplosclerid sponges. Protein domains were identified by mapping predicted peptides to the Pfam 28.0 (Finn et al., 2014) database using HMMER v3.1b1 (Eddy, 1998).

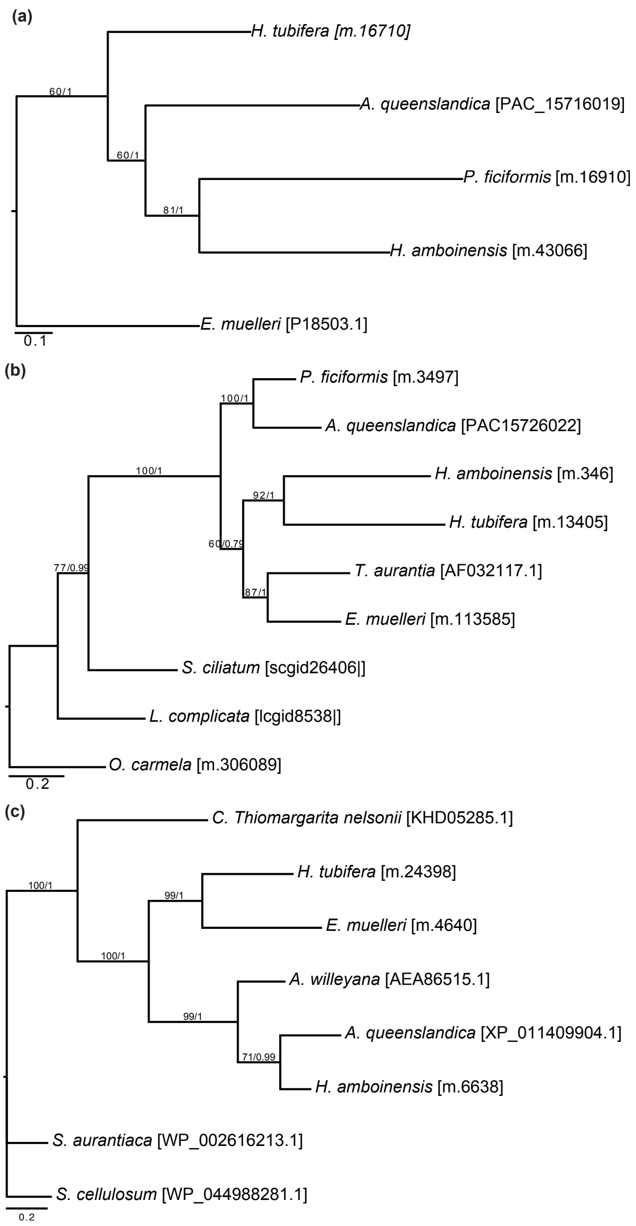

Supplementary Figure 6. Phylogenetic analyses of sponge structural proteins. Tree topologies shown were determined using maximum likelihood analysis on PhyML using the best-fit substitution model WAG+F for (a) spongin short-chain collagen and WAG+G+I for (b) silicatein and (c) spherulin. The spongin short-chain collagen tree is rooted on the freshwater sponge *E. muelleri,* the silicatein tree on the homoscleromorph *O. carmela,* and the spherulin tree on prokaryotes. MrBayes analysis using the same substitution models produced trees with similar topology. Numbers at the nodes are maximum-likelihood bootstrap values and Bayesian posterior probabilities, respectively. Gene sequences for other sponges were obtained from NCBI or by reciprocal Blast against transcriptome datasets in Compagen (Hemmrich & Bosch, 2008).

Supplementary Tables

Supplementary Table 1. Collection details and morphological characteristics of *H. amboinensis* and *H. tubifera.*

	Haliclona tubifera	*Haliclona amboinensis*
**Collection details**		
Date of collection	June and September, 2013	
Locality	Malilnep Channel, Bolinao, Pangasinan, Philippines	
GPS Coordinates	N 16.43530, E 119.94062	N 16.43968, E 119.94434
Depth	1-2 meters	7-10 meters
**Morphological characteristics**		
Color	Dark pink	Pale violet to blue
Growth form	Tubular, oscula at the end of the tube (~0.5-1cm diameter)	Oval to irregularly-shaped;<br>No visible ostia; slightly raised oscula (~0.2cm)
Consistency	Very soft; compressible; quite fragile	Slightly compressible outside/ slightly encrusting inside
Surface	Distinctly porous	Slightly hispid / wrinkled
Exudate	None	None
Skeletal components	Megascleres: oxeas (smooth to curved rods with both ends pointed)<br><br>Spicules are cemented by spongin at the tips	Megascleres: oxeas (straight or curved at the center)<br><br>Microscleres: c-shaped sigmas<br><br>Isotropic reticulation is formed by 6-10 oxeas

Supplementary Table 2. Reference transcriptome assembly statistics.

	H. amboinensis	*H. tubifera*
Number of libraries used to build reference	3	3
Number of reads assembled	140,957,948	161,040,686
Number of transcripts	107,470	124,476
Number of reads aligned back to reference (millions)	122.07 (86.60%)	138.86 (86.23%)
Number of transcripts after isoform selection	64,231	71,860
Number of transcripts after clustering (‘non-redundant assembly’)	44,693	50,067
N50(bp)	1,527	1,583
Median contig length (bp)	382	391
Average contig lenth (bp)	810.04	830.86
Number of predicted peptides from non-redundant assembly (one peptide/transcript)	20,280	18,000

Supplementary Table 3. Assembly annotation statistics.

	H. amboinensis	*H. tubifera*
**Based on transcripts**		
Number of transcripts	44,693	50,067
Without annotation	30,349	38,603
With UniProt hits + GO annotation	6,643	4,935
With UniProt hits only	7,701	6,529
**Based on predicted peptides**		
Number of peptides	20,280	18,000
UniProt and PFAM		
Without annotation	7,501	6,609
With UniProt hits + GO annotation + PFAM	4,568	3,812
With UniProt hits + GO annotation	441	322
With UniProt hits + PFAM	5,529	5,235
With UniProt hits only	921	704
With Pfam domains only	1,320	1,318
Ensembl Metazoa		
Without annotation	2,876	4,296
Porifera	16,031	11,421
Other metazoa (Placozoa, Cnidaria, Ctenophora)	154	258
Bilateria	1,219	2,025

Supplementary Table 4. Percent of *A. queenslandica* proteins with sequence similarity (Blastp e-value <1x10^{-5}) to predicted peptides in other sponge species.

	Number of *A. queenslandica* peptides with Blastp match (percent of total peptides)
Haliclona amboinensis	22,230 (86%)
Haliclona tubifera	21,590 (84%)
Petrosia ficiformis	21,405 (83%)
Ephydatia muelleri	21,099 (82%)
Oscarella carmela	18,725 (73%)
Leucoselenia complicata	19,025 (74%)
Sycon ciliatum	18,759 (73%)

Supplementary Table 5. Estimated number of gene losses and gains based on EvolMap analysis of 8 sponge species.

Ancestor name*	Sym-bets	Present loci	Losses	Paralogs	Diverged paralogs	Ambiguous gains	Total gains
Aque_Hamb_Pfic_Htub_Emue_Lcom_Scil_Ocar	7,562						
Aque_Hamb_Pfic_Htub_Emue_Lcom_Scil	7,407	10,014	43	377	1,680	438	2,495
Aque_Hamb_Pfic_Htub_Emue	7,892	10,253	1,800	548	1,063	428	2,039
Aque_Hamb_Pfic_Htub	9,510	12,062	658	618	1,343	506	2,467
Aque_Hamb	8,160	11,988	1,584	624	752	134	1,510
Aque	25,796	24,563	1,858	8,417	3,739	2,277	14,433
Hamb	20,280	19,107	1,970	3,020	3,421	2,648	9,089
Pfic_Htub	7,279	11,967	1,321	222	780	224	1,226
Pfic	17,979	15,704	1,695	989	1,650	2,793	5,432
Htub	18,000	16,793	2,993	2,603	2,810	2,406	7,819
Emue	20,527	18,863	1,793	5,484	2,997	1,922	10,403
Lcom_Scil	13,675	16,286	983	1,911	3,087	2,257	7,255
Lcom	90,512	72,127	274	13,348	14,891	27,876	56,115
Scil	50,097	43,313	2,337	12,702	8.215	8,447	29,364
Ocar	21,472	19,578	0	4,870	4,069	3,077	12,016

**A. queenslandica* (Aque); *H. amboinensis* (Hamb); *H. tubifera* (Htub); *P. ficiformis* (Pfic); *E. muelleri* (Emue); *L. complicata* (Lcom); *S. ciliatum* (Scil); *O. carmela* (Ocar)

Supplementary Table 6. Accession numbers of COI sequences used for phylogenetic analysis.

Species	Accession numbers
Amphimedon queenslandica	NC008944.1
Callyspongia armigera	EF519578.1
Callyspongia vaginalis	EF519580.1
Calyx podatypa	JX999086.1
Ephydatia muelleri	DQ176778.1
Eunapius subterraneus	FJ715439.1
Haliclona coerulea	EF519619.1
Haliclona amboinensis 1340729	KR707685
Haliclona amboinensis 1340730	KR707686
Haliclona amboinensis 1498399	KR707687
Haliclona amboinensis 1498400	KR707688
Haliclona amboinensis 1498401	KR707689
Haliclona aqueductus	EF095186.1
Haliclona manglaris	EF519626.1
Haliclona simulans	JN242201.1
Haliclona sp.	JN242210.1
Haliclona tubifera 1340734	KR707690
Haliclona tubifera 1340735	KR707691
Haliclona tubifera 1498402	KR707692
Haliclona tubifera 1498403	KR707693
Haliclona tubifera 1498404	KR707694
Haliclona tubifera	EF519624.1
Haliclona vanderlandi	JN242208.1
Hemigellius fimbriatus	JN242211.1
Lubomirskia baicalensis	EU000568.1
Neopetrosia seriata	JN242213.1
Niphates alba	EF519654.1
Niphates erecta	EF519661.1
Petrosia dura	EF519663.1
Petrosia ficiformis	JX999088.1
Petrosia sp.	JN242219.1
Spongilla vastus	DQ167179.1
Xestospongia muta	EF519700.1

Supplementary Note

Nucleotide and amino acid sequences used for phylogenetic analyses (fasta format). Sequences were aligned using ClustalW and trimmed using Gblocks.

Cytochrome oxidase I (COI)

>Niphates_erecta|EF519661.1_COI
ACTTCTTTTAGTTTATTAATACGATTAGAATTGGCTCGTCCGGGGCCTATGTTGGGGAAT
GATCAGTTGTACAATAGTATTGTGACTGCGCATGCATTTGTGATGATATTTTTTATGGTA
ATGCCAGTGATGATCGGAGGTTTTGGTAATTGATTTGTACCTTTGTTTATAGGGGCCCCA
GATATGGCATTTCCCAGGTTGAATAATATTAGTTTTTGGTTGTTGCCGCCAGCTTTATTA
TTACTATTGAGTTCTGCATTTGTAGAACAAGGAGCTGGAACAGGGTGAACAGTATATCCA
CCCTTGTCTGGGGCACAAGCACACTCTGGGGGATCCGTGGATCTGGTGATATTTAGTTTG
CATTTAGCAGGGATATCATCAATATTGGGTGCAATGAATTTTATAACTACGATTTTAAAT
ATGAGGGC
>Niphates_alba|EF519654.1_COI
ACTTCTTTTAGTTTATTAATACGATTAGAATTGGCTCGTCCGGGGCCTATGTTGGGGAAT
GATCAGTTGTACAATAGTATTGTGACTGCGCATGCATTTGTGATGATATTTTTTATGGTA
ATGCCAGTGATGATCGGAGGTTTTGGTAATTGATTTGTACCTTTGTTTATAGGGGCCCCA
GATATGGCATTTCCCAGGTTGAATAATATTAGTTTTTGGTTGTTGCCGCCAGCTTTATTA
TTACTATTGAGTTCTGCATTTGTAGAACAAGGAGCTGGAACAGGGTGAACAGTATATCCA
CCCTTGTCTGGGGCACAAGCACACTCTGGGGGATCCGTGGATCTGGTGATATTTAGTTTG
CATTTAGCAGGGATATCATCAATATTGGGTGCAATGAATTTTATAACTACGATTTTAAAT
ATGAGGGC
>Haliclona_coerulea|EF519619.1_COI
ACGGCATTTAGTATGCTAATAAGGTTGGAATTGTCTGCGCCAGGGCCAATGTTGGGGAAT
GACCAATTATATAATGTGATAGTGACAGCACATGCATTTGTGATGATATTTTTTATGGTG
ATGCCGGTAATGATCGGTGGGTTTGGGAATTGATTGGTGCCGTTGTATATAGGGGCACCG
GACATGGCGTTTCCACGACTAAATAACATAAGTTTCTGGTTATTGCCGCCCGCCTTAGCC
TTACTACTAGGTTCGGCTTTTGTAGAACAAGGGGCGGGGACGGGGTGAACAGTATATCCG
CCACTGGCAAGCATACAAGCGCATTCGGGGGGGTCAGTAGATATGGTGATATTTAGCTTA
CATTTAGCGGGGATATCGTCGATATTGGGTGCAATGAATTTTATTACAACAATATACAAC
ATACGAGT
>Amphimedon_queenslandica|NC_008944.1_COI
ACGGCATTTAGTATGTTAATCAGATTGGAATTATCCGCGCCAGGGCCGATGTTGGGGAAC
GACCAATTATATAATGTGATAGTGACGGCACATGCATTTGTGATGATATTTTTTATGGTG
ATGCCGGTAATGATAGGGGGATTTGGGAATTGATTAGTTCCGTTGTATATAGGGGCGCCG
GACATGGCGTTTCCACGACTAAATAACATAAGTTTCTGGTTATTGCCGCCCGCCTTAGCC
TTATTATTAGGTTCGGCCTTTGTAGAACAAGGGGCGGGTACGGGGTGAACAGTATATCCA
CCACTGGCAAGCATACAAGCACATTCGGGGGGGTCAGTAGATTTGGTGATATTTAGCTTG
CATTTAGCGGGGATATCGTCTATATTGGGTGCAATGAATTTTATTACAACAATATTCAAC
ATGCGAGC
>Callyspongia_armigera|EF519578.1_COI
ACGGCATTTAGTATGTTAATCAGATTGGAATTATCCGCGCCAGGGCCAATGCTGGGTAAC
GACCAATTATATAATGTGATTGTGACGGCACATGCATTTGTGATGATATTTTTTATGGTG
ATGCCGGTAATGATAGGGGGATTTGGGAATTGAATGGTACCGCTGTATATAGGGGCACCG
GACATGGCGTTTCCCCGATTAAATAACATAAGTTTCTGGTTATTGCCGCCCGCCTTGGCC
CTATTATTAGGTTCGGCCTTTGTAGAACAGGGGGCGGGAACAGGGTGAACAGTATATCCA
CCGCTGTCAAGCATACAATCCCATTCGGGGGGGTCAGTAGACTTGGTGATATTCAGCTTA
CATTTAGCGGGGATATCGTCGATATTGGGTGCAATGAATTTTATTACAACAATATTCAAC
ATGCGATC
>Haliclona_simulans|JN242201.1_COI
ACGGCATTTAGTATGTTAATCAGATTGGAATTATCAGCGCCAGGGCCAATGTTGGGGAAC

GACCAATTATATAATGTGATTGTGACGGCACATGCATTTGTGATGATATTTTTTATGGTG
ATGCCGGTAATGATAGGGGGATTTGGGAATTGAATGGTGCCGCTGTATATAGGGGCACCG
GACATGGCGTTTCCACGACTAAATAACATAAGTTTCTGGTTATTGCCGCCCGCCTTAGCC
TTATTATTAGGTTCGGCCTTTGTAGAACAAGGGGCGGGAACAGGGTGAACAGTATATCCA
CCGCTGTCAAGCATACAATCCCATTCGGGGGGGTCAGTAGACTTGGTGATATTCAGCTTA
CATTTAGCGGGGATATCGTCGATATTGGGTGCAATGAATTTTATTACAACAATATTCAAC
ATGCGAGC
>Callyspongia_vaginalis|EF519580.1_COI
ACGGCATTTAGTATGTTAATCAGATTGGAATTATCAGCGCCAGGGCCAATGTTGGGGAAC
GATCAATTATATAATGTGATTGTGACGGCACATGCATTTGTGATGATATTTTTTATGGTG
ATGCCGGTAATGATAGGGGGATTTGGGAATTGAATGGTACCGCTGTATATAGGGGCACCG
GACATGGCGTTTCCCCGACTAAATAACATAAGTTTCTGGTTATTGCCGCCCGCCTTAGCC
TTATTATTAGGTTCGGCCTTTGTAGAACAGGGGGCGGGAACAGGGTGAACAGTATATCCG
CCGCTGTCAAGCATACAATCCCATTCGGGGGGGTCAGTAGACTTGGTGATATTCAGCTTA
CATTTAGCGGGGATATCGTCGATATTGGGTGCAATGAATTTTATTACAACAATATTCAAC
ATGCGAGC
>Haliclona_sp|JN242210.1_COI
ACAGCCTTTAGCATGCTTATAAGATTGGAATTATCAGCACCTGGGTCAATGTTGGGGGAC
GATCATTTATACAATGTAATAGTGACTGCACATGCGTTTGTAATGATATTTTTTTTGGTA
ATGCCAGTAATGATAGGTGGATTTGGTAATTGGTTAGTTCCTTTGTATATTGGTGCGCCA
GATATGGCATTTCCTCGATTAAATAATATTAGTTTTTGATTATTACCTCCTGCTTTAACT
TTATTAGTTGGTTCTGCTTTTGTCGAACAAGGAGCAGGAACAGGATGAACAGTATACCCT
CCATTATCTAGTATTCAAACACATTCGGGGGGGTCTGTCGATATGGTAATATTTAGTCTT
CATTTAGCTGGGATTTCTTCTATATTGGGGGCAATGAATTTTATTACAACTATTTTTAAT
ATGAGAAC
>Haliclona_aqueductus|EF095186.1_COI
ACAGCATTTAGCATGCTTATAAGATTGGAATTATCAGCACCTGGGTCAATGTTGGGGGAC
GATCATTTATACAATGTAATAGTGACTGCACATGCGTTTGTAATGATATTTTTTTTGGTA
ATGCCAGTAATGATAGGTGGATTTGGTAATTGGTTAGTTCCTTTGTATATTGGTGCGCCA
GATATGGCATTTCCTCGATTAAATAATATTAGTTTTTGATTATTACCTCCTGCTTTAACT
TTATTAGTTGGTTCTGCTTTTGTCGAACAAGGAGCAGGAACAGGATGAACAGTATACCCT
CCATTATCTAGTATTCAAACACATTCGGGGGGGTCTGTCGATATGGTAATATTTAGTCTT
CATTTAGCTGGGATTTCTTCTATATTGGGGGCAATGAATTTTATTACAACTATTTTTAAT
ATGAGAAC
>Hemigellius_fimbriatus|JN242211.1_COI
ACAGCATTTAGTATGCTGATCAGGCTAGAATTGTCAGCCCCTGGGGCAATGTTGGGGGAT
GATCATTTATATAATGTTATAGTGACAGCCCATGCGTTTGTTATGATATTTTTTTTGGTC
ATGCCAGTAATGATAGGTGGTTTTGGTAATTGGTTGGTTCCTTTGTATATCGGGGCGCCA
GATATGGCATTTCCTCGATTAAATAATATTAGTTTTTGATTATTGCCACCTGCTTTAACA
TTACTATTAGGTTCTGCTTTTGTCGAACAAGGAGCTGGAACMGGATGAACAGTATATCCG
CCATTATCGAGTATACAAGCCCATTCGGGGGGGTCGGTGGATATGACTATATTTAGTCTT
CATTTAGCCGGGGTTTCTTCTATATTGGGGGCAATGAATTTTATTACAACTATTTTTAAT
ATGAGGGC
>Eunapius_subterraneus|FJ715439.1_COI
ACAGCATTTAGTATGCTTATAAGATTAGAGCTATCAGCCCCCGGGTCAATGTTAGGGGAT
GATCAATTATATAATGTTATAGTTACAGCCCATGCTTTTCTAATGATATTTTTCTTAGTT
ATGCCAGTAATGATTGGGGGATTTGGAAATTGATTCGTGCCATTATATATTGGTGCACCC
GATATGGCTTTTCCAAGATTAAACAATATTAGTTTTTGATTATTACCTCCGGCTTTAACT
CTATTATTAGGATCTGCTTTTGTAGAGCAAGGGGTTGGTACAGGATGGACAGTATATCCC
CCTTTAGCAGGCATACAAGCGCATTCTGGGGGATCGGTTGATATGGCAATATTTAGTCTT
CACTTGGCGGGTATTTCTTCGATATTAGGGGCTATGAATTTTATCACAACAATCTTTAAT
ATGAGAGC
>Lubomirskia_baicalensis|EU000568.1_COI
ACAGCATTTAGTATGCTTATAAGATTAGAGCTATCAGCCCCTGGGTCAATGTTAGGGGAT
GATCAATTATATAATGTTATAGTTACAGCCCATGCTTTTCTAATGATATTTTTCTTAGTT
ATGCCAGTAATGATTGGGGGATTTGGAAATTGATTCGTGCCATTATATATTGGTGCACCC
GATATGGCTTTTCCAAGATTAAACAATATTAGTTTTTGATTATTACCTCCGGCTTTAACT

CTATTATTAGGATCTGCTTTTGTAGAGCAAGGGGTTGGTACAGGATGGACAGTATATCCC
CCTTTAGCAGGCATACAAGCGCATTCTGGGGGATCGGTTGATATGGCAATATTTAGTCTT
CACTTGGCGGGTATTTCTTCGATATTAGGGGCTATGAATTTTATCACAACAATCTTTAAT
ATGAGAGC
>Ephydatia_muelleri|DQ176778.1_COI
ACAGCATTTAGTATGCTTATAAGATTAGAGCTATCAGCCCCTGGGTCAATGTTAGGGGAT
GATCAATTATATAATGTTATAGTTACAGCCCATGCTTTTCTAATGATATTTTTCTTAGTT
ATGCCAGTAATGATTGGGGGATTTGGAAATTGATTCGTGCCATTATATATTGGTGCACCC
GATATGGCTTTTCCAAGATTAAACAATATTAGTTTTTGATTATTACCTCCGGCTTTAACT
CTATTATTAGGATCTGCTTTTGTAGAGCAAGGGGTTGGTACAGGATGGACAGTATATCCC
CCTTTAGCAGGCATACAAGCGCATTCTGGGGGATCGGTTGATATGGCAATATTTAGTCTT
CACTTGGCGGGTATTTCTTCGATATTAGGGGCTATGAATTTTATCACAACAATCTTTAAT
ATGAGAGC
>Spongilla_vastus|DQ167179.1_COI
ACAGCATTTAGTATGCTTATAAGATTAGAGCTATCAGCCCCTGGGTCTATGTTAGGGGAT
GATCAATTATATAATGTTATAGTTACAGCCCATGCTTTTATAATGATATTTTTCTTAGTT
ATGCCAGTAATGATTGGGGGATTTGGAAATTGATTCGTGCCACTATATATTGGTGCACCC
GATATGGCTTTTCCAAGATTAAATAATATTAGTTTTTGATTATTACCTCCGGCTTTAACT
CTATTATTAGGATCTGCTTTTGTAGAGCAAGGGGTTGGTACAGGATGGACAGTATATCCC
CCTTTAGCAGGCATACAAGCACATTCTGGGGGATCAGTTGATATGGCAATATTTAGTCTT
CACTTGGCGGGTATTTCTTCGATATTAGGGGCTATGAATTTTATCACTACAATCTTTAAT
ATGAGAGC
>Haliclona_vanderlandi|JN242208.1_COI
ACTGCCTTTAGTATGTTAATTAGATTGGAACTATCCGCTCCAGGGTCTATGTTAGGAGAT
GATCATTTATATAATGTTATAGTAACAGCCCATGCATTTGTAATGATATTTTTTTTAGTT
ATGCCAGTAATGATAGGGGGTTTTGGTAATTGGCTAGTACCATTATACATTGGTGCCCCG
GATATGGCATTTCCTAGATTAAATAATATTAGTTTTTGGTTATTACCTCCAGCTCTTACT
TTATTGTTAGGTTCTGCTTTTGTAGAACAGGGAGCAGGGACGGGTTGAACGGTTTATCCG
CCTTTATCTAGTATTCAAACCCATTCTGGGGGATCTGTGGACATGGTAATATTTAGTCTT
CATTTAGCGGGGATATCTTCTATATTGGGAGCTATGAACTTTATAACAACAATCTTTAAT
ATGAGAGC
>Calyx_podatypa|JX999086.1_COI
ACAGCATTTAGTATGCTTATTAGGTTAGAACTATCTTCCCCAGGGTCTATGTTAGGAGAT
GATCATTTATATAATGTTATAGTAACAGCTCATGCATTTGTAATGATATTTTTTTTAGTT
ATGCCAGTAATGATTGGGGGTTTTGGTAATTGGTTAGTACCTTTATATATTGGTGCCCCG
GATATGGCTTTTCCTAGATTAAATAATATTAGTTTTTGGTTATTACCTCCGGCGCTTACT
TTATTATTAGGTTCGGCTTTTGTAGAACAAGGGGCTGGGACAGGTTGGACAGTTTATCCG
CCTTTATTTAGTATTCAAACTCATTCTGGGGGGTCTGTGGATATGGTAATATTTAGTTTA
CATTTAGCTGGAATATCTTCTATATTAGGGGCTATGAATTTTATAACAACAATCTTTAAT
ATGAGGTC
>Haliclona_tubifera|EF519624.1_COI
ACAGCTTTTAGTATGCTTATTAGGTTAGAATTATCGGCTCCGGGGTCTATGTTAGGGGAT
GATCATTTATATAATGTTATAGTAACAGCTCACGCATTTGTAATGATATTTTTTTTAGTT
ATGCCAGTAATGATAGGGGGTTTTGGTAATTGGTTAGTACCACTATATATTGGTGCCCCA
GATATGGCTTTTCCTAGATTAAATAATATTAGTTTTTGGTTGTTACCCCCAGCGCTTACT
TTATTATTAGGTTCGGCTTTTGTGGAACAAGGGGCTGGAACAGGTTGAACGGTTTATCCT
CCTCTATCTAGTATTCAAACCCATTCTGGGGGATCTGTGGATATGGTGATATTTAGTCTT
CATTTAGCAGGAATATCTTCTATATTGGGTGCTATGAATTTTATAACAACAATATTTAAT
ATGAGGGC
>Haliclona_manglaris|EF519626.1_COI
ACAGCATTTAGTATGCTTATTAGATTAGAACTATCTGCCCCAGGGTCAATGTTAGGGGAT
GATCATTTATATAACGTTATAGTAACAGCTCATGCATTTGTAATGATATTTTTTTTAGTT
ATGCCAGTAATGATAGGGGGTTTTGGAAATTGGTTAGTTCCATTATATATTGGAGCTCCA
GATATGGCTTTCCCTAGGTTAAATAATATTAGTTTCTGATTATTACCTCCTGCGCTTACT
TTATTGTTAGGTTCTGCTTTTGTGGAGCAAGGGGCTGGGACAGGTTGAACAGTTTATCCG
CCTTTATCTAGTATTCAAGCCCATTCTGGGGGATCTGTGGATATGGTAATATTTAGTCTT
CATTTAGCGGGTATATCTTCTATATTGGGGGCGATGAACTTTATAACAACAATTTTTAAT

ATGAGGGC
>Petrosia_dura|EF519663.1_COI
ACAGCATTTAGTATGCTTATAAGATTAGAACTATCTGCCCCAGGGGCTATGTTAGGGGAT
GATCATTTATATAATGTTATAGTGACTGCTCATGCATTTGTAATGATATTTTTTTTAGTT
ATGCCAGTAATGATCGGTGGTTTTGGTAATTGATTAGTTCCATTATATATTGGTGCTCCA
GATATGGCTTTCCCTAGATTAAATAATATTAGTTTTTGATTATTACCTCCCGCTCTAACT
TTATTATTAGGTTCTGCTTTTGTAGAACAAGGGGCTGGGACAGGATGAACAGTCTATCCA
CCTTTATCTAGTATTCAAACACATTCGGGGGGGTCTGTAGATATGGTAATATTTAGTCTT
CATTTAGCAGGGATATCTTCTATATTGGCGGCGATGAACTTTATAACAACTATCTTTAAT
ATGAGGGC
>Petrosia_ficiformis|JX999088.1_COI
ACAGCATTTAGTATGCTTATAAGATTAGAACTATCTGCCCCAGGGGCTATGTTAGGGGAT
GATCATTTATATAATGTTATAGTGACTGCTCATGCATTTGTAATGATATTTTTTTTAGTT
ATGCCAGTAATGATCGGTGGTTTTGGTAATTGATTAGTTCCATTATATATTGGTGCTCCA
GATATGGCTTTCCCTAGATTAAATAATATTAGTTTTTGATTATTACCTCCCGCTCTAACT
TTATTATTAGGTTCTGCTTTTGTAGAACAAGGGGCTGGGACAGGATGAACAGTCTATCCA
CCTTTATCTAGTATTCAAACACATTCGGGGGGGTCTGTAGATATGGCAATATTTAGTCTT
CATTTAGCAGGGATATCTTCTATATTGGGGGCGATGAACTTTATAACAACTATCTTTAAT
ATGAGGGC
>Neopetrosia_seriata|JN242213.1_COI
ACAGCATTTAGTATGCTTATTAGATTAGAACTATCTGCCCCAGGGGCTATGTTAGGGGAT
GATCATTTATATAATGTTATAGTGACAGCTCACGCATTTGTAATGATATTTTTTTTAGTT
ATGCCAGTAATGATCGGCGGTTTTGGTAATTGATTAGTTCCATTATATATTGGTGCCCCA
GATATGGCTTTCCCTAGATTAAATAATATTAGTTTTTGATTATTACCTCCAGCTCTAACT
TTATTATTAGGTTCTGCTTTTGTAGAACAAGGGGCCGGAACAGGGTGAACGGTCTACCCM
CCTTTATCTAGCATTCAAACACATTCMGGGGGGGTCTGTCGATATGGTAATATTTAGTCTT
CATTTAGCAGGGATATCTTCTATATTAGGGGSTATGAACTTTATAACAACTATCTTTAAT
ATGAGGGC
>Petrosia_sp.|JN242219.1_COI
ACAGCATTTAGTATGCTTATTAGATTAGAACTATCTGCCCCAGGGGCGATGTTAGGGGAT
GATCATTTATATAATGTTATAGTGACAGCTCACGCATTTGTAATGATATTTTTTTTAGTT
ATGCCAGTAATGATTGGCGGTTTTGGTAATTGATTAGTTCCATTATATATTGGTGCCCCA
GATATGGCTTTCCCTAGATTAAATAATATTAGTTTTTGATTATTACCTCCAGCTCTAACT
TTATTATTAGGTTCTGCTTTTGTAGAACAAGGGGCCGGAACAGGGTGAACGGTATACCCA
CCTTTATCTAGCATTCAAACACATTCGGGGGGGTCTGTAGATATGGTAATATTTAGTCTT
CATTTAGCAGGGATATCTTCTATATTAGGGGCTATGAACTTTATAACAACTATCTTTAAT
ATGAGGGC
> Haliclona_tubifera_1498403|KR707693_COI
ACAGCGTTTACTATGCTTATTAGATTGGAACTATCTGCTCCGGGGTCTATGTTAGGGGAT
GATCATTTATATAATGTTATAGTAACAGCTCATGCATTTGTAATGATATTTTTTTTAGTT
ATGCCAGTAATGATAGGGGGTTTTGGTAATTGGTTAGTTCCATTATATATTGGGTCTCCC
GATATGGCTTTTCCTAGATTAAATAATATTAGTTTCTGGTTGTTACCCCCGGCGCTTACT
TTATTGTTAGGTTCTGCTTTTGTGGAACAAGGTGCGGGGACAGGTTGAACAGGGTATCCA
CCTTTACCAAGTATTCAAACTCATTCTGGGGGATCTGTGGATATGGTGATATTTAGTCTT
CATTTAGCAGGGATATCTTCAATATTGGGGGCTATGAATTTTATAACTACAATCTTTAAT
ATGAGGGC
>Haliclona tubifera_1340734|KR707690_COI
ACAGCGTTTAGTATGCTTATTAGATTGGAACTATCTGCTCCGGGGTCTATGTTAGGGGAT
GATCATTTATATAATGTTATAGTAACAGCTCATGCATTTGTAATGATATTTTTTTTAGTT
ATGCCAGTAATGATAGGGGGTTTTGGTAATTGGTTAGTTCCATTATATATTGGTGCTCCC
GATATGGCTTTTCCTATATTAAATAATATTAGTTTCTGGTTGTTACCCCCGGCGCTTACC
TTATTGTTAGGTTCTGCTTTTGTGGAACAAGGTGCGGGGACAGGTTGAACAGTGTATCCA
CCTTTATCAAGTATTCAAACTCATTCTGGGGGATCTGTGGATATGGTGATATTTAGTCTT
CATTTACCAGGGATATCTTCAATATTGGGGGCTATGAATTTTATAACTACAATCTTTAAT
ATGAGGGC
>Haliclona_tubifera_1340735|KR707691_COI
ACAGCGTTTAGTATGCTTATTAGATTGGAACTATCTGCTCCGGGGTCTATGTTAGGGGAT

GATCATTTATATAATGTTATAGTAACAGCTCATGCATTTGTAATGATATTTTTTTTAGTT
ATGCCAGTAATGATAGGGGGTTTTGGTAATTGGTTAGTTCCATTATATATTGGTGCTCCC
GATATGGCTTTTCCTAGATTAAATAATATTAGTTTCTGGTTGTTACCCCCGGCGCTTACC
TTATTGTTAGGTTCTGCTTTTGTGGAACAAGGTGCGGGGACAGGTTGAACAGTGTATCCA
CCTTTATCAAGTATTCAAACTCATTCTGGGGGATCTGTGGATATGGTGATATTTAGTCTT
CATTTAGCAGGGATATCTTCAATATTGGGGGCTATGAATTTTATAACTACAATCTTTAAT
ATGAGGGC

>Haliclona_tubifera_1498402|KR707692_COI

ACAGCGTTTAGTATGCTTATTAGATTGGAACTATCTGCTCCGGGGTCTATGTTAGGGGAT
GATCATTTATATAATGTTATAGTAACAGCTCATGCATTTGTAATGATATTTTTTTTAGTT
ATGCCAGTAATGATAGGGGGTTTTGGTAATTGGTTAGTTCCATTATATATTGGTGCTCCC
GATATGGCTTTTCCTAGATTAAATAATATTAGTTTCTGGTTGTTACCCCCGGCGCTTACC
TTATTGTTAGGTTCTGCTTTTGTGGAACAAGGTGCGGGGACAGGTTGAACAGTGTATCCA
CCTTTATCAAGTATTCAAACTCATTCTGGGGGATCTGTGGATATGGTGATATTTAGTCTT
CATTTAGCAGGGATATCTTCAATATTGGGGGCTATGAATTTTATAACTACAATCTTTAAT
ATGAGGGC

>Haliclona_tubifera_1498404|KR707694_COI

ACAGCGTTTAGTATGCTTATTAGATTGGAACTATCTGCTCCGGGGTCTATGTTAGGGGAT
GATCATTTATATAATGTTATAGTAACAGCTCATGCATTTGTAATGATATTTTTTTTAGTT
ATGCCAGTAATGATAGGGGGTTTTGGTAATTGGTTAGTTCCATTATATATTGGTGCTCCC
GATATGGCTTTTCCTAGATTAAATAATATTAGTTTCTGGTTGTTACCCCCGGCGCTTACC
TTATTGTTAGGTTCTGCTTTTGTGGAACAAGGTGCGGGGACAGGTTGAACAGTGTATCCA
CCTTTATCAAGTATTCAAACTCATTCTGGGGGATCTGTGGATATGGTGATATTTAGTCTT
CATTTAGCAGGGATATCTTCAATATTGGGGGCTATGAATTTTATAACTACAATCTTTAAT
ATGAGGGC

>Haliclona_amboinensis_1340729|KR707685_COI

ACAGCATTTAGTATGCTTATAAGGTTAGAGTTATCAGCTCCTGGGGCAATGTTGGGGGAC
GATCATTTATATAATGTTATAGTGACAGCCCATGCGTTTGTAATGATCTTTTTTTTGGTT
ATGCCAGTAATGATAGGTGGTTTTGGTAATTGGTTAGTTCCTTTGTATATTGGGGCGCCA
GATATGGCATTTCCTCGATTAAATAATATAAGTTTTTGATTATTACCGCCTGCTTTAACT
TTACTATTAGGTTCTGCTTTTGTTGAACAAGGAGCTGGAACAGGATGAACAGTATATCCG
CCGTTATCGAGTATCCAAACACATTCGGGGGGGTCGGTTGATATGGTAATCTTTAGTCTT
CATTTAGCGGGGATTTCTTCTATATTGGGGGCAATGAATTTTATTACAACTATCTTTAAT
ATGAGGGC

>Haliclona_amboinensis_1498399|KR707687_COI

ACAGCATTTAGTATGCTTATAAGGTTAGAGTTATCAGCTCCTGGGGCAATGTTGGGGGAC
GATCATTTATATAATGTTATAGTGACAGCCCATGCGTTTGTAATGATCTTTTTTTTGGTT
ATGCCAGTAATGATAGGTGGTTTTGGTAATTGGTTAGTTCCTTTGTATATTGGGGCGCCA
GATATGGCATTTCCTCGATTAAATAATATAAGTTTTTGATTATTACCGCCTGCTTTAACT
TTACTATTAGGTTCTGCTTTTGTTGAACAAGGAGCTGGAACAGGATGAACAGTATATCCG
CCGTTATCGAGTATCCAAACACATTCGGGGGGGTCGGTTGATATGGTAATCTTTAGTCTT
CATTTAGCGGGGATTTCTTCTATATTGGGGGCAATGAATTTTATTACAACTATCTTTAAT
ATGAGGGC

>Haliclona_amboinensis_1498400|KR707688_COI

ACAGCATTTAGTATGCTTATAAGGTTAGAGTTATCAGCTCCTGGGGCAATGTTGGGGGAC
GATCATTTATATAATGTTATAGTGACAGCCCATGCGTTTGTAATGATCTTTTTTTTGGTT
ATGCCAGTAATGATAGGTGGTTTTGGTAATTGGTTAGTTCCTTTGTATATTGGGGCGCCA
GATATGGCATTTCCTCGATTAAATAATATAAGTTTTTGATTATTACCGCCTGCTTTAACT
TTACTATTAGGTTCTGCTTTTGTTGAACAAGGAGCTGGAACAGGATGAACAGTATATCCG
CCGTTATCGAGTATCCAAACACATTCGGGGGGGTCGGTTGATATGGTAATCTTTAGTCTT
CATTTAGCGGGGATTTCTTCTATATTGGGGGCAATGAATTTTATTACAACTATCTTTAAT
ATGAGGGC

>Haliclona_amboinensis_1340730|KR707686_COI

ACAGCATTTAGTATGCTTATAAGGTTAGAATTATCAGCTCCTGGGGCAATGTTGGGGGAC
GATCATTTATATAATGTTATAGTGACAGCCCATGCGTTTGTAATGATCTTTTTTTTGGTT
ATGCCAGTAATGATAGGTGGTTTTGGTAATTGGTTAGTTCCTTTGTATATTGGGGCGCCA
GATATGGCATTTCCTCGATTAAATAATATAAGTTTTTGATTATTACCGCCTGCTTTAACT

TTACTATTAGGTTCTGCTTTTGTTGAACAAGGAGCTGGAACAGGATGAACAGTATATCCG
CCGTTATCGAGTATCCAAACACATTCGGGGGGGTCGGTTGATATGGTAATCTTTAGTCTT
CATTTAGCGGGGATTTCTTCTATATTGGGGGCAATGAATTTTATTACAACTATCTTTAAT
ATGAGGGC
>Haliclona_amboinensis_1498401|KR707689_COI
ACAGCATTTAGTATGCTTATAAGGTTAGAATTATCAGCTCCTGGGGCAATGTTGGGGGAC
GATCATTTATATAATGTTATAGTGACAGCCCATGCGTTTGTAATGATCTTTTTTTTGGTT
ATGCCAGTAATGATAGGTGGTTTTGGTAATTGGTTAGTTCCTTTGTATATTGGGGCGCCA
GATATGGCATTTCCTCGATTAAATAATATAAGTTTTTGATTATTACCGCCTGCTTTAACT
TTACTATTAGGTTCTGCTTTTGTTGAACAAGGAGCTGGAACAGGATGAACAGTATATCCG
CCGTTATCGAGTATCCAAACACATTCGGGGGGGTCGGTTGATATGGTAATCTTTAGTCTT
CATTTAGCGGGGATTTCTTCTATATTGGGGGCAATGAATTTTATTACAACTATCTTTAAT
ATGAGGGC

Glutamate GPCRs

>Haliclona_tubifera|Htub_mm_72_glutamate
AVHLVNEDPDLLPGLKLGFTQPNRALGSASSSESISAASYASTARILSDKTRYEYFLRTL
PLVLHYNWAEGLFKLTFLMS----DAVYWESVDAVLAFAHAIDEIDFALVFLICYDHSTV
KASSRELSAISLTGLFLCFSIAFSLCYAALLVKTNRIHRIFNNKISPQSYNIILLIITMA
YAFRSRKIPE-NFNEARFIFVNVCILFFLYFPK
>Amphimedon_queenslandica|PAC1571587_glutamate
AVELVNNDTELLPGIQLGFINSNYALGAASSSVSIAVASYASTARFLSDKTRFDYFLRTI
PIVVNYNWAEGVLRMNFIVS----DAVYWSALDAVYAFAHALSEITFGIVFIVNYNHSTV
KASSRELSTVLLIGLILCYLLSFSLCYAALLVKVNRIHRIFNRQISPHSYNFLLLIISTY
YAFRTRKIPQ-NFNEARFINLTLYSLCCIFFSK
>Petrosia_ficiformis|Pfic_m_102_glutamate
TIDLVNNDSTLLPGIKLGFNSENIGLGAAASAVSVPVASYASSSARLNNRDRYSYFFRTI
SLCIKFNWAQKLFDFLWLAS----DAWFYEAYDSVYSIAHALNKIQFSIVFGIFWNTPIV
KSSGREQMILLLVGVTMCFVWCLSLIFGALLTKLIRIARIFLRGTAPIFYDTILVIACNV
LAILTIRFPD-NFNEARYVSFTTFALACLFGPR
>Haliclona_amboinensis|Hamb_m_336_glutamate
ALDTINNDPDLLPGITLGYNSENIGLGAAASGVSVPVASYASSSARLNNRDRYGYFFRTI
SLCIRFNWAQSLFMFLWLAS----DAWFYEAFDAVYSIAHSLRRISFVIVMAIYWNTPIV
KSSGREQMILLLIGIASCFLSCLSLILGSLLIKLIRISRIFLRSTGPHYYHTVLIIVCNI
LAVLSIRFPD-NFNESKYVAFSTFALLCMFGPR
>Amphimedon_queenslandica|PAC1572115_glutamate
ALDIINSNASLLHGLKLGYSSENIGLGAASSGVSVPVASYASSSARLDNRDRYGYFYRTI
SLCMEFNWARSLFSFLWIAS----DAWFYQSYDAVYSIAFAIRHITFSVALGLYWNTPIV
KSSGREQMILLLVGIALTFLTCISFILASLLVKLIRISRIFLRQTEPRYYHTALIIFCNI
LAVVTIRFPE-NFNESKYVAFSTFSLLCMFGPR
>Haliclona_tubifera|Htub_m_577_glutamate
ALDLINNDTALLPGIVLGYVSENIALGPTISQVALPVASYSASSPTLSNRDRYGYFYRTH
PLALANNWVEKFMEFVWIGS----SSWYYEDYDAVYSIAHALNTVEFAIAMGIFWKTPVI
KSSGREQMILILSGICMSFLVPFTLMLSALLIKLVRITRIFLNKIDWKYYNGILLVVTNF
IGVMTLQFPA-NFNESRYVTFATLSLLCFFVTR
>Amphimedon_queenslandica|PAC1572115_glutamate
AIDSINSNSSLLPGMSLGYVSENIALGASSSSVSIPVASYASSSSALNNRDRYEYFLRTV
PLIERANWADMLLRKVWIAS----DSTAFKQLDAVYSIAYALNEVSFTSAFIWFWNTPIV
KSSGREQMILVLIGITLCFVS---------------ITRIFMQQITPPYYNTALLITSNA
LAVVTIRFPA-NFNESRYVAFSTFSLLCLFGPR
>Amphimedon_queenslandica|PAC1572115_glutamate
AIDSINSDQNLLPNMTLGYKRENIALGAYESFLSIPVASYGSSSTALSNREFYSYFYRTF
PLILHFGWAETFLKFLWIAS----DAWYYEQYDAVYAAAHAINRVAFTSVFIWFWNTPIV
KSSGREQMMLVLIGITLCLLSSSSLILSALLVKLIRITRIFMQRIAPAYYYSILLITSNV
LAVFTIRFPA-NFNESKYVSFATFSLLCLFGPR

>Haliclona_tubifera|Htub_m_182_glutamate
AVDMINGDNNLLPNVTLGYITENIGLGDYASFISVPVASVAT-SVLLNNRERFGYFYRTV
PLILNFGWVDKLMQFVWIAG----DTWYHNYYDAVYSFAHALNSVFFSIGLIALWNKSII
KSSGREQMMLLLIGIACCFLSSFSLILSALFIKLVRIARIFLRGIQPYYFYAILLVSSNA
LAMITIRFPA-NFNEVRYVAFCTFALVSMFVTR
>Haliclona_amboinensis|Hamb_m_400_glutamate
AIDTVNSDPHLLPNITLGYVSESVALGAIVSSVTVPVASFSSTTPILSDRDRYSYFFRTI
PLILHFGWVQGLLNFLWIAG----DGWFYRFYDAVYSIAHALNEVTFVIVFIWFWNTPII
KSSGREQMILLLIGLTLCFLISYSLILCALLIKLVRIARIFLQTIGPGYYYSALIIASNA
LAILTIRFPA-NFNESKYVAFSTFSLACLFGPR
>Amphimedon_queenslandica|PAC1571772_glutamate
AIDSVNSDPTLLPNLTLGYKRENVAMGAFESFITTPMASYGSSSTALSNRELYSYFFRTF
PLIVHYGWVRSLFTFVWIAS----DTWYYEQYDAVYAAAHAINMVNFTCAFIWFWNTPIV
KSSGREQMILLLIGLTLCFLVCFSLVLSALLVKLIRISRIFLHKTGSIHYFSALLIASNA
LAVLTIRFPA-NFNESKYVSFSTFSLFCLFGPR
>Amphimedon_queenslandica|PAC1571912_glutamate
AIDSVNSDPTLLPNLTLGYKRENVAMGAFESFITIPMASYGSSSTALSNRELYSYFFRTF
PLIVHYGWVRSLLTFVWIAS----DTWYYEQYDAVYAAAHAINWVNFSCVFVRFWNTPIV
KSSGREQMILLLIGLALCFLVCFSLVLSALLVKLIRISRIGFHKIGPIYYISALLIASNV
LAVLTIRFPA-NFNESRYVSFSTFSLGCLFGPR
>Petrosia_ficiformis|Pfic_m_396_glutamate

----------------LCFVLCFSLILSALFVKLVRITRIFLRKIKSQYYYTVLLIASNA
LAMFTIRFPE-NFNEVKYVAFSTFS--------
>Amphimedon_queenslandica|PAC1571370_glutamate

-------MIFILIGITLCFVVCFSIILSALLVKLVRISRIFMQKISPKYYYSILLIASNA
LAILTIRFPQ-NFNESKYVAFSTFSLLCLFAPR
>Haliclona_tubifera|Htub_m_304_glutamate
AVKDINNNPDYLPGYNLTICDPSEGTGGSCSIVTEPTASYSSGSPILSDTQMYPYFYRTY
PLMKTYNWAVVVMCYLWIFPGWYTANWYIKAFDAVWAVAIALGNVTFLVFNTVTIRKPLI
RDSAPLINTVIILGCILLMAAGFTLSFGALFAKTLQVYRVYTNPKIWNFYKGLMTIFGLF
LAYESRNVKYMYLNDSRFVSIAMYILLILYIPK
>Petrosia_ficiformis|Pfic_m_170_glutamate
AVDDINNSPDYLPDYNVAICDASEGTGGSCSIVSEPTASYGSASPILSDRSMYPFFFRTY
PLMNKYGWAVIVMCHLWILPGWYSNEWFQAKYDAVWAIATALGSVIFLIFNIITIMKPLI
ANSAPYINTIIIIGCIIMMSTGFTLSFGALFGKTWQVYRVYTNSKHWNFYKGLLVIFGLF
LAYESRNVKYFYINDSRFVSIAMFILLVLYIPK
>Haliclona_amboinensis|Hamb_mm_55_glutamate
AINAVNNDTSLLPNYELKSCDSSEATGGSCSLSTEPTASYASYSPVLSYRSMYPLFLRTS
PLMKMYDWALVVMCHLWILPAWFSLGWFFEEYDAVWAIALSLGRVRFLVFNIVTVKKPLI
ANSAPLINIVITTGCIVMMATGFTLSFGALFAKTWQVYRVYTKSKIWNFYKGLLVVFGLF
LAYESRNVKYFYINDSRFVSIAMYILLILYIPK
>Amphimedon_queenslandica|PAC1571337_glutamate
ALDDVNNASHILSNYTLTAS----------YVISEPTASYASYSPVLSDQSMYPLFLRTA
PLMATYDWAVLVMCHLWILPGWFSESWYFKEYDAVWATALSLGRVTFLVFQIVTIRKPLM
ANSAPYINIILIIGCIVMMGSGFTLSFGSLFAKTWQVYRVYTKAKMWNFYKGLLVVFGLF
LAYESRNVKYTFINDSRFVSIAMYILMILYIPK
>Amphimedon_queenslandica|PAC1571337_glutamate

--EIRETDTEILM
ANSAPYINIILIIGCIVMMGSGFTLSFGSLFAKTWQVYRVYLDEKMWNFYKGLLLVFGLF
LAYESRNVKYTFINDSRFVSIAMYILLVLYVPK
>Haliclona_amboinensis|Hamb_m_799_glutamate
ALKDISLGNFSRLNFDF--CDPARAAGPPCEDSANHLISYAATTPQLNDETQFKSFFRTV

AAMRNFGWLPTIYCYQWIILGDHVVTDFENQLDATWAIALALTTIQFMFINCYYREKRII
KASSPHVNTLILVGCILGFISMFTLSFGALLAKTWRVRAVFHNPKDYMLYKCILLVLGCF
LATQTRHIKAKFFNDTKYIVIAIYGSSVIFLPK
>Amphimedon_queenslandica|PAC1572795_glutamate
ALSEYNESPFSQLRLTL--CDPARAVGPPCEDSTNELVSYASTTPQLNNATIFRNFFRTV
PLLGYFSWLPNILCYQWIFVGNTEISDFNNS-DAMWAIAISLTMVNFLVINLCFREKKVI
KASSPHINTLILVGCLLGILSMFTLSFGALLAKTWRVGAVFRNPKDYVLYKSFLLLLGCF
LATQTRGIKATLFNDSRFIAIAIYI--------
>Amphimedon_queenslandica|PAC1572828_glutamate
AIQEINNRSSILSNYTISYCHGRTATGCGCSVATIPVALP--------------------
-FAKRLGW------YVWIFYDSPKIDWFTKLHDAIWAMSLGLGPFEFLAFTIIFRKKKIV
KLSSPNLNYLLILGSVILYSHGYSLCFSVILAKSGRVYYIFANPKDWMLYRVLLQLVGIF
FAFHTRKIKIKGLKESTQIGYIIYF----LEEK
>Petrosia_ficiformis|Pfic_m_199_glutamate

--------AYNILCYVWFTPGGYTAEWFNKTYDAMWSIAIGLGGVVFLVFNIVFRERKVV
KLSSPNLNYLIIVGSVLLYTCGYTICFAVILSKTWRIYYIFNNPKDWIL-----------

>Amphimedon_queenslandica|PAC1572437_glutamate
ALKLINERNDLLENYTLGYCSYTSSLGCGCSTATIPVAAFASSSLALFDRHRYRNFFRTI
ILMVEFGWSYQVLCYVWILPMWYAPDWFADQYDAVWAIAVGLVSQTILTFNIVYSNKKVL
MANNAKLNYLIIILGSAVLYISGYNLCFGVILSRTWRIYYIFSNIKNWMLYKGILQLCAIF
MAFHMRNVNVKALNESKEIAAIIYILGFTFIPK
>Haliclona_tubifera|Htub_m_344_glutamate
ALEEINNRSDILQNYTLGYCTRTGSLCCGCSPATIPVAAYAAAANELVDRSRFKNFFRTL
PLCRVFNWTYMILCYVWITPMWYNREWFKEEYDTIWAVAIALGQIKYLLFNILFRNKKVV
KLSSPNLNYLIIVGAAILYSCGYTLCFAVILSKTWRIYYIFNNPKDWILYKGLLQLVAIF
MAFHIRKVRVKALNDSKETAAIIYFLGLLFIPK
>Haliclona_tubifera|Htub_mm_82_glutamate

--------TYTILCYVWITPMWYNREWFANEYDTVWSIALALGSIMFLLFNIVFRKKKVV
KLSSPNLNYLIIILGAMLLYLCGYTLCFGVILSKAWRIYYIFSNPKDWLLYKGLLQIVAMF
MAFHTRRIKIKALNDSKEIGAIIYILGLLFIPK
>Petrosia_ficiformis|Pfic_m_190_glutamate

---------------------GYTLCFAVILSKTWRVYYIFNNPKDWFLYKGLLQIIAIF
IAFHIRKVKIKALNDSKEIAAIIYILGLIFIPK
>Amphimedon_queenslandica|PAC1571647_glutamate
ALRLINERNDVLQNYTLNYCDRTMSLGCGCSPATIPVASVVSAANSLDNRIKFRNFYRTS
VLMREFGWAYQLLCYLWIIPMWYNDNWYLGNYDGVWAIAIGLGQVKFFIFNVVFRNKKYA
MMY------------------GYCLCFGVILSKTWRVYHIFNNPKDWFLYKGILQVLAIF
MAFHTRGVKVRILNESKETAAIIYILGLIFIPK
>Amphimedon_queenslandica|PAC1571647_glutamate
ALKLINERKDILQNYTLSYCNRTAGLGCGCSISTIPVASYATGADVLTDRNRFRNFFRTL
VLMREFGWAYQLLCYLWIIPMWYNDDWYFSTYDGVWAIAIGLGQIQFFIFNIAFRSKKVV
KMTSPNLNYIIIILGSAMLYTCGYSLCFGVILSKTWRVYYIFNNPKDWVLYKTMLQLLAIF
MAFHTRRVKVRILNESKEIAAIIYILGLIFIPK
>Haliclona_amboinensis|Hamb_m_433_glutamate
ALQQVNNRTDILQNYTLERCDRTVSLGCGCSTATIPVAAFAAAAYVLNDRNRFRNFFRSL
PLMREFGWAYHIFCYVWILPMWYNDDWFTDQYDGAWAMAIGLGMISFFAFNVFFRNKKVV
KLTSPNLNYFVILGAALLYVCGYNLCFAVILSKTWRIYYIFNNPKDYVLYKGLLQVLAVF
MAFHTRGIKVKVLNESIETTAIIYILGLVFVPK
>Amphimedon_queenslandica|PAC1569924_glutamate

---------------------GYNLCFGVILSKTWRVYYIFNNPKDWVLYKGLLQVSAIF

MAFHTRRVKVKILNESKEIAAIIYILGLIFIPK
>Petrosia_ficiformis|Pfic_m_183_glutamate
ALEAINSDPYILPGYYLTYCARTPSLGCGCSIATVPVGSYASQAPDLIIRDSYPNFYRTV
PIMNLYDWARRVICYVFLTLGWYPSKWFLSEYDALWAFVLAVGEVSFAVLNFVFRKRKYI
RLTSPNLNYLIVLGCALLVVSGYSLCFGTICAKMWRVYFIFHNPKDWQLLKFLLQAAAVI
FAIRTRKVKIKALNDAKEISVIIYILLLLFIPK
>Haliclona_amboinensis|Hamb_m_355_glutamate
ALSAINNDYTLLPGYVLSYCNERTALGSGCSIATEIVGSYASPAPKLNQKSLYPNVFRTV
PLFQQYGWARRVLCYIWLVWGWYPNNWFYNRYDALWAMTLAIGEVSFAIVNFVLRKRKII
RLTSPNLNYLIILGCILLCFSGYSLCFGTICAKMIRVFIIFDNPQDWHLMKFIVQVVSVF
FALKTRKVKIKAINDAKWISIIIYVLLILFIPK
>Amphimedon_queenslandica|PAC1572463_glutamate
ALDYINDRESLLSDYSLSYCDSRVSLGAGCSLATENVGSYASQSSVLNQRSLYRNVFRTV
PLFAEYNWARRVLCYVWLVYGWYPANWFFAMYDALWAVALAIGTVSFAIVNFIFRSRKII
RLTSPNLNYLIVLGCILMSASGYSLCFGTICAKMWRVYFIFHNPKDWHLKMFLVQSAAVF
FAIKTRRVKIKVLNDAKWISIIIYVLCILFIPK
>Petrosia_ficiformis|Pfic_m_197_glutamate
ALDHINKDPNIIKGYKLHYCNHTISLGGGCSIATEPIASCASSSSKLADRTRYKAYFQLL
PICRQYGWAQTLLCYTFLTYGWYSNEWFQKSYDGIWALAIALGPVSFLIFNLIYRKQKLI
RLTSPKLNYLIITGAVIMYISGYSLCFGTILAKMWRVYYIFTNPKDWHL-----------

>Amphimedon_queenslandica|PAC1571675_glutamate

--GPVSFLIFNIFFRKNKLI
RLSSPKLNYFIIAGAIIMYMSGYSLCFGTILAKMWRVYYIFTNPKDWHLFKGVLQVAALF
LAFGTRRVKVKGLNESKFVAAIIYILGLIFIPQ
>Amphimedon_queenslandica|PAC1572401_glutamate
ALDLINEQEDLLPGYKLHYCNHTIALGAGCSVATEPTASCASSSHNLANRVKYKAYFQML
PICLRYRWALTVVCYTFIIYGWYSPTWYHGMYDGVWAFALALGNVSFLVFVIVYKKNKLV
RLTSPKLNVLIIIGSLIMYFSGYTLSFSTILAKMWRVYYIFTNPKDWHLFKAILQVAALF
LAFGIRKVKVRGLNDSKFVGLMVYILGLLFFPQ
>Haliclona_amboinensis|Hamb_mm_67_glutamate

----EYGWARTVICYSFILYGWYSPNWYDVLYDGMWAFALALGEVSFLIFMIACRKSKLI
RLTSPNLNVMVIIGAVVMYISGYTLCFATILAKMWRVYYIFTNPKDWHLYKAILQIAALF
LAFGIRKVKMKGLNDSKFVGVTVYILGLLFIPQ
>Haliclona_amboinensis|Hamb_mm_69_glutamate

--------ARTVICYSFILYGWYSPNWYDVLYDGMWAFALALGEVSFLIFMIACRKSKLI
RLTSPNLNVMVIIGAVVMYISGYTLCFATILAKMWRVYYIFTNPKDWHLYKAILQIAALF
LAFGIRKVKMKGLNDSKFVGVTVYILGLLFIPQ
>Haliclona_tubifera|Htub_mm_22_glutamate
ALDKINDNSSVLPEYNLTYCNRTTSLGAGCSVASKPVASYVSNSDEFRDSRGFPQFIQLY
PAIEIFEWARLVLCYLWITYSWYGQNWFRSEYDATWSIALALGLVQFLVFIVYYRNEKIL
VLGTNVLNYSIIVGVTIILVSGYVFTFSSILTKMGRIYYIYHYPTNFHLYICLLQLFGIV
LVLQTRRIKIRVLNDSKSVSGIIYVLLFTFLPK
>Haliclona_amboinensis|Hamb_m_255_glutamate
ALDRINRNSSILSNYTLNICSRTPSLGSGCSVATEPVASFASSAINLGNRLRFKNYFQLY
PVIQYYGWARQIACYNWIFYGWYDDEWFQTLYDATWTLALALGNVQFLVFTTCFRNKKII
RLTSPNLNYFIITGTAMVFVSGYTLAFGTILSKMWRIYYIFHNPTDYHLYLGALQIIALI
FAIQTRKVRIKILNDSKEVAAIIYIMVVIFIPK
>Amphimedon_queenslandica|PAC1571582_glutamate
ALERINSNSSILPNFTLSYCLRTPSLGSGCSVATDPVASFASSSVHLRDRDRFKNYFQIY
AIMTFYGWARQVICYGWILYGWYSNAWFYELYDATWTLALALGNVSFLVFTFYFRNKKLI
KLISPNLNYFIIAGTSMVFLSGYTIAFGTILSKMWRIYYIFHNPTDWHLYLGMLQIIALI
LAIQTRKVRIKILNDSKEVATIIYI--------
>Amphimedon_queenslandica|PAC1572665_glutamate

ALEKINSNSSLLANFTLRYCRRTPSIGSGCSVATDPVASFSSSSVHLRDRDRFRNYFQIY
ALITYYGWARQVICYAWILYGWYNNEWFYELYDATWTLALALGNVSFLIFTFYFRNKKLI
KLISPNLNYFIIAGTSMVFLSGYTIAFGTILSKMWRIYYIFYNPTDWHLYLGMLQIIALI
LAIQTRKVCIKILNDSKEVAVIIYILLVIFAPK
>Amphimedon_queenslandica|PAC1571583_glutamate
ALERINSNSSLLSNFTLSYCLRTPSLGSGCSVATDPVASYSSSSVHLRDRNRFKNYFQIY
VIMTHYEWARQVICYAWILYGWYSNQWFYALYDATWSLALALGNVSFLIFTFYFRNKKLI
KLISPNLNYFIIAGTSMVFLSGYTIAFGTILSKMWRIYYIFHNP-----YLGMLQIIAII
LAMQTRKVRIKILNDSKEVATIIYILLVIFAPK
>Haliclona_amboinensis|Hamb_m_306_glutamate
AVTD--FR-HLLSGFTIDICDASRTLGGGCSPTTDVLVSYTPSPARQ-----SANYFQLY
PILMEYGWARDILCYAWLLYGWYSNNW-----DAVVALMTALGDVYFLLFNIIFRKKKII
RLTSPNLNYFIIGGALLLFSIGIVSCFAPILTKMFRIYRIFRNPTDWHLYIGILETIAMV
LAILTRQVKVEAVNDSKEVVAVVYIITLMFIPK
>Amphimedon_queenslandica|PAC1572446_glutamate
AIAA--FSNELLPQYSLNICNASRGLGGGCPSTTSPLLSYNPGLMRY-----SQNYVQVF
PVISEYGWARRVLCYAWLIYDWYNEDW-----DAMVSLMRGLGDVSFLIFNIIFRNRKII
RLTSPNLNYFIIAGAIILYSLGITSIFAPMLAKMFRIYRIFRNPTDWHLYILILEAIAVV
LAAKTRGVKVEAVNDSKEVVAIVYIVTLMFLPK
>Haliclona_tubifera|Htub_mm_23_glutamate
AVDMINNDSVLLRGYRLTHCERRSAYGAGCSTSSEPVAAYVSASPLLSDRVRYPNFYRTY
ILVKSFGWARDVLCYAFITHAWFQEEWFDRLYDGVYSLAIAMGPVMFLIFNIVFRNKRII
KLSSPTLNYVIVAGSLLSYLSGYTLSFTAVLAKMWRVYYIFHNPKDWHLYAFVLQLVAII
LAFQTRKVKIKGLNDARQVIAIIYILTLLFIPK
>Petrosia_ficiformis|Pfic_m_155_glutamate

--LVFTIVFRNKKIV
KLTSPNLNYIVIVGALFMYGSGYTLSFSAVLAKMWRVYYIFHNPTDWHLYLAILQVIAIV
LAFQTRKVKIKALNDAKQVTAIIYALGLIFIPK
>Haliclona_amboinensis|Hamb_m_363_glutamate
ALEKINNHPSLLRGYNLSYCDRGKALAAGCSVATQPVASYASSAPELSIRSQFPSFFRTY
PLIRGFGWGRAIICYAFITHGWYQDRWFDAEYDALWSLGLALGYVEFIAFNFIFRNKKII
KLTSPNVNYVIAIGGLCIYAAGYTIAFSAVLAKMWRVYYIFHNPKDWHLFLFILQVIGIV
LAFQTRKVKVKVLNDAKQITAIIYILLLIFVPK
>Amphimedon_queenslandica|PAC1572463_glutamate
ALELINRNTSILPGYELSYCDHRQGLAAGCSAATLPVASYSSAAAQLSDRTTFPSFFRTY
PLVRGLGWAIAILCYAFITFGWYQTRWFDARYDALWSLALGLGYVEFLAFNVYFRNKKII
KLTSPNLNYTIGAGSLVMYSSGYIIAFSAVLAKMWRVYYIFHNPKDWHLMLFLLQAIGIF
LAFQTRKVKVKVLNDAKQVTAIIYVLILIFIPK
>Amphimedon_queenslandica|PAC1572463_glutamate
ALELINNNTSILSGYELSYCSHRQGRGAGCSAATLPVASYSSAAPMLSDVSLYPSFFRTY
PLVRGLGWARAAFCYAFIIPGWYQTGWFDTRYDALWSLALGL-----LAFNVYFRNKKII
KLTSPNLNYTIGAGSLVMYSSGYVIAFSAVLAKMWRVYYIFHNPKDRHLYLFLLQVIGIF
LAFQTRKVKVKILNEAKQVTAIIYVLVLIFIPK
>Petrosia_ficiformis|Pfic_m_153_secretin/adhesion
HCRRC------VVCAGFILRLLVSAVWSAWGMACLLRHCFQVSS-----QSSILTS-PLV
SVKQSED-WSETVRFNECCHYINVYG--------TAGMMVGFLSRHHCWKEACTHVLKST
DVFVNHLWIPGLKSGQLEYLEECLRSFSGRS-----------------------------
--------------TTIEELY

Secretin/adhesion GPCRs

>Amphimedon_queenslandica|PAC1571736_secretin/adhesion
ATRNCWERPNIQPCSEKMLTPVLDNEWSSDGCSLNICSCDHLTSFAILLDVAVFQYLTYI
GLTVSIICLIVTLLIHLSFALLGLYFTFIIACGIIGALVHYFFLAVFFIMAAESVDLFLV
IVLGPKIQRFILKTAIIGWIAPLFVIILLPFSVIYIFNVSVLFGLGWGFGFNSIFTIFTV

FQGFFIFLLYVVLSPNARNIW
>Haliclona_amboinensis|Hamb_m_412_secretin/adhesion
ATRNCWLRPNIAACSSRMQTPVIDEDWSAEGCRLNTCSCNHLTSFAILLDVSAFEYITYI
GLTLSTVSLVVTLLINLCFALLGLYITFLLACGIIGAMVHYFFLAVFFIMAAESVDLFLV
IVLGPKIQRFILKTIIIAWIAPLFFVMLLPFCIIYIFNVSVLFGLGWGFGFNSIFTVLTV
FQGFFIFLLYVVFSPNARKVW
>Haliclona_tubifera|Htub_m_351_secretin/adhesion
ARRTCWNPPMDSMCQTRMLSSVFDGGWSREGVSTVICSTDHLTSFSVLLDVAAFTALTYI
GISVSIVCILITLLINLCFALLGLYLTFFLACAVVGALLHYFLLVVFFIMAAEAIDLFLV
IVLGSTIQHYLTKAVIIAWILPLFIVVLFPFLVVYIFNISILFGLGWGFGFNAMFSILVC
FQGFFIFLLYIVLSPNAKKEW
>Amphimedon_queenslandica|PAC1571284_secretin/adhesion
ATRRCWEPPIDEACSKRLLSILISGNWSNYGCSVVICGCNHLTTFALLLDMLFVSSVTFL
GCAVSILCLVVIILINMSCALVGLYVSFIPACVVVGAFLHYFFLATFLSMASEATILYLV
KVFAKSKHGMIIKAVIVTWVTPFFIVFISPFVMIYLYNLSILFGLAWGIGLELSFVIAAG
LQGILIFIFYGLRISKVRKTW
>Haliclona_amboinensis|Hamb_m_428_secretin/adhesion
ATRRCWEEAKDSQCSGRLLSVLISGNWSMYGCQVVVCGCNHLTTFVLLLDVSVLMSFSYI
GCTVSAVCLVIIILVNMSLALLGVYVVFIPACVVISALLHYFFLASFVAMAAEATILYLV
KVFSKQKNSMILKAVIVTWVLPLFIVYIAPFCIIYIYNLSFLFGLGWGIGLQIIFVIIVG
AQGLFIFVFYGIRVTKVRKVW
>Haliclona_tubifera|Htub_mm_83_secretin/adhesion
AHRQCWQEPYDDECSRRILSSIISGNWSSKGCYTVTCACDHLTTFALIMDVAALSAFSLI
GNIVSIFSLSLIILLSLCSALLGLNFVFILACVINGAILHYFILTCFLAMASEATVLYLV
QVFAAGKENLPIKVALVTWITPVFIVSIIPFGVIYTYNLSLLFGLGWGLGFEILFIVLTG
FQGLFIFILYGVRLRKIRLLW
>Amphimedon_queenslandica|PAC1571284_secretin/adhesion
ARRYCWGMGHFSPCPSRLLTSVISLDWSNHGCHLIVCLCDHLTNFAALLDVSALNALSYI
GNIVSLFCLGITILLNFCLALVGLYLSFILSCAIASILLQYFLLVSFCAMATEALNLYLV
IVLGKGISRYVLKAALFTWVTPVFVVTVAPFILIYLFNLSILFGLGWGFGFSVIFILLTS
FQGLFIFIMHCLRSAEVRKEW
>Amphimedon_queenslandica|PAC1572315_secretin/adhesion
ASHKCWEDLKDEKCTTKLMSVIFSGDWSTEGCIVVLCHCNHLTSFAIILDLTTLSAITYI
GTIVSTVCLLITILIHLSVALIGLYGIFIGSCSMTAILLQYFFLVIFMLMAALAVDLYLV
VVLGGKISYYVLKATIVSWVLPIFITMIAPFAVIYLFNLSVLLGLGWGMGFEVLFVSLTS
FQGLAVFVMQCLRSLEARTIW
>Haliclona_tubifera|Htub_mm_84_secretin/adhesion
ARRYCWMEGFVAPCSARLLSEIFSGDWSQSGCESLTCRCDHLTNFAVLLDASALTAVSYI
GVILSIVCLLVVILLSLCFALLGLYVSFILACAIISIILQYFMLVTFLVMASEAINLYLV
IVLGKGISRYFLKATLIAWITPVFIVIIAPFIIVYIFNLSLLFGLGWGVGFSALFILLTA
FQGLFLFIMHCLRSKEVRKQW
>Haliclona_tubifera|Htub_m_433_secretin/adhesion
-------------------SIIISGNWSGIGCHAVVCLCNHLTSFSILLDVSALNAITYI
GIIVSLFCLILLCLLNLCFALIGLYITFISACAFVSAMLQYFFLATFIAMAAEAINIYLV
VVLGSKISHYVLKIVIINWSVPFFIVVVVPFILLYIFNLSVLFGIGWGIGLSALFILITA
FHGVFIFIMNGIRSIDVRSVW
>Haliclona_tubifera|Htub_m_433_secretin/adhesion
ATRYCWSNAIVDQCSAKILSIIISGNWSGSGCHAVVCLCNHLTSFSILLDVSALNAITYI
GIILSLFCLILLCLLNLCFALIGLYITFIFACAFVSAMLQYFFLATFIAMAAEAINIYLV
VVLGSKISHYVLKIVIINWSVPFFIVVVVPFILLYIFNLSVLFGIGWGIGFSALFILITA
FHGVFIFIMNGVRSKDVRSIW
>Haliclona_amboinensis|Hamb_m_398_secretin/adhesion
ATRHCWMAPYDVRCSTKLLTIILAGDWSTVGCMISLCHCTHLTSFCVILDVSALSAVTYI
GVIISIFCLIITILLNLCYAFIGLYFVFILACAIVAVVLQYFFLVTFMLMGAEAINLYLV
IVLGGKIEHYVLKAALISWITPIFIVMITPFIIIYVFNLSILFGLGWGVGFAAFFVLLTS
FQGLFIFIMHCLRSPEIRKVW
>Petrosia_ficiformis|Pfic_m_181_secretin/adhesion

------------------------GDWSSEGCVVVTCNCNHLTSFSMLLDVSALSAVTYI
GCIISILCLLLTILINLCISLIGLYTFFIVACAVVAAILQYFFLTTFMLMAAEAINLYLV
IVLGANIPHFILKVTIVCWVVPIFIVMLVPFIIIYMFNLSILFGLGWGIGFAALFVCLTT
FQGLFIFIMHCLRSPEIRKEW
>Amphimedon_queenslandica|PAC1571676_secretin/adhesion
ATRYCWDAPDVMRCSSRIL------------------------------DVSFLDSISYV
GIMVSIVCLIITILLNLCFALLGLYLSFIVACAFSGAVLQYFFLVTFIVTAAEAIDLYLV
IVLGHKIDHFVLKATLVSWIAPVFVVVVVPFVLIYLFNLSILFGFGWGLGIASVFVFLTG
FHGLFIFVIYCLRSKEVRFVW
>Amphimedon_queenslandica|PAC1572825_secretin/adhesion
ATRYCWDAPDVMRCSSRILSIIISGDWSNDGCNTVTCHCDHLTNFAVLLDVSFLDSISYV
GIMVSIVCLIITILLNLCFALLGLYLSFIVACAFSGAVLQYFFLVTFIVMAAEAIHLYLV
IVLGRKIENYVLKATVVSWIAPVFVVMVVPFVIIYLFNLSILFGLGWGLGIASVFVILTG
FHGLFLLITYCLRSEEARHVW
>Haliclona_tubifera|Htub_mm_88_secretin/adhesion
ATRKCWEFPDVSACSAKLLSIIVSGDWSTEGCTVTLCLCTHLTSFSMLLDVSALEVVTYI
GCIVSIVCLVVTILLNLSFALIGLYGTFILACSFVGAVLQYFFLATFLLMGAEAINLYLV
VVLGSNIQQFILKVAVFCWVLPIFIVVVVPFILIYIFNLSILFGLGWGIGFASLFVLLTS
FQGLFIFFMHCIRSPDIRKQW
>Haliclona_amboinensis|Hamb_m_441_secretin/adhesion
ATRYCWSSPDVSQCSARLLSIIISGDWSSEGCNTVMCQCDHLTSFAILLDASFLDSVTYI
GIVVSLICLTITVLLNLCFALMGLYISFIIACVFIASVLQYFFLVTFAVMAAEAVNLYLV
IVLGRKIHNFVTKAIILSWIVPLFIVMLVPFLIIYLFNLSVLFGLGWGIGLAALFILATA
FHGLFIFIVHCLRSKDARNVW
>Petrosia_ficiformis|Pfic_m_200_secretin/adhesion
--------------SAKILGIIISGDWSSDGCKIVTCFCNHLTSFSILLDVSALNAVTYI
GIIISIFCLLITILINLCLALIGLYITFIFACAFVAAALQYFFLVSFMIMASEAISLYLV
VVLGRNIPHFVMKATLISWVTPIFIVMVVPFLIIYIFN---------------------

>Haliclona_tubifera|Htub_m_263_secretin/adhesion
ATRRCWDT---VYCGSQLLSSVISGRWNSSGCLTVTCSCNHLTNFAILLAPNILGIIGLV
LIPISILCMIIIIHIMMCVSLCVAQIVFVAGCSVVAVILQYLFLVCFMWMLMEGVVLYLI
KVIIKNPKCYGFVFTISCYAIPAMYMFIAPVITILLINLTVVMGLTWLVGMSYLTTVFIA
GQGILLFIILVPLSKQVRDAY
>Amphimedon_queenslandica|PAC1571565_secretin/adhesion
ANRFCWEE---IIC-----------------------------------IRMKIAGTRLLL
------------INNSVILSETITQLTFLFGCSIIATVMQYMFLVTFMWMLMEGAILYLV
KVFTTRTRHYAVAFTLVCYSVPAIYMFIAPVILIIILINLTCVMGIAWLGSIAYIMTVLIA
GQGIVIFVLYVPLSKHVRAAY
>Haliclona_amboinensis|Hamb_mm_28_secretin/adhesion

-------------------------------CSAIAIVLQYMFLVTFMWMLMEGVVLYLV
KVFVTHTKRYTLAFTIFSYGVPALYMFIAPVILIILANLTIVMGIGWLGNVAYVMTVFIA
CQGIIIFVLYVPFSKQVREVY
>Petrosia_ficiformis|Pfic_m_127_secretin/adhesion

--------------------------------------LTLVMGIAWLANVAYIMTIFIA
GQGILFFIILVPLSKHWREAY
>Amphimedon_queenslandica|PAC1572242_secretin/adhesion
ATRLCWGLPNVTNCSNVIVTAVTGGNWTLAGCTTVTCVCNHLTNFACLVDIGALNIFSII
GVFVSLLGLLLTIHIQLCLSLIFMLIVFVAGCVTVGVLIHYFALVSWMWMGAEAVMMFLV
IVFSNITWKYILFVSIICWAVPLLPLFLIPVFLILLFNVMLLFGLTWIFGVH--------
--------------SESRDAW
>Haliclona_amboinensis|Hamb_mm_57_secretin/adhesion
ATRRCWDSPIITSCSNQIVSNVISGNWSTDGCSTVNCSCDHLTNFACLVDISALQAVSIV
GVCLSLVGLIITIHIQLCIALCCMLIVFVSGCITVGVLIHYFTLVAWMWMGAEAVLMFLV

IVFSNITWRYLLAVSILCWAAPLLPVFLIPILLVIIFNILFLFGVTWVFAIQFFFAFFNA
FQGFFIFLFFVMLSSDSREAW
>Amphimedon_queenslandica|PAC1570592_secretin/adhesion
AIRQCWDTHDISNCSNNII---------------------HLTNFACLVDISTLEIVSYI
GVCLSLLGLVLTIHIQLCLSLIFMLIVFVVGCITVGVLIHYFALVSWMWMGAEAVLMFLV
IVFTNLTWKYFLIVSITCWGLPLLPVFFVPVMIILILNIMLLFGLTWIFAVQFFFAFFNA
FQGFFIFVFFIVLSNDSRTAW
>Amphimedon_queenslandica|PAC1571483_secretin/adhesion
ATRECWGSPNISSCSNNIVSSVIS----------GTMVPPHTVTVALEIVSSALEIVSYV
GVSLSLLGLVLTIHIQLCLSLIFMLIVFVVGCITVGVLIHYFALVSWMWMGAEAVLMFIV
VVFSNITIKYLLVLSVICWTLPLLPIFLVPVFLILIFNIMFLLGLTWIFGLQFLFAFFNA
FQGFFIFIFFVVLSSDARAAW
>Haliclona_tubifera|Htub_m_398_secretin/adhesion
STRSCWSEPIIDLCSPTIVSSVVS-DWDSSGCVTVICLCSHLTNFACLVDVYALEAVSYV
GVTLSLIGLVLTFHLQLCVSLACMYIVFVSGCVTVGTMIQYFTLVAWMWMGAEALLMFLI
IVFGTITWKYLLLVSVVCWSVPIIPVFLGPIFLVIIFNIMMILGLAWVFAFQLIFSLLNA
FQGFWIFFFFFVLLNAEARNPW
>Haliclona_amboinensis|Hamb_m_432_secretin/adhesion
ASRSCWGSSDTSACVNSVISQVVSGNWSTAGCRTVLCQCDHLTNFACLVDISTLEVISYV
GVIVSLLGLLLTGHIQFCIALFMMLLVFVSGCIAVGVFIHYFALATWMWMAAEALLMFIV
IVFSNITWRYHLIVSSVCWALPLLPVFLTPMFLLLIFNIMFLFGLSWLFLAQLMFTLLNV
FQGFFVFIFFVVLNADCRDAW
>Amphimedon_queenslandica|PAC1571202_secretin/adhesion
ATRYCWSVSDTSNCVNIIISQVIGGNWSTSGCRTVRCECNHLTNFACLVDISTLESLTII
GVIFSLVGLTLTAHVQLCISLFLMLLIFVSGCITVGIFIHYFALVSWMWMGAEALLMFLV
IVFSNITWRFHLVVSLICWGVPLLPVFFVPILLLIIFNIMSLFGISWIFAAQIMFTLFNV
FQGFFIFIFFVVLNNDTREAW
>Amphimedon_queenslandica|PAC1572642_secretin/adhesion
ANRACWESPNVTLCSNNVISSVIGGNWSTDGCNTINCHCDHLTNFACLVDVSTLEVVSIY
GVCLSLVGLILTIHIQLCLSLIYMLIIFVVGCITVGVLIHYFALVSWMWMGAEALLMFLV
IVFTDITQKYLIAVSILCWTLPLLPVFLLPVFIILIFNSGSGFGGG--------------
-SGANPF------GDDDK---
>Amphimedon_queenslandica|PAC1571984_secretin/adhesion
--------------QGDIISSVIGGNWSTDGCNTINCHCDHLTNFACLVDVDILKVVSIV
GVCFSLVGLLLTIHIQLCLSIIFGSIVFIVGCITAGVLMHYFSLVSYIWMGAEALLMFLV
IVFTDITWKYLTAVSILCWTLPLLPVFLLPIFIILIFNVLILFGLTWFIAIRLIFAFFNG
FEGFFIFFFLVILSSDSRNAW
>Amphimedon_queenslandica|PAC1570171_secretin/adhesion
------------------------GNWSTDGCNTIACHCDHLTNFACLVDVSTLEVVSIV
GVCLSIGGLVLTIHIQLCLSLIFMLAVFVSGCITVGVLIHYFALVAWMWMGAEALLMFLV
IVFTDITWKYLVGISLICWTLPLLPVFLLPVFIILIFNILILFGLTWVSAVQFFFAFFNA
FQGFFIFFFFIILSSDSRDAW
>Amphimedon_queenslandica|PAC1572493_secretin/adhesion
ASRKCWLWPRTSNCSTLLLSNVWSGQWMTDGVYTVVCSSYHLTAFAVLVSVNAMSIATYA
GTAASVLCLALSFHLNLIIALMLAYSIFGLGCSVVAGLLHYFFLASFCWMLCEGIMLYLV
VVFSRLAKRWYFFF-AIGWVTPLIPVFVAPMLLIIAINLLPLMGFTWLFGFAWLFNILNS
TQGVAIFFLYVVRNDKVW-SK
>Petrosia_ficiformis|Pfic_m_190_secretin/adhesion
------------------------SQFSTEGVITVDCQSDHLTPFAVLVDVSALKAVSYI
GLAISMACLLLTIHLNLAISLFCGYLVFATGCGFVAALLHYFFLASFCWMLCEGIMLYLV
VVFSSFAKRWYLFL-LLGWVPPLLPVFVAPMIIIILINLLPLLGFTWLFGFAWLFTLFNI
FQGMAIFFFHVVRSEKVW-TK
>Amphimedon_queenslandica|PAC1572446_secretin/adhesion
ATKACWGETNVLDCSTELLSIVWSREWSIDGVNTVYCQSFHLTAFAVLVDVSALKVVSYI
GCAISIACLIVTVHLNLALSLLCGYFIFAVGCAFVAALLQYFFLSSFCWMLCEGVMLYLV
KVFSTLSKRWYFFL-ILGWAPPLLPVFVAPMIVIIIINLLPLLGLTWLFGFAWIFTLLNT
LQGAAIFFFHVVRSDKVW-SK

>Haliclona_amboinensis|Hamb_m_444_secretin/adhesion
ATRACWDEPDVIDCSDILLSLVWSGEWSDNGVVTVKCQSFHLTAFAVLVDVSALKVVSYI
GCAISLVCLILTIHLNLAISLFSGYFIFSVGCAFVAALLQYFFLSSFCWMLCEGVMLYLV
KVFSSLSKRWYFFL-ILGWVPALLPVFVVPMLIIIIINLLPLLGLTWLFGFAWIFTLLNS
LQGAAIFILHVVRSDKVW-LK
>Amphimedon_queenslandica|PAC1572835_secretin/adhesion
QDNFC--GDNQLRCSTALLSNVINNVWSDVGIETILCNTSHLTSFAVLVDHQALSIVGYI
GPSISLLCLIIALHLNLCISLLLGLTVFLFGCSIVAVLLHYLFLCVFAWMLAEGITLFVV
HVWGMKLLKWQISL-LTAWGIPLFIVFLGPALAIAVINLFPLLGITWIIGFLWLFTICNS
LQGVFILIFHVLRHKTVL-TW
>Amphimedon_queenslandica|PAC1572835_secretin/adhesion
MNRLC--GDERLRCGAMLLSLVLSNMWSDAGIETVKCITSHLTSFAVLVDHQALSIVGYI
GPSISLVCLIVALHINLCISLVLALSVLIVGCSIVAGLLHYLFLCVFAWMLAEGITLCVT
YVFEMKFLKWQIFL-PAAWGIPVLIVFLGPALGVALVNLFPLLGITWTIGFLWLFTICNS
LQGVFILIFHVLKHKVVL-TW
>Haliclona_amboinensis|Hamb_m_377_secretin/adhesion
LERLC--GDERLRCGAMLLSLILSMEWSANGVETVRCTTNHLTSFAVLVDHRALSIVGYI
GPSFSAVCLIIAIHLNLCISLLMALAVLLVGCSVVAGVLHYLFLCVFAWMLVEGITLFVV
YVWGIKFLKWYIFV-PLAWGIPVFVVFLGPVALICVINLFPLLGITWVIGFLWLFTICNS
LQGVFILVFHVLKHKAFL-TW
>Amphimedon_queenslandica|PAC1572221_secretin/adhesion
ARRRCWKLPNVSNCSEELLSVVLSSRWESEGID-VRCLSSHLTNFAVLVSVVLLSVFSYI
GCSVSIMCLLATIHLNLSIALLLALILFVGGCKTIAVLAHYFFLSAFSWMLCEAIMLYLV
VVFSRLIKMWYIFL-ILGWGLPIIPVFVGPILA---INLLPLLGSTWIIGFAWLFVIFNS
LQ--------------VW-GR
>Haliclona_amboinensis|Hamb_mm_31_secretin/adhesion
ARRWCWARPDLARCSEELLSVILSGGWTADGIVTIQCYSYHLTSFAVLVTVVLLSIVSYI
GCSISILCLLVTVHFNLSIALLVALILFVAGCTTITVLMHYFFLSAFSWMFCEALMLYIV
VVFSRITDKWWLYL-LLGWGLPLIPVFAGPIIGVICINLLPLLGSTWIIGFAWLFVILNS
LQGAFIFFFHVFRGPLIW-GR
>Haliclona_tubifera|Htub_mm_52_secretin/adhesion
IRRFCWAKPDISMCSEELLSAIISGGWVSEGIEVIKCLSKHLTSFSVLVDISLLSVVSYI
GVSVSIVCLSVTVHFGLAVSLLIALILFIGGCKLVAALLHYFFLSAFCWMLCEAIMLFLV
LVFSTLSKKWWLFC-IIGWGFPLVPIFVGPMILIILVNLLPLLGLTWIIGFAWIFTIFNS
LQGVFIFVFHVLRSKKFT-EK
>Petrosia_ficiformis|Pfic_m_193_secretin/adhesion
--------------GPELLTRIISGFWSSEGITTVICLSNHLTSFAVLVDTAALSIVSYI
LLTISVVCLILTVHLNLALALLIAFCVFLFGCTFIAALLHYFFLSVFCWMLCEGVMLYLV
VVFSTLSKKWWFFL-ILGWVPPIIPVFTVPMIIIIIINLLPLLGLTWLFGLAWLFVILNA
SQGIAIFILHVVRNERIW-NR
>Haliclona_tubifera|Htub_mm_94_secretin/adhesion
TSRMCWLPVNISQCSEELLSVILSGSWLSDGIVTVRCLSSHLTSFAVLVDVSALSYVSYI
GTGLSVVCLVLTIHLNLAISLLLGLIVFIAGCKIVAILLHYFFLSAFCWMLCEAILLYIV
IVFTSPAKFWYLFV-LFGYGVPAVIVFVGPMCLIISVNLLPLLGVTWVVGFAWLFTVLNS
LQGVFIFVFHVLCSERVT-NR
>Petrosia_ficiformis|Pfic_m_932_secretin/adhesion
-------------------SLMISMKWSDEGVETVECVTNHLTSFAVLVDHRALTVIGYI
GPAISIVCLIISIHFNLCIALLLGLITLVAGCGLIAGLLHYLFLCVFCWMLSEGIMLYVI
HVWGSISKKWYIFL-PLGWGLPLIIVFLGPVLVICLINLSPLLGVTWIIGFLWIFTITNS
LQGVAILFFHVLHNKVIW-SR
>Amphimedon_queenslandica|PAC1572489_secretin/adhesion
CEN-G----SNPSCGPNLLSLVVSQNWSTDGMTT------------------GLEIISYI
GVSISIICLILSIHINLSLALLLALLVFVFGCSVVAGLLHYLFLCVFCWSLAEGIMLYIV
RVYGSLADRWYLLL-PLGWGLPIIIVFIGPMLLIIIINLLPLLSITWIIGFQWLFTIFNS
IQGCAVLYFHVLRNKFIT-NW
>Haliclona_amboinensis|Hamb_m_329_secretin/adhesion
CNAIP----DTDECSPQLLSLIVSNVWSTDGVVTVVCISNHLTSFAVLVDSTALNTISYI

GVIISILCLILTIHINLSVALLLALILFVAGCSVVAGLLHYFFLCVFCWSLGEGIMLYLV
KVYGSLSDRWYLLL-PLGWGLPVIVVFVGPMLLIIAINLLPLLGITWIIGFLWIFTICNS
LQGVAILYFHVLRNKFVN-NW
>Amphimedon_queenslandica|PAC1571439_secretin/adhesion
------------------------------MTTVHCTSNHLTSFAVLVDHSALSIIGYA
GPVISIIALIITLHVNLSLSLLLALLVFVLGCSVVAGLLHYLFLCVFCWSLAEGIMLYLI
KVYGSLADKWYLLL-PLGWGVPIIIVFVGPILLIILVG----------------------

>Amphimedon_queenslandica|PAC1572493_secretin/adhesion
CL--N----RTKDCGPMLL-----QNWSTDGMTTVLCTSNHLTSFAVLVDHSGFQVVGYI
GCIISIIALLFAIHINLSLALLLALLVFVFGCSVLAGSLHYLFLCVFCWSLAEGIMLYIV
RVYGSLADRWYLLL-PLGWGLPIIIVFIGPMLFIIIINLSPLLGITWVIGFLWLFTILNS
LQ--------------IS-DW

Rhodopsin GPCRs

>Amphimedon_queenslandica|PAC1571077_rhodopsin
EAVAGVLSIEMILALIANGVVLVITIYKSWKQSSTIFFTSLILAHLVL-TLYLPFSIAWI
IGETDEDKEGTCHFTLCLISIDRFLFIVKPHLHVALVLVIIVWIVNA-----VWTFCFTR
KFINNQSVYASKKKRLFGIFGSMLLVYGICFIPFFLAVVLSPVVQAYFRPEI
>Amphimedon_queenslandica|PAC1571077_rhodopsin
EAVAGVLSIEMVLALIANGVVLVVTIYKSWKQSSTIFFTSLILAHLVL-TLSLPFSIAWV
VGDTDEEKQGTCGFTLSLISIDRFLFIVKPHLHVALVLVIIVWIVPAF------------
---ASLSVYASKKKRLF---GSMLLVYGICFTPFFFAVIPSPVIQAYFRPEI
>Amphimedon_queenslandica|PAC1571077_rhodopsin
EAVAGVLSIEMILALIANGVVLVITIYKSWKQSSTIFFTSLILAHLVL-TLYLPFFIAWI
IGSTDGEKKATCDFTLSLISIDRFLFIVKPHLHVALVLVIIVWIVTAIVVTSIWTFCFTR
MFMNNQSVYASKKKRLFGIFGSMMVVYCIFFTPFFLVVVLNPVVQAYFRPEI
>Amphimedon_queenslandica|PAC1572286_rhodopsin
EAVAAVLSIEMILALIANGVVLVITIYKSWKQSSTIFFTSLILAHLVL-TLYLPFTISWI
IGSTDEEKQGTCGFTLCLVSIDRFLFIVKPHLHIALVLVIIVWSFDTIIITSVWTFCFTR
KFINNQSVYASKKKRLFGIFGSMMLVYGIFFTPFFLVITLSPVIQAYFRPEI
>Amphimedon_queenslandica|PAC1570218_rhodopsin
EAVAGVLSIEMILALIANGVVLVITIYKSWKQSSTIFFTSLILAHLAL-TLYLPFSIAWV
VGDTDEEKQGTCGFTLSLISIDRFLFIVKPHLHVALVLVIIVWSFDLIIITSVWTFCFTR
KFINNQSVYASKKKRLFGIFGLMMLVYGICVSPFFLAVVLSPVIQAYFRPEI
>Amphimedon_queenslandica|PAC1571077_rhodopsin
EAVAGVLSIEMILALIANGVVLVITIYKSWKQSSTIFFTSLILAHLAL-TLYLPFTIAWV
VGSTDGEKQGTCSFTLSLISIDRFLSIVKPHLHVALVLVIIVWSFDLIIVTSVWTFCFTR
KFINNQSIYASKKKRLFGIFGSMLLVYGIFFTPFFLVVVLSPVVQAYFRPEI
>Amphimedon_queenslandica|PAC1571643_rhodopsin
QAVAGVLSIEMILALIANGVVLVITIYKSWKQSSTIFFTSLILAHLFM-ILYLPFTITWI
IGSTDEEKEGTCDFILSLISIDRFLFIVKPHLHVALVLVIIVWIVTAIIITSIWTFCFTR
KFINNQSVYASKKKRLFGIFGSMILVYGICFAPLFLVVTLSPVVQSYFRPEI
>Amphimedon_queenslandica|PAC1571643_rhodopsin
EAVAAVFSIEIILALIANGVVLVISIHKSWKESSTIFFTSLILAHLLM-LLVIPFSITWI
IGSTDEEKEGSCGFTLSLISIDRFLFIVKPHLHVALVLVIIVWIVTAILITSVWTFCFTR
KFINNQSVYATKKKRLFGIFGSMLLVYGMCFIPFLLGIILSPVVQSYFRPEI
>Amphimedon_queenslandica|PAC1571083_rhodopsin
EAVAAVLSIEMILALIANGVVLVITIYKSLKQPSTIFFTSLILGHLVM-ILYLPFSITWI
IGSTDEERQGSCDFTLSLISIDRFLFIVKPHLHVALVLVIIVWVFNAIVVTSVWTFCFTR
KFINDQSVYASRKKRLFGIFGSMLLIYGVCFIPFFLAIVLSPVVQSYFRPEI
>Amphimedon_queenslandica|PAC1571643_rhodopsin
EAVAAVLSIEMILALIANGVVLVITIYKSWKQSSTIFFTSLILAHLTL-TLYLPFSIAWI
IGDTDEEKKATCDFTLSLISIDRFLFIVKPHLHIALVLVNIVWIVNAIIITSIWTFCFTR
KFINNQSVYASKKKRLFGIFGSMILVYGICFAPFFLAIILSPVVQSYFRPEI

>Amphimedon_queenslandica|PAC1571643_rhodopsin
EAVAGVLSIEMILALIANGVVLVITIYKSWKQSSTIFFTSLILAHLVL-TLYLPFSIAWI
IGSTDEEKKGTCNFTLSLISIDRFLFIVKPHLHVALVLVIIVWIVNAIVVTSVCTFCFTR
KFINNQSVYASKKKRLFGIFGSMLLIYGICFAPFFLAIILSPVVQSYFRPEI
>Amphimedon_queenslandica|PAC1571643_rhodopsin
EAVAAVLSIEMILALIANGVVLVITIYKSWKQSSTILFTSLILAHLLI-ILVLPFCIAWI
IGSTDEERKGTCDVTLSLISIDRFLFIVKPHLHVALVLVIIVWIATAIIITSVWTFCFTR
KFINNQSVYASKKKRLFGIFGSMLLVYGICFAPYLLAFILSPVIQSYFRPEI
>Amphimedon_queenslandica|PAC1571643_rhodopsin
EAIAGVLSIEMILALIANGVVLVITIHKSWKQSSTIFFTSLVLGNLVMTTLYLPFTITWI
IGSTDEEKQGSCDFTLSLISIDRFLFIVKPHLHVALVLVIIVWIVNAILITSVWTFCFTR
KFINNQSVYASKKKRLFGIFGSMLLVYGICFIPFLLALILSPVVQSYFRPEI
>Amphimedon_queenslandica|PAC1571643_rhodopsin
EAVAGVLSIEMILALIANGVVLVITIYKSWKQSSTIFFTSLILGNLVMTMLYLPFSIAWI
IGSTDEEKQGTCGLTLSLISIDRFLFIVKPHLHVALVLVIIVWIVTAIVVTSVWTFCFTR
KFINNQSVYASKKKRLFGIFGSMLLVYGICYIPFLLALILSPIVQSYFRPEI
>Amphimedon_queenslandica|PAC1571642_rhodopsin
EAVAGVLSIEMILALIANGVVLVITIYKSWKQSSTIFFTSLIMGNLLMTMSYLPFSIAWI
IGSTDEEKQGTCDFTLSLISIDRFLFIVKPHLHVALVLVIIVWIVNPIVVTSVWTFCFTR
KFINNQSVYASKKKRLFGIFGSMLLVYGICYIPFLLALIVSPVVQSYFRPEI
>Amphimedon_queenslandica|PAC1571497_rhodopsin
IALAVVLTIEVILAAIANGAVLCITIYKHWRQPSTIFFTSLILAHLVMMLLYLPFTITWS
IGRTNEQRIGTCFFTLAAISFDRFLFIVKPHLHVAVSLAVAIWILSAIVVTCTWTFCFTR
KFLKDQSVYSAKKRRLFGIFGSMLLVYIICFTPYHFITIANPLVQSYFRPEI
>Amphimedon_queenslandica|PAC1571069_rhodopsin
PVLAAVFATEMILALIANGVVLLITITNSWKQSSTIFFTSLILAHLVLNLLYLPFTIIWI
FGSTDEEKRETCDFTLAAISFDRFLFIVKPHLHVALTLTLAIWILSAIVVTSVWTFCFTR
KFIHNQSVYASRKKRLFGIFGAMLIVYGLCFTPFIFVTVLSPIVQSYFRPEI
>Amphimedon_queenslandica|PAC1571069_rhodopsin
PVLAAVFATEMILALIANGAVLLITITNSWKQSSTIFFTSLILAHLVLTLLYLPFNIIWI
FGSTDEEKRGFCSFTLAAISFDRFLFIVKPHLHVALTLTLAIWILSAITVTSVWTFCFAR
KFIHNQSVYASRKKRLFGIFGAMLIVYGLCFTPFIFVTVLSPIVQSYFRPEI
>Amphimedon_queenslandica|PAC1570612_rhodopsin
PVLAAVFATEMILALIANGAVLLITITNSWKQSSTIFFTSLILAHLVPNLLYLPFTIIWI
FGSTDEEKRGFCSFTLAAISFDRFLFIVKPHLHVALTLTLAIWILSAITVTSVWTFCFTR
KFIHNQSVYASKKKRLFGIFGAMLIVYGLCFTPFIFVTVLSPIVQSYFRPEI
>Amphimedon_queenslandica|PAC1571093_rhodopsin
PAVAAVLTVEMILALIANGVVLSITLYKSWKQPSTIFFTSLILAHLVLNLLYLPFSIIWI
FGSTDEEKKTTCTFTLAAISFDRFLFIVKPHLHVALTLTIAIWILAAIIVTSLWTFCFAR
SYFKAQSVYASKKKRLFGVFGSMLLIYGMGIVPLFFITIASSIIQSYFRPEI
>Amphimedon_queenslandica|PAC1571093_rhodopsin
PAVAAVLTFEMILALIANGLVLSITLYKSLKQSSTIFFTSLILAHLVLNLLYLPFTIIWI
FGNTDEEKIATCTFTLAAISFDRFLFIVKPYLHVALTLTIAIWILSAIIVTSLWTFCFAG
SFFKDQSVYASKKKRLFGVFGSMLFIYGICIVPFLFITIASPIIQSYFRPEI
>Amphimedon_queenslandica|PAC1571633_rhodopsin
PAVAAVLTVEMILALIANGVVLSITLYKSLKQSSTIFFTSLILANLVLNLLCLPFNIIWI
FGSTDEEKTATCIFTLAAISFDRFLFIVKPHLHVALTLTIAIWILSA-IVTSLWTFCFAR
SFLKDQSVYASKKKRLFGIFGSMLLIYGICVVPFFFITIASPIIQSYFRPEI
>Amphimedon_queenslandica|PAC1571094_rhodopsin
PAVAAVLTVVMILALIANGVVLSITLYKSWKQSSTIFFTSLILAHLVLNLLYLPFTIIWI
FGSTDEEKTGTCIFTLAAISFDRFLFIVKPLLHVALTLTIAIWILAAIFVTSLWTFCFTK
SYFKAQSVYASKKKRLFGVFGSMLIVYGTSYLLFPIVTIASPIIQSYFRPEI
>Amphimedon_queenslandica|PAC1570484_rhodopsin
PAVAAVLTVEMILALIANGVVLSITLYKSWKQSSTIFFTSLILAHLVLNLLYLPFTIIWI
FGSTDEEKRGFCSFTLAAVSFDLFLFIVKPHLHVALTLVIAIWILSAIFVTSLWTFCFTR
SFFKAQSVYASKKKRLIGIFGSMLLIYGTCSLFSYFVTVASPIIQSYFRPEI
>Amphimedon_queenslandica|PAC1570484_rhodopsin

PAVAAVFTVEMILALIANGVVLSITLYKSWKQSSTIFFTSLILAHLVLNLLCLPFTIIWI
FGSTDEEKRWTCIFTLAAISFDRFLFIVKPHLHVALTLTIAIWTLSVIFITSLWTFCFTR
SFFKDQSVYASKKKRLFGIFGSMLLIYGTSYLFFCFVTIASPIIQSYFRPEI
>Amphimedon_queenslandica|PAC1571094_rhodopsin
PAVAAVHTVALILALIANGIVLSITLYKSWKQSSTIFFTSLILAHLVLNLLYLPFTIIWI
FGSTDEEKTGTCAFTLAAISFDRFLFIVKPHLHVALTLTIAIWILSAIFVTSLWTFCFTK
SYFKAQSVYASKKKRLFGVFGSMLLIYGTAYLFFFFVTIASPIIQSYFRPEI
>Amphimedon_queenslandica|PAC1571093_rhodopsin
PAVAAVLTVEMILALIANGVVLSITLYKSLKQSSTIFFTSLILAHLVSNLLNLPFAIIWI
FGSTDEEKRGTCIFTLAAISFDRFLFIIKPHLHVALTLTIAIWILSTIFVTSLWTFCFTR
SYFKDQSVYAFKKKRLIGVFGSMLLIYGISHLLYFFVTIASPIIQSYFRPEI
>Amphimedon_queenslandica|PAC1571093_rhodopsin
PAVAAVLTVEMILALIANGVVLSITLYKSWKQSSTIFFTSLILAHLVSNLLNLPFTIIWI
FGSTDEEKTGTCDFTLAALSFDRFLFIVKPHLHVALTLTIAIWILSAIFVTSLWTFCFTR
SYFKAQSVYASKKKRLFGVFGSMLLIYGISHLLYFFVTIASPIIQSYFRPEI
>Amphimedon_queenslandica|PAC1571633_rhodopsin
QAVAAVFTVEMILALIANGVVLSITLYKSWKQSSTIFFTSLILAHLVLNLLHLPFAVIWI
FGSTDEEKRGTCTFTLAAISFDRFLFIVKPHLHVALTLTIAIWILCAIFVTSLWTFCVTK
SYFKDQSVYASKKKRLFGIFGSMLIVYGTAYLIFCFVTIASPIIQSYFRPEI
>Amphimedon_queenslandica|PAC1570617_rhodopsin
PGVAAVLIVEMILALIANGVVLSITLYKSWKQSSTIFFTSLILAHLVLNLLYLPFAIIWI
FGSTDEEKRGTCTSTLAAISFDRFLFIVKPHLHVALTLTIAIWILSAIIVTSLWTFCFTR
KFFKAQSLYASKKKRLIGIFGSMLLIYGTVYLIFCFVTIASPIIQSYFRPEI
>Amphimedon_queenslandica|PAC1570617_rhodopsin
PAVAAVLTVVMILALIANGVVLSITLYKSWKQSSTIFFTSLILAHLVLNLLYLPFTIIWI
FGSTDEEKTATCYFTLAAISFDRFLFIVKPHLHVALTLTIAIWILSAIFVTSLWTFCFTR
SYFKAQSVYASKKKRLFGVFGSMLLIYGTVYFSYFMVTIASAIIQSYFRPEI
>Amphimedon_queenslandica|PAC1572758_rhodopsin
PVLATILIVQIILALIANGVVLSITFYKSLKLSSTIFFTSVILAHLVLNLLYLPFRIIWV
FGSTNEEKKVTCTFTVAAISFDRFLFIVKPQLHVALTLTIAIWILSAIFVTSLWTFCFTR
SFFKNQSVYASKKKRLFGVFGSMLLIYGICFVPFDFITVANPIIQSYFRPEI
>Amphimedon_queenslandica|PAC1572230_rhodopsin
PAVAAVLTVEMILALIANGVVLSITLYKSLKQPSTVFFTSLILAHLVLNLLYLPFYIIWI
FNGSDEEKRGTCYFTLAAISFDRFLFIVKPHLHVALTLTIAIWILSAIFVFSIWTFCFTR
RFFRDQSAYASKKKRLFGIFGSMLLIYGICLVPFLFFTIANPVIQSYFRPEI
>Amphimedon_queenslandica|PAC1571094_rhodopsin
PAVAAVLTVEMILALIANGVVLSITLYKSWKQSSTIFFTSLILAHLVLNLLYLPFTIIWI
FGSTDEQKTGTCYFMLTTISFDRFLFIVKPHLHVALTLTIIVWLLAAMFVTSLWTFCFTK
SYFKAQSVYASKKKRLFGVFGSMLIIYGICFVPYLFITVANPVIQSYFRPEI
>Amphimedon_queenslandica|PAC1572230_rhodopsin
PAVAAVLTVEMILALIANGVVLSITLCKSLKQSSTIFFTSLILAHLVLNLLYLPFRIIWI
FGSTDEEKRGTCRFTLAAISFDRFLFIVKPHLHVALTLTVAIWILSAIFITSLWTFCFTR
RFFKDQSVYASKEKRLFGIFGCMLLVYAICFVPFLFITVASPLIQSYFRPDI
>Amphimedon_queenslandica|PAC1572230_rhodopsin
PALAGILIVEMILALIANGVVLSITLYKSLKQPSTMFFTSLILAHLVMNLLNLPFIIIWI
FGSTDEEKRGTCYFTLAAISFDRFLFIVKPHLHVALTLTIAIWILSAIFVTSLWTFCFTR
RFFKDQSVYISKKKRLFGIFGSMLLIYGICFVPFHFITVINPVMQSYFRPEI
>Amphimedon_queenslandica|PAC1572230_rhodopsin
PAVAAVLTVEMILALIANGVVLSITLYKSWKQSSTIFFTSLILAHLVLNLLYLPFIIIWI
FGSTDEEKRGTCYFTLAAISFDRFLFIVKPHLHVALILTIAIWILSAIFITSLWTFCFTR
RFFKDQSVYASKKKRLFGIFGAMLLIYGICFVPFLFITIASPVIQSYFRPEI
>Amphimedon_queenslandica|PAC1571674_rhodopsin
PTYAAALAIEAVAGLVANFTVLAITLYNSFKQSSTIFFTSLLLANLIMVIYILI-TSIWI
VGSTHEEKTATCAFTIAAISFDRFLFIVKPHLHVALFVTIGIWLLSGIFITSLWTFCFTR
RFMQSQSVYVSKRKRLFGIFGTMLLAYAIALSPYNSITIINPIIQSYFRPEI
>Amphimedon_queenslandica|PAC1572824_rhodopsin
PTYAAALGIEGVIGIIANVAVLLMTLYKSWNQPSTIFFTSLLLSSLIIALVYLM-SSFWI

FGNTFEEKNGSCIFTLAVISFDRFLFIVKPHLHVALILIIGVWLLCSIASTSIWTFFFTR
SFIKRQGVYQSKNKKLFGIFGSMLLSYILTLLPYGLISIMNPLIQSYFRPEI
>Amphimedon_queenslandica|PAC1570300_rhodopsin
PTYAAVLGIEGVIGIIVNVAVLLMTLYKSWNQSSTIFFTSLLLSNLIIALWYLI-SSIWI
FGNTFEEKNATCMLTLAAISFDRFLFIVKPYLHVALILITGVWLLCSIAFTSIWTFLFTR
SFIKRQGVYQSKNKRLFGIFGSMLLFYIIALLPYGLVAIINPLIQSYFRPEV
>Amphimedon_queenslandica|PAC1572824_rhodopsin
PTYAAVLGIEGVIGIIANVAVLLMTLYKSWNQPSTIFFTSLLLPNLIIALCYLM-SSIWI
FGNTFEQKNGSCMFTLAAISFDRFLFIVKPHLHVALILIIGVWLLSSMAFTSIWTFFFTR
SFIKRQGVYQSKNKKLFGIFGSMLLSYILSFFPYGLVVIINPLIQSYFRPEI
>Amphimedon_queenslandica|PAC1571848_rhodopsin
PTYAAVLGIEAIIGMIANVAVLLMTLYKSWNQPSTIFFTSLLLSNLIIALWYLM-SSIWI
FGSTFEQRNATCLFTLATVSFDRFLFVAKPYSYVALILVIGVWVMSSIAVTSFWTFCFTR
RFMQDQAVYQSRKKRLFGIFGYMLLSYVIALLPYGSFITSNPIXXXXKSYHS
>Amphimedon_queenslandica|PAC1571848_rhodopsin
TTYAAVLGIEGVIGIIVNVAVLLMTLYKSWNQPSTIFFTSLLLSNLIIALWYFM-SSIWI
FGNTFKEKNASCLITLAALSFDRFLFVVKPYLHVALILIIGVWLLCSIAFTSIWTFCFTR
RFIQNQVVYESRRKRLFGIFGYMLLSYVIALLPNGSFIISNPIIQSYFRPEI
>Amphimedon_queenslandica|PAC1572824_rhodopsin
TTYAAVLGIEGVIGMIVNVAVLLMTLYKSWNQSSTIFFTSLLLSNLIIALWYFM-SSIWI
FGNTFEQKNATCLFTLAAISFDRFLFIVKPYLHVALILIIGVWLLCSIAFTSIWTFCFTR
RFMQNQAVYQSRRKRLFGIFGYMLLSYIIAILIYGSFIISNPIIQSYFRPEI
>Amphimedon_queenslandica|PAC1572492_rhodopsin
PLLAAALAIQMIGALVANGIVLIATLSKSLKLPSTMFFTSLIIIHLLMALLFIPFYMIWI
FGRTVEEKEGTCKYTLAAISVDRWLFIVKSQLYVALTIIVSIWILAAIIATSVWTFIFTR
KFIQEQAVYVSRNRRLIGIFGAMLSGYAVCFAPFINVIIINPMIQSYFRPEI
>Amphimedon_queenslandica|PAC1572492_rhodopsin
PLLAAVLTLQMIGALVANGIVLIATLSKSLKLPSTILFTSLIIMHLVMALLYIPSWILWI
FGRTMQVKEGTCKFTLAAISVDRWLFIVKPQLYVALTIIASIWILAAILVTSVWTYIFTK
KFIQEHSVYVSRKRRLIGIFGVMLIAYFVCFSPFMMITIINPIIQSYFRPDV
>Amphimedon_queenslandica|PAC1571197_rhodopsin
PLLAVVLTLELIAALVTNTIVLAATLSKSLKLPSTILFTSLIMIHYVMAFIYILSWLIWI
FGTSEEEKEATCNATLTAISVDRWLFIVKPNFYVTLVLVLSIWIFSGVIVTSVWTYCFTK
KFIREHAVYVSKNRRLTGIFGLMLIAYVICYVPILAMTFINPIIQSFFRREV
>Amphimedon_queenslandica|PAC1571174_rhodopsin
PLLAAALAVEMIAALIVNTFVLVATFSKSLKLPSTILFTSLIMIHYVMALIYIPSWLIWI
FGNTMQVKEATCNFTLAAISVDRWLFIVKPIFYVALIVVASTWITSTIIVSSIWTCCFTR
RFIREHAAYVSKNRRIIGIFGAMLVAYGVCYTPLLTVIIINPIIQSIFRPDV
>Amphimedon_queenslandica|PAC1571687_rhodopsin
PLLAAALAVEMIAALIVNTFVLVATFSKSLKLPSTILFTSLIMIHYVIALIYIPSWLIWI
FGSTIQVKEATCKFTLAAISVDRWLFIVKPIFYLAVVLAVIIWIGSCIIVTSIWTCCFTR
RFIREHAAYVSKNRRIIGIFGAMLVAYGVCYTPFLTVIIINPIIQSVFRPDV
>Amphimedon_queenslandica|PAC1572314_rhodopsin
PAVAAILIIEMALGLVANSIVLVITFTQSWKQPSTIFFTSLILAHLVLLLLYLPFFIVWV
FGKTLQEKRDSCSVTLSVISFDRFLFIVKPNQYVALCLTITIWLLAAIIITSVWTMCFTR
NFLSHQSVYTSKKKRLFGIFGTMLIVYVICLSPFVAITFVNPLVQSYFRVDT
>Amphimedon_queenslandica|PAC1571278_rhodopsin
PAVAVILGLEMVAGLFANAVVLCITLMKSLSQPSTIFFTSLTAAHLLMVLLYLPVSVIWI
FGSNFEEKKITCSINLAVISFDRFLFIVKPHFYVALILTIAVWILSAIIVTSLWTFCFTR
KFMHEQEVYQSKERRLFGIFGAMLIVYGVCFLPFQLITSGSPLVQSYFRPDI
>Haliclona_amboinensis|Hamb_m_413_rhodopsin
PALAGVIAVLMVAALLANTLVLIVTVYKSWKQPSTIFFSSLIVSHLVMNLLFTPFFLIWI
FGETTEEKRATCSSTLALISFDRFLFIVKPHLYVAVALVVCIWLLTAIAVTSLWTFCFTR
KFLQEHSVYVSRKKRLFGIFGSMLLVYLLCFSPFMSVSVLNPIIQSYFRPDI
>Haliclona_amboinensis|Hamb_mm_53_rhodopsin
PAVAAVFAVEMIVALIANTTVLIITVTKFWKLPSTIFFNSLIVAHLVMVLLYLPFNIIWI
FGSTSEQKEDICMFTLALISFDRCLFIIKPNVYVALTLVTGVWLLTAITVTSVWTFCFTH

KFLRDHPVYISRKKRLFGIFGAMLIVYIVCFAPYQLVAIANPLIQSYFRPEI
>Amphimedon_queenslandica|PAC1571069_rhodopsin
PVLAAVLAVEAVVAFIANIIVLSITLYKSWKQSSTIFFTSLILSNLLAVVGYLPMNIVWI
FGSTFEERSITCLIILAAISFDRCLFVVKPHLHVALTLTIAIWIFCIIIITSIWTCCFTQ
RFINDQSVYASRKKKLFGIFGSMFLAYSFCYGPFQFITVASPLIQAYFRPDI
>Amphimedon_queenslandica|PAC1570073_rhodopsin
PVLAAVFAVEAVVGFIANIIVLSITLYKSFKQPSTIFFTSLILSDLLDVLVYLPMTTVWI
FGSTFEQRRATCVFVLAAISFDKCLFITKPYFYVALTITVALWIIIGIIVTSVWTFCFTR
RFINNQSAYNSRKMKLFGIFGSMLLAYTLCYAPLHFAPIVSPIIQAYFRPEI
>Amphimedon_queenslandica|PAC1570919_rhodopsin
PVLAAVFALEAVVGFIANIIVLSITLYKPFKQPSTIFFTSLILSNLLDALVYLPMTTVWI
FGSTFEQRKATCLFVLAAISFDRCLFIVKPYFYVALIIAVIPWMIIAIIVTSIWTFCFTR
RFIRDQSVYHVKKMKLFGIFGSMLLAYVICYGVFQFSIIATPLIQSYFRPDI
>Amphimedon_queenslandica|PAC1571700_rhodopsin
PALSAVLALETVTAFIANTVVLSITLYKSWKQSSTIFFTSLILSHLVMVFLYLPLCVIWI
FGSTFEQQIATCSFTLAIISFDRFLFIVKPRIHTALAFTIGIWILAAILITSIWTFCFTR
RFINDQSVYVSRKKRLFGIFGSMLLVYGICFLPFQFITVASPLVQSYFRPDI
>Amphimedon_queenslandica|PAC1571069_rhodopsin
PAVAAVLTVEMILALIANGVVLSITLYKSWKQSSTIFFTSLILAHLVLNLLYLPFTVIWI
FGSTDQVKRGFCKFMLAAISVDRFLFIVKPHLHVALTLTIAIWLLAAISLTSIWTFCFAR
RFLTDQSYYSSKKQRLIGIFGAMSLVYLLCFSPIELLTIAGPLVQSYFRPDI
>Amphimedon_queenslandica|PAC1571068_rhodopsin
PVAITVLSITTLAALIANSVVLFITLYKSWKQSSTIFFTSLILSNFILNLLYLPFTIIWI
FGSTDEEKRGTCAFTLAAISFDRFLFIVKPHLHVALTLTIAIWILSAIIITSVWTFCFTY
KFIRDQSVYLSRKKRLVGIFGAMLLVYVICLLPLHLFTVINPLVQSYFRPGF
>Amphimedon_queenslandica|PAC1571068_rhodopsin
PVVAAVLIIX---XXXANTFVLSITLYKSWKQSSTIFFTSLILANFVMVLLHFPFAVTWI
FGSTDEEKTGTCTFTLAAISFDRFLFIVKPLLHVALTLTIVIWILSAIVITSLWTFFFTW
WFLRKRSIYSSKKRGLLGIFGFMLLVYGISKGPYQLSIIGDPIVQSFFRPGL
>Amphimedon_queenslandica|PAC1571068_rhodopsin
PVVAAVLIIQVILALIANGVVLSITLYKSWKQSSTIFFTSLILAHLVLNLLYLPFTIIWI
FGSTDEEKRGTCTFTLAAISFDRFLFIVKPHLHVALTLTIAIWTLSAIAVTSVLTFCFTR
RFINNQSVYSCEKKRLFGIFGAMLLSYVVCFIPVLFVTTVNPLIQSYFRPEV
>Amphimedon_queenslandica|PAC1571068_rhodopsin
PAVAAVLSVETVAGLVANFIILSITLYKSLKQPSTIFFTSLILALLVLLLVYLPLSIIWI
FGSSFEEKSATCSFTLDAISFDRFLFIVKPRLHVALTLTIAIWILSAIVITSLWTFCFTR
QFMNEQSAYTSQKKRLFGIFGSMLIVYGICFTPILFITVANPIVQSYFRPEI
>Amphimedon_queenslandica|PAC1571068_rhodopsin
VAVGAALAVEGVSGFLANTFVLSITLYKSWKQSSTIFFTSLILANFVMVLLHFPFAVTWI
FGSTDEEKTGTCTFTLAAISFDRFLFIVKPHLHVALTLTIAIWTLSAIIVTSVWTLCFTH
RFITQQSTYGSKKKWLLGIFGSMLLVYGICFMPSLFITVANPIVQSCFRPEI
>Amphimedon_queenslandica|PAC1571068_rhodopsin
PAVAAVLTVEMILALIANGVVLSITLYKSWKQSSTIFFTSLILAHLVLNVLYLPFTIIWI
FGSTDDEKTRTCSFTVAAISFDRFLFIVKPHLHVALTLTIAIWTLSAIIVTSVWTLCFTH
RFITQQSTYGSKKKWLLGIFGSMLLVYGICFLPSLFITVANPIVQSYFRPEI
>Amphimedon_queenslandica|PAC1571068_rhodopsin
---------------IANGVVLSITLYKSWKQSSTIFFTSLILAHLVLNLLYFPFTIIWI
FGSTDEEKRGTCSFTVAAISFDRFLFIVKPHLHVALTLTIAIWTLSAIIVTSVWTLCFTH
RFITQQSIYASKKKWLLGIFGSMLLVYGIYFMPSFFITVANPIVQSYFRPEI
>Petrosia_ficiformis|Pfic_m_102_rhodopsin
AVHSFILSAIILVGVCGNAFVLFLVAKKRLRYRSVIASLSVVFVDFLLTIFYHGVALTWA
YGDDDFSI---CRMALGLIATDRFLTVRFPFHYL-IILTLLVWGCPI-------------

>Haliclona_tubifera|Htub_mm_64_rhodopsin
IPCAIILGFMNLLAFGWNLFIIIVYCYYLLKHPANIFLFSLAFMDFLMSVTVIPSSVIYV
IGSTDTVRCGFCYFILAMLSMDRCFLLSNPLVYVAVVVVTIVWMFCILGVTNIWTFRIVK
KFLRNKLKHQQQQAQLIKVFGALFIANTVIWAPVASNNVVHPILESFFNKEL

>Petrosia_ficiformis|Pfic_m_112_rhodopsin
IGIALVISILFVIAFTWNLFIIITYIVNLLKQPANIFLFTLAITDFMISLLVLPVSVVFF
IGDNDKTRCDVCYTILASLSVDRCFLLSQPIKYFAVVTIISLWFICSLCVTNIWTYKIVR
RFLKKRLSHQRQQTQLVKVFGALFIAHCITWTPTVSNSAVHPIIESFFNKEL
>Amphimedon_queenslandica|PAC1571292_rhodopsin
IAIAFFESIIFIVALSWNLFIFVTYLLRLLKEPANIMLFTLSIVDLLVCILIIPFPIIYI
FGNSDVVRCIICQVLLAILSIDRCILLSNPLKYTTVVGILVIWVFCFLAVTNVWTFKIVN
RFLKKNLTHRSQQNQLVKVFGALFIANIISWTPFLLNPTVHPILESFFIKEL
>Haliclona_amboinensis|Hamb_m_192_rhodopsin
IPIALFESIVLVVALGWNLFIFITYLIKLLKEPANILLFSVSITDILICILIIPFPLVFI
VGNTDVIRCVICSISLAILSIDRCILLSNPLKYTTVLGVAIIWFISFLAVTNVWTYKIVN
RFLKRNLNYKTQQKQLVKVFGALFITNIASWTPYLLNPTVHPILESFFIQEL
>Amphimedon_queenslandica|PAC1571293_rhodopsin
IPSAVIQSLILAVALGWNLFIIVVFILELLKEPANILLFTLAIVDVLICLIVVPGPIVFV
LGRNDQIRCAICDTTLALLSIDRCILLSNPMKYLWVLFIVLVWLLCLIIITNIWTFKIIL
SFLKRKQQIKQQQKQLVQVFGALLIATIIAWVPYLINPLVHPILESLFVKEL
>Haliclona_amboinensis|Hamb_m_242_rhodopsin
IPSAVLQSIIFCVATGWNLFIIIVYIIQLLKEPANLLLFMLAISDLMVCLIIVPVAISFV
LGHNDSTRCIVCSIFLAMLSIDRCILLSNPFKYIWIVIVLFVWLLCFIIFTNTWTYKIVR
LFLKKKLQQQQQQKQLVNVFGALVISNVVAWVPYLINPLVHPILESFFIKEL
>Petrosia_ficiformis|Pfic_m_114_rhodopsin

MFVIT--VYSNSYRNLIGIFGALTVVNVLSFLPYLLSNIFNPIIQSYFRQEL
>Haliclona_tubifera|Htub_m_420_rhodopsin
PLIASVVLFEALASLLANSFVLTVTMWQVLKQPSIIFLTNFVVLNLFLTILYLFPVVIWI
FGDTVEQKNGSCQFTYAIISVDRLFFIVKPMHYVAVAVIGFAWSSAIIVVTNIWTFCFTR
MYLQRARIYDQKIRKVVGIFSALLAMTVVVYIPYYFNYFLNPLIQSYFRKEL
>Haliclona_tubifera|Htub_m_168_rhodopsin
TAYATAIIILIVLAIVVNMFVLIYTLCKTLKQPSIVFLTNFVMVNLLMVFIYLPSVVIWI
FGHTNSEKSASCQFMLTAISVDRFLFIVKPLVYVAVVVSAVLWIVALIAVTSIWTYCFTH
NYIKRTGVYNTKLLKIMGLFGAMLLVTAVTYAPYFTTTVSNPIIQLYFRRDM
>Haliclona_tubifera|Htub_m_131_rhodopsin
TAYATAIIILIVLAIVVNMFVLIYTLCKTLKQPSIVFLTNFVMVNLLMVFIYLPSVVIWI
FGHTNSEKSASCQFMLTAISVDRFLFIVKPLVYVAVVVSAVLWIVALIAVTSIWTYCFTH
NYIKRTGVYNTKLLKIMGLFGAMLLVTAVTYAPYFTTTVSNPIIQLYFRRDM
>Haliclona_tubifera|Htub_m_432_rhodopsin
PALVIFIGLEIIVALCANSFVLLYTLCKVLKHPANIFLTNFVLGNLLMTVLYMPSMMVWV
FGTTLDEKVASCQFTLTLISIDRFSFIVKPNLHYAVIYSILSWITSAIAVTSIWTFCFTR
SFIKKIRVYKNRMCNIVGIFSAMLVVMVASFALVLLNNVLNPIVQSYFRREL
>Haliclona_amboinensis|Hamb_m_238_rhodopsin
PLLATVIGMEMIIALIINVAVLVGTFASSLKKPSTIFLSFLVVSNIIMAIFFMPFTVIWI
FGETDQQKQAVCEFTLALISVDRCIFIVRPLLYLALLIIICLYPILTITVTSVWTFIHTR
RFFKRRQVYNRKIYNLIGIFGSLLIVHLISLAPHLLHTITNPLVQAYFRPEL
>Haliclona_tubifera|Htub_m_186_rhodopsin
AALAVAVGIEFVLSLVSNLFVLIFTLCKSMKQPSIIFMTNFVLANLMITILVMPFTIIWI
FGESVEQKHHVCQFTLVVISIDRFLFIVKPLFHVAIAIVICVWILSTISITSIWTCCFSR
KFIKKAYLYTHRMRKLIGIFGILIIITIVSYVPFFVNTVANPIVQSYFRRDV
>Amphimedon_queenslandica|PAC1571393_rhodopsin
PAIAFFLSLEMGISLAINSTILGIIFLSLIKAPSNIYLTSMLLVNLLATLTVMPMIIIWI
FGGTLEEKVSSCRFTLAIISVDRWMFIVKSTLYVAIGVVITAWLLTAIAASSIWTFCFSR
RFIRRMSVYTFRWRKLIGIFGTLSIVYVVCFTPFLLITILNPLVQIFFRRDI
>Haliclona_amboinensis|Hamb_m_739_rhodopsin
PVVAAFVGLEMITALITNVFVLCITLCANWRQASNIFLTSMILVNLVVAITVMPFIIIWK
FGSTIEQKVGTCEFTLAIISFDRFVFIVKPAYHVAIGIVIAVWVVCAIIVTSIWTFCFTR
RYIKRIRIYTSRWRKLIGIFGTLSIVYALSFAPFYLITILSPLVQSFFRNDI
>Amphimedon_queenslandica|PAC1571347_rhodopsin

PLLAGAIGIVTVVALLTNSFVLLLTLCKAWKQPSNIFLTNMLLSNLLISVFLMPLCVVWI
TGATDAQKLKTCQASLTLISLDRLFFIVKSMEYKALLIVLLSWLLAAIVISSTWTFCYTR
NFLRRRNVYLSQKRKLIGLFGTLVVIHILCYSLLLLMTNLSPLAQSYFRYEV
>Amphimedon_queenslandica|PAC1572622_rhodopsin
PLLAAVIGVEMVAGLIANIFVLTLSCFETYKKPSTIFLTNMLVANLIIIVVVMPFSIAWI
FGSTVSSKLASCEASLVLLSFDRFFFIVKAGKYKALIIVAASWILAAIVVTSLWTYCYTK
KFIKEMNVYFSQHTKLVGIFGTLIIVHIFCYSLLLLITILSPLVQSYFRSET
>Amphimedon_queenslandica|PAC1572437_rhodopsin
PLLAAVIGLEMITGFLTNSFILVLTICKTWKQPSNIFLTNMLLNNLVIVVIVTPFSIVWI
FGNTLSEKVAICEASLVLISFDRFFFIVMSFEYRAIIIVIISWMLAAIIVTSTWTYCFTR
RYIKRRSEYQSQNRKLVGLFGMLIVIHILCYTLVILMTSLSPLAHAYFRYDI
>Amphimedon_queenslandica|PAC1571394_rhodopsin
PLLAAVISIEMIAGLIANSFVLIITICGTWKKPSTLFLTNMLICNLFTVLLVMPLNIIWI
FGVTVDQKLITCNLSLMLLSIDRWIYIVQAMRYRALLAIIITWSLAIIVVTSCWTFCYLF
MYIRRRKQYVAARRKLIVLFGVLLFIHFISYLPFLSITTLSPLAQSCFREDI
>Amphimedon_queenslandica|PAC1571393_rhodopsin
PALASIVGLELFAGFITNSLLLALTVFKNWKLPSTVFLTNLLLCNLLLVLLVMPFTIIWV
FGRNPVEKRTVCRIGLVILSFDRFFYVVKAVAYKAVTIVIATWVAGGIVVTSIWTCIFTR
RFLGKDELYATKNQRLLGLFGMTVLVHLLCYLPFLMAATLGPLVQSFFRRDI
>Amphimedon_queenslandica|PAC1570935_rhodopsin
PLLAAAFATEMIGGLITNSLVLILTASKTWKQPTTIFLSNMLLNNLVIGICIIPFAIIWI
FGRTEKEKETVCQVSLVLLSFDRFFFITKSFQYKALVIVSLSWALAVIIVTSIWTLCFTR
KHIKKTASNHTQERKVIGVFGMLIIVHLLCYAPLFLLITLIPLVQSFFRSDI
>Amphimedon_queenslandica|PAC1571245_rhodopsin
PLLAAVIGMEMLAGLITNSFVLILTACKNWKQPTTVFLSNMLANNLVVILFTMPLSIIWI
FGSTVSQKESVCYFSLVLLSFDRFFFIVKALHYRAFIIVAVSWLLAAITVTSLWTMCFTR
KYLKNAA--------------------------LLLLTVLIPLVQSFFRRDI
>Haliclona_amboinensis|Hamb_m_333_rhodopsin
PVFATVVGIEMVTALITNSFVLLMTVCTTWKQPSTVFLTNLLLANLCTAVFGMPFIVIWI
FGNTLDQKVKVCQFSLVLISFDRFFFIVKALLYKAVAIVVVSWILVAIIVTSVWALCFTR
NFLKRRSVYISQKRRLIGLFGTLIIVHLLCYLPFLLETTLSPLVQSCFRKDI
>Amphimedon_queenslandica|PAC1571394_rhodopsin
PLLAAVISIEMIGGLIANSFVLILTICKTWKQPSTIFLTNMLISNLLIVLFVMPFAITWL
FGKTDKQKVKACQFGLVLLSFDRFFYIVKSFEYISIIIVTLSWLLAAIIITSSWTMIYTR
RYLNMERIYASRKKRRVIGLFGMIMIVHLLCYLPFLLLTVLSPLVQSFFRRDI
>Amphimedon_queenslandica|PAC1571394_rhodopsin
PLLAAVISVEMIGGLIANSFVLILTICKTWKQPSTIFLTNMLISNLLIVLFVMPFPITWM
LGETEAEQVKYCYFSLVILSFDRFFYIVKSFMYRSLSIVAFSWLIASIVTTTLWTYCYTR
RFLQSERVYISKEKRLIGLFGMMMVVYLTCYAPFLLITVLSPLVQIIFRRDM
>Haliclona_amboinensis|Hamb_m_380_rhodopsin
QPLPIVVWVVWFTAVVSNLAVLFVML-KEKKQVPQFFICNLALADFLLGIYLAFLGAVFY
KSALLWQMGPGCQTILTALTLERLHTIAYSFKRYAILIVAIGWLLAGITAFLVILCSYVR
ILYLF-CTSGKDKIVIALKMGTLVLTNLICWLPFPFNACFNPFIYSIFTRQF
>Haliclona_tubifera|Htub_m_134_rhodopsin
DPVRVLIWVVWFPAILGNIAVIFVIM-SEKVDVPNFILCNLALADFMLGIYLSFLAVVFY
KSALHWQHGPGCKTLMVILTLERLHTIVYSFNQHAVILVVIGWVFAMVVASLIILFSYIH
ILVIC-C---RERIMTSVKMGILVVSNFACWLPLPINACLNPFLYTISKKTF
>Haliclona_amboinensis|Hamb_m_274_rhodopsin
DALRAGMWLVILISIGGNVLVLTVTGLRKKPHIMYFLYLNLAIADLFMGIYLLTIAVVYS
LHAIDWQTGPGCRFTLLIITMERVYTIKFALRRYAVISILIGWVITLGIATFVIMMCYCL
LFYMVACISGREELKLALRMSLLVITDFACWAPFPINSCLNPILYSFSTRKF
>Haliclona_tubifera|Htub_mm_39_rhodopsin
DILRVAIWLVLFIAIIGNGLVIALTILKKEPNLLYILYLNLAIADSFMGLYLLTVASVYS
ADAVEWQTSAGCNFSLLFITIERVYSIKFALEMWVFIFMVFGWILAFGVASFIILICYVY
LFYLVGCFSGKEELKLALRMSLLIVTDFATWGPFPLNSCLNPILYSFSTRLF
>Amphimedon_queenslandica|PAC1572135_rhodopsin
NLLRAGMWLVIILSIGGNIVVLSATLLHKKPHLMYFLYINLAMADLFMGIYLLTIAVVYS

RHAIEWQTSAGCRFTLLVITVERVYTIKFALQHTVTVSILVGWVLTIGIASFAIMFCYVL
LFYLVVCLSGREELKLALRMSLLVMTDFACWAPFPLNSCLNPILYSFSTRKF
>Petrosia_ficiformis|Pfic_m_169_rhodopsin

-------------FTLLVITVERVYSIKFSLQRWVCFFMIIGWVIGIGIASFVILFSYVY
LFYIVACLTAREELKLALRMSLLIVTDFMCWGPFPLNSCLNPIL--------

Disks large homolog (DLG)

>Amphimedon_queenslandica|PAC:15726976_disks_large_homolog_(DLG)
LAEKWEDSNPGRGLGLSVAGGTDNIFITRLTPGSPAERSGLLQLGDQLLSVNNVPLVDVV
HNDAVDALRNAGLTVTLRIRRNGGTSLGFSIAGGKGNQHVLDDNGIFVTKITKGGVADQD
GQLEVGDRVLEVNGQNMVEIDHEDAVAILKATGQEVTLKIEKGVGLGFNIIGGEEEVGIF
ISVISKEGVAADNGQLRVGDMILEVNGQNLETWSHETAAQALKTAGETVTLKVVYKPDEF
EEFYRKKTLYVSSSPNIS
>Ephydatia_muelleri|Ephy284344_disks_large_homolog_(DLG)
LAERWEGHTSGGSLGVSVAGGVDNIFITKILPDTVAEQDGRLRLGDQIVSVNGVCLEEVN
HSRAVDALKQAGQHVTLVIRRKNAQGLGFSIAGGCGNQHVLGDDGIFITRIISGGAADEN
GTLAVGDRILEVNGNSMVSITHDDAVRILKSTQEKVMLRIEKGTGLGFNIIGGEAGTGIF
ISLISPNGVADKSSQLKVGDQILEVNNQDLRTATHEEAATALKNAGTVVTLRVEYRPEDF
SDFQNKKQLYVRALFDYD
>Haliclona_amboinensis|Hamb_m.15210_disks_large_homolog_(DLG)
LTEKWEDSDPGRGLGLSVAGGTDNIFITRLTPGSPAERSGLLQLGDQLLSVNDSSLIDVT
HNEAVDALRNSGQRVTLRIRRKKGGGLGFSIAGGKGNQHVLGDDGIFITKIIPGGLADSD
GRLEVGDRVLSVNGQSTVDINHEEAVAILKATGQDVVLKVEKGVGLGFNIIGGEEEVGIF
ISVISKEGVAAESGQLRVGDMILEVNGQNLENWSHETAAQALKTAGEEVTLKVIYKPKEF
EEFYRRKQLYVRALFSYD
>Haliclona_tubifera|Htub_m.26123_disks_large_homolog_(DLG)
LADKWDKHQPTRGLGLSIAGGNDNIFVTRLTPESPAEKCGLIQLGDQILSVNGTSLIDIS
HVDAVNTLKSAGNRVELHIRRKKGRGLGFSVAGGHNNQHIEGDDGIFITKIIEGGVAEEE
GTLAVNDRILEVNGKSMVKITHEKAVDILKGTGKDVKIKVEKGTGLGFNIIGGEEEVGIF
ISVLSPQGLAAKSGQLKKGDMILEVNDQDLVRWSHDAAALALKNAGDVVKLKVVYKPKEF
EELYRRRELYVRALFSYD
>Leucosolenia_complicata|29073|lcgid16959_disks_large_homolog_(DLG)
MAMRWEKQSPGRGLGFSIAGGVDNVYCTKVLPNGVAMEDGRLRADDIIVQVNDTVMDNVT
HAQAVQSLKDAISTVVLRIRRKKDRGLGFSIAGGVGNQHIEGDDGVFVTKIIEGGVAEAE
GTLQVNDKLMKVNDTVLDHVSHAEAVQALQRAAGTTYLTIEKDKGLGFNIVGGEGGEGIY
ISFILAGGVADLSGQLKRGDQILDVNGNDLIQASHESAAGVLKGSGRTVTLLVAYRPEEY
GRFEQRKSSHVRALFDYD
>Oscarella_carmela|Ocar306928_disks_large_homolog_(DLG)
MAAAWEKQSPSGGLGFSIAGGYDHVYVTKIIPIGGASKDGRLRLEDCILSVNENSLLNVT
HGEAVDALRHAGNRVTLVVKRKAGKGLGFSIAGGVGNQHIPGDDGIFVTKIIDTGAAAAD
GHLSVGDRILKVNEGSVENVSHEDAVLALKRTDQTVILLVEKNQGLGFNIIGGEDNQGIF
VSFILAGGAADRSGKLVRGDQILDVNGTNVRGASHGDAAVALKSAGDTVRMRVQYNPEEY
AQFEAKKELFVRALFDYD
>Petrosia_ficiformis|Pfic_m.12493_disks_large_homolog_(DLG)
LTSNSRTTSPPEGFGFNVMGGREQIYISRIIPGGYSDRQGNLRRGDQILSVNDVSLQDVT
HADAVNVLKGAGTRVEIRIRRKKGGGLGLSIAGGTNNQHVEGDNGIFITNIIANGVAEED
GRLGVNDRIIEVNDKTMVNVTHEEAVEILKGTGKDVYIKVEKGKGLGFNIIGGEEEVGIF
ISVISDDGIAAKSGQLKIGDMILEVNGENLERWSHDTAAHALKTAGDHVTLKVIYKPKEF
EELYRRRQLYVRALFDYD
>Sycon_ciliatum|36247|scgid29339_disks_large_homolog_(DLG)
MAMRWEKQSPGKGLGFSIAGGIDNVYCTKVLPNGVAKEDGRLRADDIILQVNDTVLELVK
HSDAVQALKDSTNTVVLRIRRKGSRGLGFSIAGGIGNQHVEGDNGIYVTKVIPGGVAEVE
GSLLANDKLLKVNDTALHDVTHAEAVQSLQRASGTTYLTVEKDKGLGFNIVGGEGGEGIY
ISFILAGGVADLSGQLKRGDQILEVNSTNLTDASHESAAQALKGSGKTVGLVVAYRPEEY

GRFEQRRSSHVRALFDYD

Membrane-associated guanylate kinase inverted 1 (MAGI)

>Amphimedon_queenslandica|PAC:15725500_Membrane-associated_guanylate_kinase_inverted_1_(MAGI)
SNNDPLGRSNSLKRKHWSIRAHETLISRNKDGHFCLNVEGGAEHGIFPLIGDLRQDRINY
KMGKLHPGELIIEINHKRVPGMIKKDVIALIKRSADPVSLVTVKQNATITKDIRHYLASR
FTKNSVDSELQHQIRENLHTRVIPCTTRQPRDEERNGVDYNFISVEEFKKMDKQGLLLES
GTYENNHYGTPKPPPDPPSGPLPSNWEIAYTENNEKYFIDHNTGTTHWLDPRLVHNLKHS
LLDCGDDELPYGWEKVNDPMFGVYYIDHINRTTQYENPVVEAKRKFVGSQLEIESNLRGD
PIFVTLTKNPTGFGFTIIGGDRPGELLQIRSIVQGGVADRDGNLKIGDVLVRVNGESVVA
HNHKRVVDLFQSIPMHSPVRLEVRRGYPLPDSMTDELPSYSESNKFSLGPLPPPEKVVVG
IVKGPLGFGFSLSETPQGPVKQIMDIPRCAQLREGDLITELNGVLSLTHTAPA-------
-----------EPFQHMIVMLERQVSGFGFRVIGGREEGSQATIGGIVPGGAADLDGRLM
VGDEITQINGLSVMDAPHKDVIQLIAQAGQVGKVELHVRRKMPWPMSGPLQSNTLRTGCM
ICRLIPDSPAEKCQQLYLYDELLVVNMDHGDIVGLIKGSSTTIKLVV
>Ephydatia_muelleri|Ephy107470_Membrane-associated_guanylate_kinase_inverted_1_(MAGI)
--MSKAKRSDSLQKKHWSIKAHESLISRNAEGHFCLNVEGGAEAGLMPFVGEIRQDRINY
QSGKVYAGEIILEVNSKRVPGMIKKDVIALIKKSTDPVSLVTTKQNATITKDLRQYLATR
FTKNSVDSELQHQIRENLHMRVVPCTTRSRQGNEQQGVDYNFISPEEFKRMEKNGDFLES
GSFEGHFYGTPKPPTDPPSGPLPPNWEIAYTENNEKYFIDHSTGTTHWMDPRLAHMMKHT
LLDCEDTELPYGWEKVSDPVYGTYYIDHINRVTQYENPVTEAKRRYAGAQLDLEADLQGE
VVKTILLKTAAGFGFTIIGGDRPGELLQIKTIQKGSAADKDGRLQVGDVIVYINGILVLT
YNHHKVVDLFKSIPMGSTVTLEVRRGYPLPDYQDDKLPPYTASS------HAVMDRLSIN
IVKGPLGFGFSLGESTRGPVKQIMDHPRCAQLREGDIIMEVNGVHNYLHSDLVSVLKRCP
KGNQANFGVIREPFTVLEVRLIRQVSGFGFRIIGGKEEGSQATIGAIVPGGAADLDGRLQ
IGDEITQINGRSVLDAAHQDVINYMGEAAAQGEVTLKISRKVPPLELNPLQSNTLRAGCI
VCRLVQGSPADQSGQLYQFDELISVNMDHSDVVTLIKASGTTITLEV
>Haliclona_amboinensis|Hamb_mm.390_Membrane-associated_guanylate_kinase_inverted_1_(MAGI)
SGTDPLSRSNSLKRKHWSIRAHETLISRNKDGHFCLNVEGGAESGLFPLIGDLRQDRVNY
KMGKLYPGELIIEINHKRVPGLIKKDVIALIKRSADPVSLVTVKQNATITKDIRHYLASR
FTKNSVDSELQHQIRENLHTRVIPCTTRQPREGERDGVDYNFISVEEFKKMDKNGQLLES
GTYENNYYGTPKPPPDPPSGPLPSNWEIAYTENNEKYFIDHNTGTTHWLDPRMVCFLKHS
LLDCEDTELPYGWEKVNDPRYGSYYIDHINRTTQYENPVVEAKRKFLGSQLEIEASLRGD
PIIVTLTKNPTGFGFTIIGGDRPGELLQIRSIVQGGVADRDGNLKVGDVLVRVNGESVVA
HNHKRVVDLFQSIPMHSPVRLEVRRGYPLPDTIGDELPSYSESNKFSMGPFPPPEKVVVG
IVKGPLGFGFSLSETPQGPVKQIMDIPRCAQLREGDLITEVNGVLSQSHSDLITLLKRCP
KGNTANFLVTREPFQHMVVVLERQVSGFGFRVIGGREEGSQATIGGIVPGGAADLDGRLM
MGDEITVINGLSVMDASHKDVIQLIAQAGQVGKVELHIRRKMPWPMSGPLQSNTLRTGCM
ICRLIPDSPAEKCQQLFLYDELLVVNMDHGDIVGLIKGSSTTIKLVV
>Haliclona_tubifera|Htub_m.37365_Membrane-associated_guanylate_kinase_inverted_1_(MAGI)
SSGGQVGRSNSLKRKHWSIRAHETLISRNKDGHFCLNVEGGAEYGLFPIIGDLRQDRINY
KMGKVHSEELILEVNHKRVPGMIKKDVIALIKRSADPVSLITVKQNATITKDIRQYLASR
FTKNSVDSELQHQIRENLHTRVVPVTTRKPRDGERDTVDYNFISVDQFKKMDKAGELLES
GTFENNYYGTPKPPADPPSGPLPGNWEIAYTENNEKYFIDHNTGTTHWLDPRMARSMKHS
LLECDDNELPYGWEKVEDPNCGTYYIDHINRNTQYENPVTESKRKYHASQLEIEDNLIGD
VIKVVLTKNPTGFGFTIIGGDRPGELLQIRSIVSGGVADRDGQLKVGDVLVRVNGESVVT
YNHHKVVDLFQSIPMHSPVELEVRRGYPLPDSAADELPTYSESN----ANTGSPDKILVS
IVKGPLGFGFSLSETPQGPVKQIMDKARCAQLKESDLILEVNGVFTHSHSDLITLLKRCP
KGNTANFLVMREPFQHLEVHLVRQVSGFGFRIIGGKEEGSQVTIGGVVPGGAADVDGRLE
VGDELTHVNGITVVDASHKEVIQLIAQASQMGDVLLGIRRKMPWPTNVPLQSNTLRTGCM
ICRLIPGSPAERCGKLFLYDELLIVNMDHGDIVQLIKGSDITIRLVV
>Leucosolenia_complicata|39710|lcgid12126_Membrane-associated_guanylate_kinase_inverted_1_(MAGI)

SKFSPARKSSLKKKKHWSGRVHDSVISRSAEGLFNVVIDGGGDYGQFPCIGDLKQDKINY
ISGKLHQDEILLEVNGQAVSGLTRQDVCRLIKESPDPLALRTVKTGSSITKDLRPYLGAR
FDKGTIDHDLQQTIRDNLYTRTVPCTTRDPRPGETPGVEYNFLTVDQFMVLEKSGNLLES
GLFDGNYYGTPKPPHEPPVGPLPRNWEIAYTDGGDKYYIDHTAGTTQWLDPRVARMQKRL
PLQCLDEELPHGWERVDDAKYGVYYIDYINSRTTMENPVARARRIGSNDIWEQAQRLNGL
VVEVRLRKTSSGFGFTIVGGEAPRDLKQIKNIVPGSVAAQSGRLQVSDVLVRINDVCVLL
FTHDDVVRMFQEIAVGGDVSIEVRRGYQLRN--GDELPQYIGTNKRMEPSRPDAERMIAH
VVKGPFGFGFTIADSALGQIKQILDAPRCQQLVEGDLLVEINGVYSASHNDVVTILKGCE
KGSSVEFLIERESYIYLEIELHRQVSGFGFRILGGLEEDSQVMVGTIVPGGAADLDGRLQ
VGDDIIEINGQSVLNETHRNVVQLMGTAGLHGRVALGIRRKTSQSGDSSLQSHVQEAGCS
ICRLVPDSPAELCGKLRISDRIVGVNLTHSEVVAAIKRAGLRVVLQV
>Oscarella_carmela|Ocar74304_Membrane-associated_guanylate_kinase_inverted_1_(MAGI)

----MAS-EPDMGGGNHSPMLGTYTTSATAN-----------------------SSLSNV
SF--GVAEGSSGSGDSFGKESMPAHVLAVGSI--KSVKSLKGEDEAEDIIVFIDEPSKRL
FCTL-CNRVFRD-----PVI----------TTCGHSFCRFCATVKF--GC--KPGMSFGE
YVRDPNGCPFKIKINNRKEPTQCPNSLHCPSLLKRDLESHLAVTFRGMQERIAEHLTSCR
FEVVKDFLSTV--MTELRAQIEDKDTDIGF-----------------LRSTVSNLSERLG
MLERSSEIRLQ-----------RLESNNSRVGQDLLDTRHTLSYMTNELMHGNRLYSGSA
DCTI----------RIWDIDSLECIDMHRHQMVHELTGQNHWVRALV
>Petrosia_ficiformis|Pfic_m.15448_Membrane-associated_guanylate_kinase_inverted_1_(MAGI)
SSGGQLGRSNSLKRKHWSIRAHETLVSRNKDGHFCLNVEGGAEYGLFPAIGELRQDRINY
KMGKMYHDELILEVNHKRVPGMIKKDVIALIKRSADPVSLVTVKQNATITKDIRQYLASR
FTKNSVDSELQHQIRENLHTRVIPITTRKPREDERDGADYNFVSVEHFKKMDKNGELLES
GIYENNYYGTPKPPGDPPSGPLPSNWEIAYTENNEKYFIDHNTGTTHWLDPRMARNLRHS
LLECEDDELPYGWEKITDPVFGVYYIDHINRCTQYENPVIEAKRKYHGSQLEIEANLKGE
VLKVILTKNPTGFGFTIIGGDRPGELLQIRSIVQGGVADRDGQLKVGDVLVRVNGESVVT
YNHHKVVDLFQSIPMHSPVELEIRRGYPLPETMGDELPSYSESNKFNLGPLPPPERHMVN
IVKGPLGFGFSLSETPQGPVKQIMDIPRCARLKEGDLILEVNGLFAHAHSDLITLLKRCP
KGNTAN--

>Sycon_ciliatum|46130|scgid34650 647_Membrane-associated_guanylate_kinase_inverted_1_(MAGI)
SKFSPARKSSLKKKKHWSGRVHDSVISRSSGGLFNVVIDGGGDYGQFPCIGEVKQDKIAY
ISGKLHPEEILLEVNGQAVSGMTRQDVCKLIGESPDPLVLRTVKTGSSITKELRPYLGAR
FDKGTMDHDLQQTIRDNLYTRTVPCTTREPRPGETPGVEYNFLTVDQFMVLEKSGNLLES
GLFDGNYYGTPKPPHEPPSGPLPRNWEIAYTEGGDKYYIDHTAGTTQWLDPRVARMQKRL
PLQCNDDELPHGWDRADDVKYGVYYIDYINGRTTLENPVARAHRIGSNDIWEQSARLNGM
AIDARLKKPANGFGFTVVGGQVPRDLKQIKSIVPNSVAAQCGRLQIGDVLVRINDVNVLL
FTHDDVVRMFQDIPIGGDVVIEVRRGVQQPP--GEFTPHPGHGQARFEPSRSGSDRLVAH
VVKGPFGFGFTIADSTIGQIKQILDAPRCEQLREDDWLIEINGVAGGTHNDVVTILKGCE
KGSSVEFLVERGAVTYLEIELHRQVSGFGFRILGGLEEGSQVMVGTIVPGGAADLDGRLQ
VGDEIVEINGQSVLNETHRNVVSLMSTAGLHGRVSLGIQRRPGTQQAGPLQSHXXXXXXX
XXXXXXXXXXXXXXXXXXXXXXXXXXXXXXXXXXXXXRQSSSSSSNS

Spongin short-chain collagen

>Amphimedon_queenslandica|PAC:15716019_spongin_short-chain_collagen
GPPGQNGDPGPTGLPGNPGPRGVV---GSPGALGPPGPPGS------------------P
GELGEQGTKGARGGSPGRDGSPGAPGQAGQDGNSGRNGEQGPPGPEGLPGHTGPVGSTGA
KGAAGRPGPPGAPGNPGEKGDKGDQGPRGPYGHKGEKGNSGLPGNPGALGESGGRDGDNG
QPGAPGLPGASGPPGEAGPDGPPGPQGVPGWPGGVSGAPGQRGPRGNTGRRGLMGINGNP

----GPSGAQGPQGSRGTPGPPGPQGNKGDRGTQGPPGSPG-------------------
--EQGFEGEAGQ---RGPAGQPGDAGVPGRDGEP---------GHSGPSGDHGVAGKDGQ
PGPTGPRGEKGGDRGPPGDVGAPGIAGSAGLTGSPGVDGRDGDTGPPGPAGAAGPRGGPG
YLYGDRGERGEQGPKGPTGET---GPQGLPGSAGVNGQSGQQGPPGPPGPPGGTPGAPGS
PGDQGEQGERGDSGEDGLPGQQGPP------------GESGGSQGETGPTGPDGAPGNPG
SEGQRGETGPPGATGPQGPPGLDGGDTGSAGDKGSKGSKGNRGATGAPGNDGTPGKEGGD
PGPPGSSGPQGPAGSAGDAGAKGGAAGPTGAQGPKGGRGQKGDRGTPGQPGPNGAPGTSG
DHGGTTGAPGNLGPIGPPGPPGAVGEPGSNGEDGLDGAGEPGPQGPKGPSGNRGDTGLPG
PVGSPGDPGPKGSSGDKGAMGPRGNRGNTGAQGPQGEKGGASGAAGANGNPGDDGEKGDS
GQTGPGGDRGPQGDVGPKGGELGPPGEKGSKGQVGPGPSGHKGEQGANGAPGADG--TGP
---PGPQGPQGDQGLTGQNGAKGEKGGLKGTEYYIG

>Ephydatia_mueleri|P18503.1_spongin_short-chain_collagen

-----------------------GPQGPQGVAGPPGIDGAKGDKGECFYPPP--------
----------------PTCPTCPAGPPGAPGPQGAPGAGAPGLPGPAGPQGPKGDKGLPG
NDGQPGAPGAPGYDGAKGDKG---DTGAPGPQGPKGDQG------------GDQGYKGDA
GLPGQPGQTGAPGKDGQDG------------GDKGQGPAGTPGAPGKDGAQGPAGGPAGP
AGPVGPTGPQGPQGPKGDVGPQGPQGPAGETNVYSG

>Haliclona_amboinensis|Hamb_m.43066_spongin_short-chain_collagen

---GMKGMIGPVGLRGMKGSPGIEGPPGPPGRKGLPGPPGDTGEKGSIGPRGNTGDIGPP
GEKGSNGTKGEK-GDRGIRGDRGPKGDPGSIGNTGKMGQKGQIGPKGDKGRRGPQGDKGD
KGMRGEKG---MKGEPGEPGRKGDTGPQGDKGKKGEKGDIGEKGMPGERGPVGG---LN-
ATGERGPTGEKGNMGETGPKGDTGPQGNKGDKGGMNGTRGDRGPKGNIGPRGHPGDKGAT
GPKGGVAGRIGKPGRRGFTGEKGDKGDRGEKGVQGRAGKPGVLGDKGNKGMMGPKGVRGD
RGSKGSNGTTGDMGPPGTRGPPGKQGLPGRKGDIGPKGSPGMKGSKGDNGRMGFIGKTGP
PGMNGMNGTMG-----KGFRGYPGARGSPGMKGSPGVEGMKGDPGPKGNMGLLGEKGGPP
G------RMGLKGSKGDKGDKGPKGDHGMNGTDGLPGKNGSQGFPGLSGRPGGMKGNTGP
KGDKGSIGDKGAKGLIGLPGARGSPGRKGDKGVTGERGLNGGNEGERGPKGQNGTVGMKG
PRGDPGSDGTPGARGPPGVKGQKGGMKGMKG------VNGTKGIKGDPGLRGKPGKDGGR
TGPPGKDGPVCMKGQKGEPGKMGGPPGMKGEKGAPGFDGDKGN---RGQAGSNGIPGIPG
RSGGMKGAKGNKGEFGLKGEKGDTGPSGRDGLRGDNGTGMPGRNGTDGMKGEKGDKGDKG
TKGVPGLPGMKGDKGMKGSPGNVGRRGK---TGPKGDMGGIEGPMGPQGRPGR------R
GLPGKQGEKGVMGSTGLKGGNEGPSGIKGTKGEKGPGPHGQPGPPGVQGPVGAEGGSQGP
PGRVGRPGPKGMKGIKGNIGSKGNRGPGMNTMVFRN

>Haliclona_tubifera|Htub_m.16710_spongin_short-chain_collagen

GPQGQKGAPGLKGIRGAKGHPGVKGPIGEMGQQGLPGPSGPPGVKGAIGRGGPSGFKGPT
GEKGEMGDPGPTGGSRGSPGNEGAPGNPGSTGPKGETGPIGHPGPAGQRGGRGDPGEDGD
VGKTGAHGAPGDRGEPGAPGPK------------GLDGGIGLPGQPGRD---GGQDGFDG
ATGAPGNPGSQGDEGDVGPIGYPGPQGPKGPGGGADGITGPRGPKGKKGEPGEDGHTGKP
GTKGGDAGQQGGQGDIGSPGANGPVGSPGEKGPLGRPGIPGLNGRPGNKGTSGATGPRGN
PGSPGQNGDDGQPGAPG---SPGAPGSPGRTGRAGRPGRNGAAGLPGPDGRQGPVGEPGH
PGPRGATGAVGGNDGTPGLPGGPGSSGAPGERGHPGAK---GPTGPQGPIGSIGDPGGPA
GIVGAPGNKGVQGEEGDKGHPGPQGEVGFPGEAGKTGAQGSQGEAGDSGP--GERGDPGP
PGHTGPAGDIGEEGEAGHPGSAGAPGSAGSKGPRGPPGENGGPQGEPGIPGAQGFPGNDG
AEGPPGSAGPRGAKGPSGPQGDQGGPPGSAGPQGRRGTQGSRGEDGEPGPQGHPGETGGS
PGAPGDSGPAGLRGMTGEMGPTGGPVGPPGE---HGLDGDKGEPGHPGQPGNNGAPGQPG
APGGRRGNQGPRGPIGATGPPGASGPPGRTGIEGTTGAGEP------------GEDGFPG
FDGAPGQPGAPGPDGAKGEQGPQGKRGAPGPKGPKGDQGGLQGPQGPRGDPGPTGQKGDR

GSPGEPGQTGGQGQQGFQGGEKGAAGDKGGVGDKGPGPVGSTGEPGGKGPAGHEGGATGP
RGSPGNKGPDGDKGEAGNPGPAGPPGPVITPTFYRG
>Petrosia_ficiformis|Pfic_m.16910_spongin_short-chain_collagen
GITGELGYPGEHGLRGQPGIRGLQGPPGPPGIVGSKGEIGYHGEPGALGQKGIKGDVGPP
GKQGIQGPMGPRG--------------------GGPPGTLGEPGTDGKQGHRGEMGERGI
KGAMGKKGPPGQKGYPGMKGIVGLQGRTGPPGHKG---HQGYPGQMGKRGGVGGPPGPEG
DPGRPGIPGEQGIEGDEGPIGEQGPPGLIGPPG--QGVRGPMGPRGPPGPKGFEGFPGDQ
GNKGGPIGRQGQ---------KGDIGDRGDPGVEG---YPGFDGQPAEDGENGEDGIPGE
PGP---KGEKGPPGEQGPPGSNGKPGIPGETGNPGLPGHPGVRGPKGYAGYKGYNG----
--------RVGGITGPPGLPGNKGPPGPPGIGGNKGIKGAKG------------------
-KIGPAGRKGIRGSKGDFGPPGQPGHPGMPGSLGLDGMDGNIGAPGPKGNKGGMPGIKGP
LGNPGPIGATGINGKHGLPGEAGKTGPTGAVGLAGIPGLKGGARGAPGGKGPDGKLGLPG
IRGKDSIQGPQGKRGPPGPTGLPGGFSGHKGFPGYKGAKGPKGFKGESGIKGKTG-----

-------IQGPQGAFGPT---------GEKGNQ-----GEQGLIGRKGLSGDRGEKGLRG
HRGNPGLPGPKGIAAINVR---

Silicatein

>Amphimedon_queenslandica|PAC:15726022_silicatein
FHEEWLLWKEKHGKVYPHGEEESKRLNIWLENKNYIEEHNQKAHVHGFTLKMNHFGDLTI
EEYRQSRPEEVDWRTKNAVTGVKDQGQCGSCYAFSAVGALEGAQALAHDKLVHLSEQNIV
DCSIPYGNKGCNGGNMYESFRYIIDNDGIDREDGYKYTGRQGQCGGRQVGIIHIPTGSEA
ELQSALATAGPVSVAIDGSSNAFRFYEKGVFDEPNCSTTKLTHAGLIIGYGKKKGKPYWL
VKNSWGPHWGMKGYIMMARNKANQCGIATAASFPTL
>Ephydatia_muelleri|Ephy113585_silicatein
FVEEWHLWKGQHQKSYMSELEELERHTIWLSNKKYIEEHNARSDDIGYTLAMNHFGDLTT
EEYNEIYVDSIDWRTKGAVTSVKYQGQCGASYAFATTGALEGASALASDKQVILSEQNII
DCSVPYGNHGCSGGDTYTAMKYVIDNGGIDTESSYSFQGKQSSCGASATGVISIASGSET
DLLAAVATVGPVAVAVDANTNAFRFYQSGVFDSSSCSSTKLNHAMLVTGYGSYNGKDYWL
VKNSWSKNWGDNGYILMVRNKYNQCGIATDALYPTL
>Haliclona_amboinensis|Hamb_m.346_silicatein
FPAKWHSWKLENNKAYSSLHEELERHITWLSNQVYIDAYNSKEDVFGFKLEMNEFGDLSH
FEFQEKYPESVDWRSMGAVSSVKSQGKCCSCYAFGALGTLEGVHALASGKLTELSAQNIL
DCSRVYGNRGCRRGSVENTYLYIIDNNGVDTKKSYPYLGKQTTCGATMHYYVQIEEGEEK
DLEAAVATQGPISVLVDASHNIFRFYKSGVLNIPNCSRTNVTQALTLIGYGTYNGKKYWL
VKNSWGKSWGIKGFAMLSRGKYNQCGIATHATFPSL
>Haliclona_tubifera|Htub_m.13405_silicatein
IDSEWLGWKKEHSRAYETDIHELERYVTWKSNKAYIEAHNQLRDQFGFHLALNQFGDLSS
NEYYYA-PVEVDWRTMGAVTGVKDQGRCHSCYAFAVTGALEGMAALATGNLASMSEQQIV
DCSVIYGNQGCSGGSREVALLYIVDHNGISTSEDYPYIGYQYLCGTRTTGMVKVTKGSED
DLMAAVAIAGPVTVGVDHLHSSFQFYAGGIFDEPSCSYTKLTHSMLIIGYGTSNGKDYWL
IKNSWGTTWGDNGYIKMVRGKYNQCGIASRAIYPST
>Leucosolenia_complicata|88120|lcgid8538_silicatein
LVSEWSEYKMEHGKQYDENVEDDRRFQLWKKALDLIEKFNAGN--ESFTLGMNQFGDMTD
EEYKQYLPETVDWRPKGYVTPVKNQAACGSCWAFSTTGSVEGQHFKKTGKLVSLSEQNLV
DCSKAEGNNGCEGGLMDFGFEYIQKNGGIDTEPSYPYTAKDGTCGATVSGHTDVKKESES
DLQEAVATVGPISVAIDASSSSFRYYKSGVFYHLFCSHTRLDHGVLAVGYGADSGKDYWL
VKNSWGASWGQEGYIMMSRNRNNNCGIATSASYPSV
>Oscarella_carmela|Ocar306089_silicatein
FRAEWQEWKATHGKKYNSDDEDMNRFEVWVSNLDYVNKWNAEE--HSFTLGMNEYSDMTE
DEFRTAFPDSVDWRTEGYVTEVKNQGNCGSCWAFSTTGSLEGQHFKKTNKLVSLSEQNLV
DCSKKEGNDGCEGGLMEQGFEYIKVNGGIDTEESYPYKAKDGHCGATCSGCVKVKAKSEE
QLQQAVASVGPISIAMDAHLRSFMMYKQGIYFDKKCSSTKLDHGVLAVGYGTQGADDYWI

VKNSWGKSWGMKGFFMIARNKDNACGIATDASYPVV
>Petrosia_ficiformis|Pfic_m.3497_silicatein
FHEEWMLWKDIHGKEYSSKE-ELQKHNVWLQNKNYIDEHNAMSDVHGFTLKMNHLGDLTD
DEYCSQYPEVVDWRTKNAVTDVKDQGQCGACYAFSAAGALEGAHALAHDELMRLSEQNIV
DCSVPFGNHGCNGGNMYDVFQYVIDNNGLDTEDSYPYQGKQGQCGASETGIIEIPPR-EA
DLQAAVATGGPIAVAIDGSSNAFRFYEKGVFDEPNCSSAKLTHAVLLIGYGNSNGKPYWL
VKNSWGPNWGMHGYIMMAKDKSNQCGIATDASFPTL
>Sycon_ciliatum|84648|scgid26406_silicatein
TDQRWQRFKAQYNKAYAPGE-EQVRFVYFQENIAFIDEQNAVG--HNYTLEENQFADLSD
NEFTTYYPAEVDWRSKGYVTPIKNQGACGSCWAFSATGSLEGQHFKKSGTLVSLSEQNLV
DCSTKEGDHGCGGGLMDFAFKYVEKNGGIDTEASYPYKAKNGKCGATCTGKKDIKRGNED
DLQQAVADIGPISVGIDASTKTFRFYRKGVMDDKQCSSTKLDHGVLAVGYGTDSGDDYWL
VKNSWGKSWGMEGYVMMSRNKDNQCGIATSASYPLV
>Tethya_aurantia|113585_silicatein
FKEEWQLWKKQHDKSYSTNLEELEKHLVWLSNKKYIELHNANADTFGFTLAMNHLGDMTD
HEYKERYPETVDWRTKGAVTGIKSQGDCGASYAFSAMGALEGINALATGKLTYLSEQNII
DCSVPYGNHGCKGGNMYVAFLYVVANEGVDDGGSYPFRGKQSSCGASMSGSVQINSGSES
DLEAAVANVGPVAVAIDGESNAFRFYYSGVYDSSRCSSSSLNHAMVITGYGISNNQEYWL
AKNSWGENWGELGYVKMARNKYNQCGIASDASYPTL

Spherulin

>Amphimedon_queenslandica|XP_011409904.1_spherulin
LKVGIYNSIPDDGFDMLEIDTISLPGLVPEALDAVQYEDTYYAYPTLLCGNFLINDIYGY
YLPDMYVDGYIDMYGSQKAQEAVAMIDFITFFTSQSLRESILFGEDRYLLQANKQVY
>Astrosclera_willeyanagi|AEA86515.1_spherulin
LRVGIYNCIPDDGFDMIEMDTANLKEVVPAAVGAAAINGKLYAYPTLLCGNFLINDDYGW
YLPYLYLDGYIDIHGRESVDKAVAIIDFVNYFLNNNLREDIAMGVDRYLLQSTETFY
>Candidatus_Thiomargarita_nelsonii|KHD05285.1_spherulin
LRVSLYPWVPDDAQHLIEIDTIILGTLVPVAAEAVTFNNRVYGVPHWTCGYFVIDLDGSW
DSVMVYLDAYRDTYPSRNLLNVVAATTFASYYLSDEVFEVVLMGLDRYLLPSTQTAF
>Ephydatia|Ephy4640_spherulin
LRVLIFPYIPDSAFDVAEVDMILLGEMAPFAVNAVTYGGTTYGIPTLVCANFLANFRGSW
TLPGYYLSAYVNAYGASFMYEGVAIKRFMEFYTSDDFRQSYAFCGDRYVLPANTNFY
>Haliclona_amboinensis|Hamb_m.6638_spherulin
LKVGIYNSIPDDGFDMLEIDTTSLKGLVPSAVQAVLVNGKLYAYPTLLCGNFLINDADGY
YLPDLYIDGYIDIHGASTAQDAVIMMEFVAYFASKSLRTNIALGSDRYLLQANEEVY
>Haliclona_tubifera|Htub_m.24398_spherulin
LKVGIYPWIPDDSFDILEMDTVLLGEVAQVAVDGVRYKDVHYGVPTQICGNFLIEFRGKW
ALPCFYLDAYIDKHGKDSAQEGVAVDKFIEFYTSLRFRNKYAVGKDRYIMIARKDFY
>Sorangium_cellulosum|WP_044988281.1_spherulin
LRVPLYPYIPDCSVDVIETDMILLGELVPAGIEASTYDGEIYGVPHWLCGHFTFNLLGSW
NLPSLYLDAWADTNGPEGVASAIAAAAFVAYMSSASTFAWILASEDRYLMAATFDAY
>Stigmatella_aurantiaca|WP_002616213.1_spherulin
LRVPLYPYIPDCAYDVVETDTVILRELVPAGLAASRQQQSTYGVPHWLCGDFIINLLGSW
NLPALYLDAWADRNGSANVASAVAALAFVDYMSQPSTFEWILMSEDRYLLPATLDSY

References

Eddy, S. R. (1998). Profile hidden Markov models. *Bioinformatics, 14*(9), 755-763.

Finn, R. D., Bateman, A., Clements, J., Coggill, P., Eberhardt, R. Y., Eddy, S. R., . . . Punta, M. (2014). Pfam: the protein families database. *Nucleic Acids Res, 42*(Database issue), D222-230. doi: 10.1093/nar/gkt1223

Hemmrich, G., & Bosch, T. C. (2008). Compagen, a comparative genomics platform for early branching metazoan animals, reveals early origins of genes regulating stem-cell differentiation. *BioEssays, 30*(10), 1010-1018. doi: 10.1002/bies.20813
